# Supplementary material for: Targeted therapies for previously treated advanced or metastatic renal cell carcinoma: systematic review and network meta-analysis
Source: BMJ Open. 2019 Mar 1;9(3):e024691. doi: 10.1136/bmjopen-2018-024691 (PMC6429896; doi:10.1136/bmjopen-2018-024691)
Supplement: Supplementary data [file bmjopen-2018-024691supp001.pdf]

SUPPLEMENTARY FILE

Targeted therapies for previously treated advanced or metastatic renal cell carcinoma: systematic review and network meta-analysis

Contents

SEARCH STRATEGY 2

DATA EXTRACTION 4

RISK OF BIAS SUMMARIES 7

DATASETS 9

WINBUGS CODE 12

RESULTS 14

REFERENCES 15

Targeted therapies for previously treated advanced or metastatic renal cell carcinoma: systematic review and network meta-analysis

SEARCH STRATEGY

Table 1. Example search strategy (EMBASE update search for randomised controlled trials)

**OVID: EMBASE 1974 to July 03 (searched on 4th July 2017 from Week 3 2016 to Week 27 2017)**		
**#**	**Search Terms**	**Results**
1	Carcinoma, Renal Cell/	20712
2	(renal cell carcinoma$ or cell renal carcinoma$ or renal carcinoma$ or kidney carcinoma$ or kidney cell carcinoma$ or renal adenocarcinoma$ or kidney adenocarcinoma$ or adenocarcinoma$renal or adenocarcinoma$kidney$).mp.	66950
3	(hypernephroma$ or nephroid carcinoma$ or hypernephroid carcinoma$ or kidney hypernephroma$ or kidney pelvic carcinoma$ or kidney pyelocarcinoma$ or renal hypernephroma$ or grawitz tumo?r$ or renal cell neoplasm$ or renal cell cancer$ or renal tumo?r$ or carcinoma chromophobe cell kidney$ or chromophobe cell kidney carcinoma$).mp.	17922
4	kidney neoplasms/	10255
5	(cancer$ adj2 kidney$1).ti,ab.	5836
6	(neoplasm$1 adj2 kidney$1).ti,ab.	329
7	(neoplasm$1 adj2 renal).ti,ab.	2153
8	(cancer$ adj2 renal).ti,ab.	12586
9	(tumo?r$1 adj2 kidney$1).ti,ab.	4838
10	(tumo?r$1 adj2 renal).ti,ab.	14674
11	or/1-10	92199
12	(axitinib or inlyta or AG013736 or "AG 013736").mp.	3492
13	(sorafenib or nexavar or bay 43-9006 or bay 439006 or bay43-9006 or bay439006).mp.	23166
14	(sunitinib or sutent or pha 2909040ad or pha2909040ad or "su 010398" or "su 011248" or su 10398 or su10398 or su 11248 or su010398 or su011248 or su11248).mp.	18935
15	(everolimus or afinitor or certican or zortress or nvp-rad-001 or rad-001 or rad 001a or rad001 or rad001a or sdz rad).mp.	23010
16	(nivolumab or opdivo or ONO4538 or ONO 4538 or BMS936558 or BMS 936558 or MDX1106 or MDX 1106).mp.	4666
17	(temsirolimus or cci-779 or cell-cycle-inhibitor-779 or nsc 683864 or nsc683864 or torisel).mp.	7267
18	(bevacizumab or avastin or nsc 704865 or nsc704865 or anti-vegf or rhumab-vegf).mp.	50865
19	(armala or pazopanib or gw786034 or gw 786034 or sb 710468 or sb710468 or votrient).mp.	5612
20	or/12-19	99220
21	Clinical trial/	934498
22	Randomized controlled trial/	460454
23	Randomization/	74486
24	Single blind procedure/	28210
25	Double blind procedure/	140589
26	Crossover procedure/	52369
27	Placebo/	309726
28	Randomi?ed controlled trial$.tw.	162409
29	Rct.tw.	24817
30	Random allocation.tw.	1704
31	Randomly allocated.tw.	28013
32	Allocated randomly.tw.	2271
33	(allocated adj2 random).tw.	867
34	Single blind$.tw.	19692
35	Double blind$.tw.	180274
36	((treble or triple) adj blind$).tw.	721
37	Placebo$.tw.	257991
38	Prospective study/	388181

39	or/21-38	1782373
40	Case study/	48285
41	Case report.tw.	342862
42	Abstract report/ or letter/	1025233
43	or/40-42	1408381
44	39 not 43	1736655
45	11 and 20 and 44	3942
46	Animals/ not Humans/	1295518
47	45 not 46	3942
48	(editorial or letter).pt.	1521255
49	47 not 48	3868
50	limit 49 to em=201603-201727	327

DATA EXTRACTION

Table 2. Data extraction template

**Study or trial name:**			**Publication source**
**Full reference for all publications:**			
**Design**			
Study design			
Number of centres & Country/countries			
Recruitment dates			
Length of follow-up [include study start date, data cut-off and completion date]			
Source of funding			
Eligibility criteria (inclusion and exclusion)			
**Participants and treatment arms**	**Intervention:**	**Comparator:**	**Publication, data cut-off**
Intervention, method of delivery, dose and frequency			
Concomitant medication(s) or therapies			
Cross-over or post-study interventions allowed<br>Number of patients (%)			
Number of cycles<br>At least one dose reduction n (%)			
Treatment duration (and the data cut offs for each publication for the study)			
Number randomised			
Number who received study medication			
Number withdrawn/ discontinued and reasons<br>[give breakdown]			
Disease stage and/or metastatic disease<br>[give breakdown]			
Previous systemic therapy treatments, n (%) [give breakdown]			
Age, years: median (range)			
Ethnicity, n (%)<br>[give breakdown]			
Male, n (%)			
Performance status n (%)			

[give tool and breakdown]			
Reported subgroups	None reported		
**Reported outcomes**			
Primary outcome			
Secondary outcomes			
Outcomes and time points with data reported for subgroups of prior baseline therapies			
Outcomes and time points with data reported for subgroups of baseline prognostic scores (e.g. ECOG, MSKCC)			
**Results**	**Intervention**	**Comparator**	**Publication, data cut-off**
**PFS**			
HR (95% CI)			
HR (95% CI) for subgroups based on prior therapy:			
PFS, median (95% CI) months			
PFS, median (95% CI), months for subgroups based on prior therapy			
Number of progression events n (%)			
**Overall survival**			
HR, (95% CI)			
HR, (95% CI) for subgroups based on prior therapy			
Number of deaths, n (%)			
Median OS, months (95% CI)			
Median OS, (95% CI) months for subgroup based on prior therapy			
Number of deaths, n (%) for subgroups based on prior therapy			
**Response**			
Objective response, n (%)			
Complete response, n (%)			
Partial response, n (%)			
Stable disease, n (%)			
Progressive disease, n (%)			
Time to response, months (median [range])			
Duration of response, median (95% CI), months			
Other measures of response			
**HRQoL**			

[Scale 1] Mean end of treatment			
[Scale 1] Mean difference (95% CI)			
Completion rate			
**Adverse events (AE's)**			
N in safety analysis			
Total AE's (any Grade)			
Total AE's Grade ≥3			
[enter list of individual AEs]			

**Risk of bias assessment based (RCTs)**		
**Domain**	**Risk assessment**	**Comments**
Random sequence generation	[Low/High/Unclear]	
Allocation concealment	[Low/High/Unclear]	
Blinding (who [participants, personnel], and method)	[Low/High/Unclear]	
Other biases	[Low/High/Unclear]	
Progression-free survival		
-Blinding of outcome assessment	[Low/High/Unclear]	
-Incomplete outcome data	[Low/High/Unclear]	
-Selective reporting	[Low/High/Unclear]	
Overall survival		
-Blinding of outcome assessment	[Low/High/Unclear]	
-Incomplete outcome data	[Low/High/Unclear]	
-Selective reporting	[Low/High/Unclear]	
Response (partial response, disease stabilisation, progressive disease)		
-Blinding of outcome assessment	[Low/High/Unclear]	
-Incomplete outcome data	[Low/High/Unclear]	
-Selective reporting	[Low/High/Unclear]	
HRQoL		
-Blinding of outcome assessment	[Low/High/Unclear]	
-Incomplete outcome data	[Low/High/Unclear]	
-Selective reporting	[Low/High/Unclear]	
Adverse events		
-Blinding of outcome assessment	[Low/High/Unclear]	
-Incomplete outcome data	[Low/High/Unclear]	
-Selective reporting	[Low/High/Unclear]	

RISK OF BIAS SUMMARIES

Table 3. Summary of Cochrane risk of bias assessment for randomised control trials

Criteria		AXIS	Checkmate -025	HOPE 205	METEOR	RECORD-1
All outcomes	Random sequence generation	✓	✓	✓	✓	✓
	Allocation concealment	✓	✓	✓	✓	✓
	Blinding: participant/personnel	X	X	X	X	✓
**Outcome-specific**						
OS	Blinding: outcome assessment	✓	✓	✓	✓	✓
	Incomplete outcome data	✓	?	✓	?	✓
	Selective Reporting	✓	✓	✓	✓	✓
	Other Biases	?	?	?	?	?
PFS	Blinding: outcome assessment	✓	X	✓	✓	✓
	Incomplete outcome data	✓	✓	✓	?	✓
	Selective Reporting	✓	✓	✓	✓	✓
	Other Biases	**NA**	**NA**	?	**NA**	**NA**
ORR	Blinding: outcome assessment	✓	X	✓	✓	✓
	Incomplete outcome data	?	✓	✓	?	✓
	Selective Reporting	✓	✓	✓	✓	?
	Other Biases	**NA**	**NA**	?	**NA**	**NA**
HRQoL	Blinding: outcome assessment	X	X	**NA**	X	X
	Incomplete outcome data	✓	✓	**NA**	?	✓
	Selective Reporting	✓	✓	**NA**	✓	X
	Other Biases	**NA**	**NA**	**NA**	**NA**	**NA**
AE	Blinding: outcome assessment	X	X	X	X	✓
	Incomplete outcome data	✓	✓	✓	✓	✓
	Selective Reporting	✓	✓	✓	✓	✓
	Other Biases	**NA**	**NA**	?	**NA**	**NA**

Key: ✓, low risk; ?, unclear risk; X, high risk; NA, not applicable.
Abbreviations: OS, overall survival; PFS, progression-free survival; ORR, objective response rate; HRQoL, health-related quality of life; AE, adverse effects.

Table 4. Summary of ROBINS-I risk of bias assessments in non-randomised studies

	Guida 2017	Heng 2016	Iacovelli 2015	Lakomy 2017	SPAZO-2	Vogelzang 2016	Wong 2014
**Overall survival**							
Confounding	~	~	x	x	~	~	~
Selection	✓	✓	✓	✓	✓	✓	✓
Intervention classification	✓	✓	✓	✓	✓	✓	✓
Intervention deviations	✓	✓	✓	✓	✓	✓	✓
Missing data	✓	NI	✓	✓	✓	✓	x
Outcome measures	✓	✓	✓	✓	✓	✓	✓
Outcome reporting	✓	✓	✓	✓	✓	✓	✓
**Overall judgement**	x	~	x	x	~	~	x
**Progression-free survival**							
Confounding	x	~	-	x	~	~	~
Selection	✓	✓	-	✓	✓	✓	✓
Intervention classification	✓	✓	-	✓	✓	✓	✓
Intervention deviations	✓	✓	-	✓	✓	✓	✓
Missing data	✓	NI	-	✓	✓	✓	x
Outcome measures	x	x	-	x	x	x	x
Outcome reporting	x	x	-	x	x	x	x
**Overall judgement**	x	x	-	x	x	x	x

Abbreviations: PFS = progression-free survival; OS = overall survival

Key: ✓, low risk; ~, moderate risk; x, serious risk; x, critical risk; NI, no information.

DATASETS

Table 5. Study data: overall survival

Study	Data details	T1	T2	HR (95% CI)	T1 median months (95% CI)	T2 median (95% CI)
**AXIS[1]**	**Prior sunitinib subset**	**5**	**6**	**1.00 (0.78 to 1.27)**	**NR**	**NR**
**CheckMate 025[2]**	**-**	**3**	**1**	**0.73 (0.57 to 0.93)**	**25.0 (21.8 to NE)**	**19.6 (17.6 to 23.1)**
**HOPE 205[3]**	**July 2015 cutoff**	**7**	**1**	**0.59 (0.36 to 0.97)**	**25.5 (16.4 to 32.1)**	**15.4 (11.8 to 20.6)**
**METEOR[4]**	**31 Dec 2015 cutoff**	**2**	**1**	**0.66 (0.53 to 0.83)**	**21.4 (18.7 to NE)**	**16.5 (14.7 to 18.8)**
**RECORD-1[5]**	**RPSFT adjusted**	**1**	**4**	**0.60 (0.22 to 1.65)**	**14.4 (NR)**	**10.0 (NR)**
Guida 2017[6]*		5	1	1.33 (0.8 to 2.1)	14.9 (7.4 to 22.4)	21.5 (16.5 to 26.5)
Heng 2016[7]	-	6	1	1.25 (0.75 to 2.10)	18.7 (NR)	23.0 (NR)
Heng 2016[7]	-	5	1	1.22 (0.77 to 1.94)	23.5 (NR)	23.0 (NR)
Iacovelli 2015[8]	-	6	1	2.21 (1.47 to 3.31)	NR	NR
SPAZO-2[9]	Adjusted results	5	1	0.81 (0.60 to 1.20)	11.6 (7 to 16)	9.5 (7 to 12)
Vogelzang 2016[10]	-	1	5	1.16 (0.74 to 1.82)	NR	NR
Wong 2014[11]	Full adjusted results	1	6	0.66 (0.44 to 0.99)	19.0 (NR)	13.8 (NR)

Abbreviations: T1, treatment 1; T2, treatment 2 (baseline); HR, hazard ratio; CI, confidence interval; NE, not estimable; RPSFT, rank preserving structural failure time model; NR, not reported.
Treatment codes: 1, everolimus; 2, cabozantinib; 3, nivolumab; 4, best supportive care/placebo; 5, axitinib; 6, sorafenib; 7, lenvatinib with everolimus. Studies in bold formed the primary analysis.
*Data from personal communication with the study author, 11 March 2018

Table 6. Study data: progression-free survival

Study	Data details	T1	T2	HR (95% CI)	T1 median months (95% CI)	T2 median (95% CI)
**HOPE 205[12]**	**IRR**	**7**	**1**	**0.45 (0.27 to 0.79)**	**12.8 (7.4 to 17.5)**	**5.6 (3.6 to 9.3)**
**METEOR[4]**	**IRR for ITT**	**2**	**1**	**0.51 (0.41 to 0.62)**	**7.4 (6.6 to 9.1)**	**3.9 (3.7 to 5.1)**
**RECORD-1[13]**	**Final analysis, ICR**	**1**	**4**	**0.33 (0.25 to 0.43)**	**4.9 (4.0 to 5.5)**	**1.9 (1.8 to 1.9)**
AXIS[1]	Prior sunitinib subset	5	6	0.72 (0.57 to 0.90)	6.5 (5.7 to 7.9)	4.4 (2.9 to 4.7)
Guida 2017[6]*		5	1	0.84 (0.55 to 1.2)	7.7 (5.3 to 10.2)	5.3 (4.0 to 6.6)
Heng 2016[7]	-	6	1	1.47 (0.95 to 2.28)	-	-
Heng 2016[7]	-	5	1	1.26 (0.81 to 1.95)	-	-
Iacovelli 2015[8]	-	6	1	NR	NR	NR
SPAZO-2[9]	Adjusted results	5	1	0.76 (0.5 to 1.1)	5.3 (3 to 7)	4.6 (3 to 6)

Vogelzang 2016[10]	Adjusted results	1	5	1.16 (0.85 to 1.59)	NR	NR
Wong 2014[11]	Adjusted results	1	6	0.76 (0.55 to 1.04)	10.1 (NR)	8.6 (NR)

Abbreviations: T1, treatment 1; T2, treatment 2 (baseline); HR, hazard ratio; CI, confidence interval; NR, not reported.
Treatment codes: 1, everolimus; 2, cabozantinib; 3, nivolumab; 4, best supportive care/placebo; 5, axitinib; 6, sorafenib; 7, lenvatinib with everolimus. Studies in bold formed the primary analysis.
*Data from personal communication with the study author, 11 March 2018

Table 7. Study data: objective response rate

			N		Objective response	
**Study**	**T1**	**T2**	**T1**	**T2**	**T1**	**T2**
AXIS[14]	5	6	361	362	70	34
CheckMate 025[2]	3	1	410	411	103	22
HOPE 205[12]	7	1	51	50	18	0
METEOR[4]	2	1	330	328	57	11
RECORD-1[13]	1	4	277	139	5	0

Abbreviations: T1, treatment 1; T2, treatment 2 (baseline).
Treatment codes: 1, everolimus; 2, cabozantinib; 3, nivolumab; 4, best supportive care/placebo; 5, axitinib; 6, sorafenib; 7, lenvatinib with everolimus

Table 8. Study data: grade 3 or 4 adverse events

			N		Grade 3 or 4 adverse events	
**Study**	**T1**	**T2**	**T1**	**T2**	**T1**	**T2**
AXIS[1]	5	6	359	355	NR	NR
CheckMate 025[2]	3	1	406	397	76	145
HOPE 205[15]	7	1	51	50	36	25
METEOR[4]	2	1	331	322	235	193
RECORD-1[13]	1	4	274	137	NR	NR

Abbreviations: T1, treatment 1; T2, treatment 2 (baseline); HR, hazard ration; CI, confidence interval; NE, not estimable; RPSFT, rank preserving structural failure time model; NR, not reported.
Treatment codes: 1, everolimus; 2, cabozantinib; 3, nivolumab; 4, best supportive care/placebo; 5, axitinib; 6, sorafenib; 7, lenvatinib with everolimus

Table 9. Study data: health-related quality of life (not meta-analysed)

**Study**	**T1**	**T2**	**Study analysis details**	**FKSI scales**	**EuroQol scales**	**EORTC QLQ-C30**
AXIS[16]	5	6	End of treatment MD (95% CI)	DRS: MD 0.12 (-0.45 to 0.69) p = 0.68;<br>FKSI-15: MD 0.35 (-0.63 to 1.34) p = 0.48	5D Index: MD 0.02 (-0.01 to 0.05) p = 0.19<br>VAS: -0.53 (-2.77 to 1.72) p = 0.65	NR

CheckMate 025[2]	3	1	Median change (range) at week 104	DRS:<br>niv -2 (-1 to 16)<br>evo 2 (-7 to 15)	NR	NR
HOPE 205	7	1	NA	NR	NR	NR
METEOR	2	1	NA	NR	NR	NR
RECORD-1[17]	1	4	Time to deterioration HR (95% CI); results favour placebo	DRS: HR 0.82, 95% CI 0.75 to 0.92, p = 0.001	NR	Global health status HR 0.85 (0.75 to 0.96) p = 0.006<br>Physical functioning HR 0.84 (0.75 to 0.94) p = 0.001
Abbreviations: FKSI-DRS = Functional Assessment of Cancer Therapy (FACT) Kidney Cancer Symptom Index; DRS = Disease-related Symptoms subscale of the FKSI-15; EQ-5D = European Quality of Life self-report questionnaire; VAS = visual analogue scale; EORTC QLQ = European Organization for the Research and Treatment of Cancer Quality of Life Questionnaire; MD, mean difference; CI, confidence interval; HR, hazard ratio; NA, not applicable; NR, not reported						

Targeted therapies for previously treated advanced or metastatic renal cell carcinoma: systematic review and network meta-analysis

WINBUGS CODE

CODE 1: Fixed effect log hazard ratio NMA for 2-arm studies (overall survival primary and progression-free survival primary)

```
model{
#Model for log-hazard ratios
for(i in 1:ndp){
            prec[i]<- 1/(se[i]*se[i])
                    lhr[i]~dnorm(md[i],prec[i])

#Fixed effect model for log hazard ratios
                    md[i] <- d[t[i]] - d[b[i]]

#Deviance residuals for data i
    dev[i] <- (lhr[i] - md[i])*(lhr[i] - md[i])/(se[i]*se[i])
          }
          resdev <- sum(dev[])

#Give priors for log hazard ratios
          d[1]<-0
          for (k in 2:nt){
          d[k] ~ dnorm(0,.001)
          }

#Rank the treatment effects (with 1=best) & record the best treatment
for(k in 1:nt){
          rk[k]<- rank(d[],k)
          best[k]<-equals(rk[k],1)
          }

#All pairwise log hazard ratios and hazard ratios
for (c in 1:nt-1){
          for (k in (c+1):nt){
                    lhzr[c,k] <- d[k] - d[c]
                    HR[c,k] <- exp(lhzr[c,k])
                    }
          }
}
```

CODE 2: Fixed effect odds ratio NMA (objective response rate analysis)

```
model{

for(i in 1:ns){

          delta[i,t[i,1]]<-0
          mu[i] ~ dnorm(0,.0001)

          for (k in 1:na[i]) {
                    r[i,t[i,k]] ~ dbin(p[i,t[i,k]],n[i,t[i,k]])
```

```
                    logit(p[i,t[i,k]])<-mu[i] + delta[i,t[i,k]]

          rhat[i,t[i,k]]<- p[i,t[i,k]] * n[i,t[i,k]]

          resdev[i,k]<- 2 * (r[i,t[i,k]] * (log(r[i,t[i,k]]) - log(rhat[i,t[i,k]])) + (n[i,t[i,k]] - r[i,t[i,k]]) * (log(n[i,t[i,k]] - r[i,t[i,k]]) -
log(n[i,t[i,k]] - rhat[i,t[i,k]])))
          }
          sumdev[i]<-sum(resdev[i,1:na[i]])

for (k in 2:na[i]) {
          delta[i,t[i,k]] <- d[t[i,k]] - d[t[i,1]]          # trial-specific LOR
          }
}

sumdevtot<- sum(sumdev[])

d[1]<-0
for (k in 2:nt){
          d[k] ~ dnorm(0,.0001)
          }

for (i in 1:ns) {
          mu1[i] <- mu[i] * equals(t[i,1],1)
          }

for (k in 1:nt) {
          logit(T[k])<- sum(mu1[])/nb +d[k]
          }

for (k in 1:nt) {
          rk[k]<-nt - rank(T[],k)
          best[k]<-equals(rk[k],1)
          }

for (c in 1:(nt-1)) {
          for (k in (c+1):nt) {
                    lor[c,k] <- (d[k] - d[c])
                    or[c,k]<-exp(lor[c,k])
                              }
                    }
}
```

CODE 3: Fixed effect log hazard ratio NMA to combine 2-arm and multi-arm studies (overall survival sensitivity and progression-free survival sensitivity)

```
model{

# Priors

   #On tx effect mean
   beta[1] < -0
   for (tt in 2:nt){
   beta[tt]~dnorm(0,1.0E-6)
   }

   #On individual study baseline effect
```

```
    for(ss in 1:ns){
    alpha[ss] ~ dnorm(0,1.0E-6)
    }

# Fit data
    for(ii in 1:ndp){
    mu[ii] < - alpha[t[ii]]*multi[ii] + beta[tx[ii]] - beta[b[ii]]
    prec[ii] < - 1/pow(se[ii],2)
    m[ii] ~ dnorm(mu[ii],prec[ii])
    }

# Calculate HRs
    for (hh in 1:nt) {
    hr[hh] < -exp(beta[hh])
    }

# Rank
    for (ll in 1:nt) {
                        rk[ll]<-rank(beta[],ll)
      best[ll] < - equals(rk[ll],1)
       }
    }
}
```

RESULTS

Table 10. Results of the RCT network meta-analyses for objective response rate, with 0.5 correction of 0 values

	Best supportive care	Lenvatinib+ everolimus	Nivolumab	Cabozantinib	Everolimus
Everolimus	0.24 (0.00 to 1.39)	91190 (9.30 to 34400)	6.23 (3.78 to 10.01)	6.61 (3.27 to 12.55)	-
Cabozantinib	0.04 (0.00 to 0.24)	14500 (1.36 to 5629)	1.06 (0.41 to 2.19)	-	0.15 (0.08 to 0.31)
Nivolumab	0.00 (0.00 to 0.24)	7.73 (1.46 to 5652)	-	0.94 (0.46 to 2.41)	0.16 (0.10 to 0.26)
Lenvatinib + everolimus	0.01 (0.00 to 0.04)	-	0.13 (0.00 to 0.68)	0.00 (0.00 to 0.74)	0.00 (0.00 to 0.11)
BSC	-	193 (24.99 to 4494382)	657000 (4.15 to 9580)	23.53 (4.10 to 30312)	4.02 (0.72 to 4826)

Abbreviations: BSC, best supportive care; OS, overall survival; PFS, progression-free survival
Results are odds ratios with 95% credible interval; odds ratios > 1 favour the treatment along the top row.

REFERENCES

1. Motzer RJ, Escudier B, Tomczak P, Hutson TE, Michaelson MD, Negrier S, et al. Axitinib versus sorafenib as second-line treatment for advanced renal cell carcinoma: overall survival analysis and updated results from a randomised phase 3 trial, 2013. Available from: http://onlinelibrary.wiley.com/o/cochrane/clcentral/articles/055/CN-00864055/frame.html. Date accessed.
2. Motzer RJ, Escudier B, McDermott DF, George S, Hammers HJ, Srinivas S, et al. Nivolumab versus Everolimus in Advanced Renal-Cell Carcinoma, 2015. Available from: http://onlinelibrary.wiley.com/o/cochrane/clcentral/articles/537/CN-01108537/frame.html. Date accessed.
3. Hutson TE, Dutcus CE, Ren M, Baig MA, Fishman MN. Subgroup analyses and updated overall survival from the phase II trial of lenvatinib (LEN), everolimus (EVE), and LEN+EVE in metastatic renal cell carcinoma (mRCC). Journal of Clinical Oncology Conference 2016; 34.
4. Choueiri T, Escudier B, Powles T, Tannir N, Mainwaring P, Rin iB. Cabozantinib versus everolimus in advanced renal cell carcinoma (METEOR): final results from a randomised, open-label, phase 3 trial. The Lancet Oncology 2016; 17: 917-27.
5. Korhonen P, Zuber E, Branson M, al. e. Correcting overall survival for the impact of crossover via a rank-preserving structural failure time (RPSFT) model in the RECORD-1 trial of everolimus in metastatic renal-cell carcinoma. J Biopharm Stat 2012; 22: 1258-71.
6. Guida A, Albiges L, Derosa L, Loriot Y, Massard C, Fizazi K, et al. Everolimus Versus Axitinib as Second-line Therapy in Metastatic Renal Cell Carcinoma: Experience From Institut Gustave Roussy. Clinical Genitourinary Cancer 2017; 15: e1081-e8.
7. Heng DY, Signorovitch J, Swallow E, Li N, Zhong Y, Qin P, et al. Comparative Effectiveness of Second-Line Targeted Therapies for Metastatic Renal Cell Carcinoma: A Systematic Review and Meta-Analysis of Real-World Observational Studies. PLoS ONE [Electronic Resource] 2014; 9: e114264.
8. Iacovelli R, Santini D, Rizzo M, Felici A, Santoni M, Verzoni E, et al. Bone metastases affect prognosis but not effectiveness of third-line targeted therapies in patients with metastatic renal cell carcinoma. Can Urol Assoc J 2015; 9: 263-7.
9. Arranz Arija J, Perez-Valderrama B, Rodriguez Sanchez A, Puertas Alvarez J, Pinto Marin A, Maximiano Alonso C, et al. SPAZO2 (SOGUG): Comparative effectiveness of everolimus (Ev) vs axitinib (Ax) as second-line after first-line pazopanib (1stPz) in metastatic renal carcinoma (mRC). Annals of Oncology 2017; 28 (Supplement 5): v313.
10. Vogelzang NJ, Pal SK, Signorovitch JE, Reichmann WM, Li N, Yang C, et al. Comparative effectiveness of everolimus and axitinib as second targeted therapies for metastatic renal cell carcinoma in the US: A retrospective chart review. Current Medical Research and Opinion 2016; 32: 741-7.
11. Wong MK, Yang H, Signorovitch JE, Wang X, Liu Z, Liu NS, et al. Comparative outcomes of everolimus, temsirolimus and sorafenib as second targeted therapies for metastatic renal cell carcinoma: A US medical record review. Current Medical Research and Opinion 2014; 30: 537-45.
12. Motzer RJ, Hutson TE, Ren M, Dutcus C, Larkin J. Independent assessment of lenvatinib plus everolimus in patients with metastatic renal cell carcinoma, 2016. Available from: http://onlinelibrary.wiley.com/o/cochrane/clcentral/articles/453/CN-01168453/frame.html. Date accessed.
13. Motzer RJ, Escudier B, Oudard S, Hutson TE, Porta C, Bracarda S, et al. Phase 3 trial of everolimus for metastatic renal cell carcinoma : final results and analysis of prognostic factors, 2010. Available from: http://onlinelibrary.wiley.com/o/cochrane/clcentral/articles/159/CN-00762159/frame.html. Date accessed.
14. Rini BI, Escudier B, Tomczak P, Kaprin A, Szczylik C, Hutson TE, et al. Comparative effectiveness of axitinib versus sorafenib in advanced renal cell carcinoma (AXIS): a randomised phase 3 trial, 2011. Available from: http://onlinelibrary.wiley.com/o/cochrane/clcentral/articles/402/CN-00804402/frame.html. Date accessed.
15. Motzer RJ, Hutson TE, Glen H, Michaelson MD, Molina A, Eisen T, et al. Lenvatinib, everolimus, and the combination in patients with metastatic renal cell carcinoma: a randomised, phase 2,open-label, multicentre trial. Lancet Oncology 2015; 16: 1473-82.

16. Cella D, Escudier B, Rini B, Chen C, Bhattacharyya H, Tarazi J, et al. Patient-reported outcomes for axitinib vs sorafenib in metastatic renal cell carcinoma: phase III (AXIS) trial. British Journal of Cancer 2013; 108: 1571-8.
17. Beaumont JL, Butt Z, Baladi J, Motzer RJ, Haas T, Hollaender N, et al. Patient-reported outcomes in a phase iii study of everolimus versus placebo in patients with metastatic carcinoma of the kidney that has progressed on vascular endothelial growth factor receptor tyrosine kinase inhibitor therapy, 2011. Available from: http://onlinelibrary.wiley.com/o/cochrane/clcentral/articles/925/CN-00800925/frame.html
