# Supplementary material for: Targeted therapies for previously treated advanced or metastatic renal cell carcinoma: systematic review and network meta-analysis
Source: BMJ Open. 2019 Mar 1;9(3):e024691. doi: 10.1136/bmjopen-2018-024691 (PMC6429896; doi:10.1136/bmjopen-2018-024691)
Supplement: Author's manuscript [file bmjopen-2018-024691.draft_revisions.pdf]

BMJ Open is committed to open peer review. As part of this commitment we make the peer review history of every article we publish publicly available.

When an article is published we post the peer reviewers' comments and the authors' responses online. We also post the versions of the paper that were used during peer review. These are the versions that the peer review comments apply to.

The versions of the paper that follow are the versions that were submitted during the peer review process. They are not the versions of record or the final published versions. They should not be cited or distributed as the published version of this manuscript.

BMJ Open is an open access journal and the full, final, typeset and author-corrected version of record of the manuscript is available on our site with no access controls, subscription charges or pay-per-view fees (http://bmjopen.bmj.com).

If you have any questions on BMJ Open's open peer review process please email editorial.bmjopen@bmj.com

BMJ Open

TARGETED THERAPIES FOR PREVIOUSLY TREATED ADVANCED OR METASTATIC RENAL CELL CARCINOMA: SYSTEMATIC REVIEW AND NETWORK META-ANALYSIS

Journal:	*BMJ Open*
Manuscript ID	bmjopen-2018-024691
Article Type:	Research
Date Submitted by the Author:	08-Jun-2018
Complete List of Authors:	Karner, Charlotta; British Medical Journal Technology Assessment Group (BMJ-TAG) BMA House Tavistock Square London, UK WC1H 9JR<br>Kew, Kayleigh; British Medical Journal Technology Assessment Group (BMJ-TAG) BMA House Tavistock Square London, UK WC1H 9JR<br>Wakefield, Victoria; British Medical Journal Technology Assessment Group (BMJ-TAG) BMA House Tavistock Square London, UK WC1H 9JR<br>Masento, Natalie; British Medical Journal Technology Assessment Group (BMJ-TAG) BMA House Tavistock Square London, UK WC1H 9JR<br>Edwards, S; British Medical Journal Technology Assessment Group (BMJ-TAG) BMA House Tavistock Square London, UK WC1H 9JR
Keywords:	metastatic renal cell carcinoma, network meta-analysis, overall survival, progression-free survival, systematic reveiw

SCHOLARONE™ Manuscripts

For peer review only

TARGETED THERAPIES FOR PREVIOUSLY TREATED ADVANCED OR METASTATIC RENAL CELL CARCINOMA: SYSTEMATIC REVIEW AND NETWORK META-ANALYSIS

Charlotta Karner PhD, Kayleigh Kew MA (Cantab), Victoria Wakefield MBChB, Natalie Masento PhD, Steven J. Edwards DPhil*

Affiliations: BMJ Technology Assessment Group (BMJ-TAG), BMA House, Tavistock Square, London, WC1H 9JP, UK (all authors)

*corresponding author: sedwards@bmj.com

For peer review only

Word count: 4227 (excluding title page, abstract, article summary, references, figures and tables)

Topic area: Oncology

Key words: metastatic renal cell carcinoma, network meta-analysis, overall survival, progression-free survival, systematic review

Checklists completed: PRISMA 2009, MOOSE

For peer review only - http://bmjopen.bmj.com/site/about/guidelines.xhtml

ABSTRACT

Objective

To compare the effectiveness and safety of treatments for advanced or metastatic renal cell carcinoma (amRCC) after treatment with vascular endothelial growth factor (VEGF)-targeted treatment.

Design

Systematic review and network meta-analysis of randomised controlled trials (RCTs) and non-RCTs. MEDLINE, EMBASE, and The Cochrane Library were searched up to January 2018. Data extraction and critical appraisals were conducted in duplicate.

Participants

People with amRCC requiring treatment after VEGF-targeted treatment.

Interventions

Axitinib, cabozantinib, everolimus, lenvatinib with everolimus, nivolumab, sorafenib and best supportive care (BSC).

Outcomes

Primary outcomes were overall survival (OS) and progression-free survival (PFS); secondary outcomes were objective response rate (ORR), adverse events, and health-related quality of life (HRQoL).

Results

Twelve studies were included (n = 5,144): five RCTs and seven non-RCTs. Lenvatinib with everolimus significantly increased OS and PFS over everolimus (HR 0.61, 95% Credible Interval [95%CrI]: 0.36 to 0.96 and 0.47, 95%CrI: 0.26 to 0.77, respectively) as did

For peer review only - http://bmjopen.bmj.com/site/about/guidelines.xhtml

cabozantinib (HR 0.66, 95%CrI: 0.53 to 0.82 and 0.51, 95%CrI: 0.41 to 0.63, respectively). This remained the case when non-RCT evidence was included. Nivolumab also significantly improved OS versus everolimus (HR 0.74, 95%CrI: 0.57 to 0.93). OS sensitivity analysis, including non-RCTs, indicates everolimus being more effective than axitinib followed by sorafenib. However, inconsistency was identified in the OS sensitivity analysis. PFS sensitivity analysis suggests axitinib is more effective than everolimus, which is more effective than sorafenib. The results for ORR supported the primary OS and PFS analyses. Nivolumab is associated with fewer grade 3 or 4 adverse events than lenvatinib with everolimus or cabozantinib. HRQoL could not be analysed due to differences in tools used.

Conclusions

Evidence suggest that lenvatinib with everolimus, cabozantinib and nivolumab are likely to be more effective in prolonging survival than axitinib, everolimus, sorafenib or BSC for people with amRCC subsequent to VEGF-targeted treatment.

Protocol registration: PROSPERO CRD42017071540

ARTICLE SUMMARY

Strengths and limitations of this study

- This review is highly relevant and timely as it includes all recently approved treatments and focuses on the effectiveness of these treatments when used after first line VEGF-targeted tyrosine kinase inhibitor (TKI) treatment, as recommended in European clinical guidelines.

For peer review only

For peer review only - http://bmjopen.bmj.com/site/about/guidelines.xhtml

- The review focuses on high quality RCT evidence, but inclusion of comparative observational evidence in sensitivity analyses enabled estimates for axitinib and sorafenib, which otherwise could not be connected in the network.
- The reliability of the results of this review is hampered by trial design limitations of some of the included studies: the proportional hazards assumption did not hold for PFS in the one trial including nivolumab, RCT data for axitinib and sorafenib were limited to a subgroup analysis conducted in one study which could only be compared to the other treatments by including observational studies, and the trial assessing lenvatinib with everolimus is a small phase II trial with an increased risk of a false positive result and of over estimating the effect size due to some differences in baseline characteristics and relatively low significance level (alpha 0.15).
- There were also some differences between the trials in the network in terms of baseline characteristics, number of prior VEGF targeted treatments, and trial blinding, but there were too few studies to explore the potentially treatment modifying effects of these differences.

For peer review only

BACKGROUND

Renal cell carcinoma (RCC) makes up 80–90% of new kidney cancers, which occur most commonly in men over 60 years.[1] Kidney cancers are among the most common cancers in Europe (age-standardised rates estimated at 17.2/100,000 males and 8.1/100,000 females).[2] Established factors associated with increased risk of advanced or metastatic RCC (amRCC) are smoking, obesity, hypertension, germline mutations and advanced kidney disease.[1]

Five-year survival for people diagnosed at stage I is 80%, decreasing to less than 10% for those diagnosed at stage IV.[1] The disease is often asymptomatic until later stages, and so most people are diagnosed with advanced disease, at which point the goal of treatment is to slow the cancer progression and treat symptoms.

Targeted treatments are designed to interrupt the biological pathways needed for the cancer to grow and spread. Since 2006, eight targeted treatments have been approved by the European Medicines Agency (EMA) for the treatment of amRCC, falling within three classes: mammalian target of rapamycin inhibitors (mTORis; everolimus), tyrosine kinase inhibitors (TKIs; sunitinib, pazopanib, axitinib, cabozantinib, lenvatinib [in combination with the mTORi everolimus] and sorafenib), and monoclonal antibodies (MABs; nivolumab).

The emergence of targeted treatments has changed the RCC treatment pathway substantially and targeted treatments have virtually replaced the use of cytokines in many European health systems.[3] As a result, published studies assessing second-line targeted agents in populations who received first-line cytokines, or indeed adjusted indirect

For peer review only

For peer review only - http://bmjopen.bmj.com/site/about/guidelines.xhtml

comparisons combining studies that enrolled those having received prior cytokines, have limited applicability to current practice because nearly all people now receive sunitinib or pazopanib (VEGFRs) at first-line. Sunitinib and pazopanib are the recommended first-line treatments in the latest RCC European Society for Medical Oncology (ESMO) clinical practice guidelines.[3] ESMO recommends axitinib, cabozantinib, sorafenib, everolimus, nivolumab, and lenvatinib with everolimus as treatment options from second line.[3]

Second-line practice patterns are not well established, partly because some treatments have only relatively recently been approved by the EMA.[4-6] Randomised controlled trials (RCTs), cohorts and patient registry data are emerging but head-to-head comparisons remain limited. Given the high cost of RCTs, and the number of treatments available for use at second line, it is unlikely that every treatment will ever be compared to every other treatement available. As such, adjusted indirect treatment comparisons are required to provide estimates beyond trial comparators to help establish an evidence based treatment sequence for amRCC. Before cabozantinib, nivolumab and lenvatinib with everolimus were approved, network meta analyses (NMAs) of RCTs or good quality observational cohorts favoured axitinib and everolimus over sorafenib, though primarily within populations who had received prior cytokines.[7-10] Two NMAs of RCTs comparing more recently approved drugs indicate that lenvatinib with everolimus or cabozantinib are likely to be the most effective option to extend overall survival (OS) and progression-free survival (PFS) in amRCC. However, neither study included all the relevant treatments and both NMAs combine evidence for people who had either received prior cytokines or VEGF-targeted agents, which resulted in an unreliable network as type of prior treatment has been shown to be a

For peer review only - http://bmjopen.bmj.com/site/about/guidelines.xhtml

potential treatment effect modifier.[11] In addition, prior cytokines are less relevant to clinical practice as most people receive a TKI first line (also the recommended first line in clinical guidelines).[3]

This systematic review is the first to include randomised and non-randomised evidence for all recently-approved treatments for amRCC, focusing specifically on the relevant population who have previously received a VEGF-targeted treatment. By doing so, the review aims to provide a full and clinically relevant assessment of treatment benefits and harms.

OBJECTIVE

To compare the safety and clinical effectiveness of targeted treatments for amRCC previously treated with VEGF-targeted therapy.

METHODS

Methods for the review are reported in more detail in the published protocol (CRD42017071540) and were based on the principles published by the National Health Service Centre for Reviews and Dissemination.[12] The review reported here is an update and extension of a project commissioned by the UK National Institute for Health Research (NIHR), registered as CRD42016042384. This review was reregistered and updated to make the results applicable outside the UK and to include treatments that have received European marketing authorisation subsequent to publication of the first iteration of the review. Patients were not directly involved in the development of this review.

Eligibility criteria

Study design

For peer review only - http://bmjopen.bmj.com/site/about/guidelines.xhtml

RCTs formed the basis of the primary analyses for all outcomes. As per the published protocol, comparative non-RCTs were included in sensitivity analyses for OS and PFS to provide a connected network for all interventions of interest. Preclinical studies, animal studies, narrative reviews, editorials, opinions and case reports were not eligible.

Population

Adults (18+ years) with a diagnosis of amRCC who had received previous treatment with a VEGF-targeted treatment.

Interventions

Interventions of interest were axitinib, cabozantinib, everolimus, lenvatinib with everolimus, nivolumab and sorafenib. Studies were included if they compared any of the listed interventions with each other, placebo or best supportive care (BSC). For the purposes of this review, placebo was assumed to be the equivalent of BSC. Studies comparing an intervention of interest with another treatment were only included if there were insufficient direct comparisons to provide a connected network that included all treatments of interest.

Outcomes

The primary outcomes were OS and PFS. Secondary outcomes were predefined as objective response rate (ORR), adverse events of Grade 3 and above (as defined by the Common Terminology Criteria for Adverse Events), and health-related quality of life (HRQoL).

Studies were excluded if none of the outcomes of interest were reported. Non-RCTs were only included if they reported OS or PFS in a way that could be incorporated into the NMA

For peer review only

For peer review only - http://bmjopen.bmj.com/site/about/guidelines.xhtml

(i.e. as a hazard ratio [HR] or where a HR could be estimated from a Kaplan-Meier curve with the number of people at risk).

Search and selection process

Electronic searches for the original project were run in January 2016 (for RCTs; MEDLINE, EMBASE and CENTRAL) and June 2016 (non-RCTs; MEDLINE and EMBASE), and subsequently extended to cover a new intervention (lenvatinib with everolimus) and updated to January 2018. Manual searches of conference proceedings and bibliographies of included studies and systematic review were also updated to January 2018. Searches combined terms for the interventions of interest with condition terms for RCC and the relevant design filter (RCT or observational; example strategy provided in the Supplementary file). No date or language restrictions were applied. Searches for non-randomised evidence were limited to interventions required to connect the network of treatments.

Unpublished and ongoing studies were identified by contacting experts in the field and searching ClinicalTrials.gov and the EU Clinical Trials Register.

Two reviewers screened all titles and abstracts independently. Full texts were retrieved and reviewed for records identified as potentially relevant by one or both reviewers. Discrepancies were resolved by consensus or by involving a third reviewer.

Data extraction and quality assessment

Data extraction was carried out independently by two reviewers and cross-checked for accuracy; as with study selection, discrepancies were resolved by discussion or by involving a third reviewer. A standard data extraction form was piloted and used to capture

For peer review only - http://bmjopen.bmj.com/site/about/guidelines.xhtml

information about study conduct, population, interventions, outcomes and risk of bias from each study, including the information source where more than one was available for a given study (template available in the Supplementary file together with extracted datasets for all outcomes). Where there were incomplete information study authors were contacted to gain further details.

Methodological quality was assessed independently by two reviewers using the Cochrane Risk of Bias tool for RCTs[13] and the ROBINS-I for non-randomised studies.[14] Where appropriate, risk of bias was assessed separately for each outcome within a study. Disagreements were resolved by consensus or by involving a third reviewer. The likely direction and magnitude of bias across the evidence as a whole was considered during interpretation of the results.

Data synthesis

Baseline characteristics of the included studies were compared to assess similarity of the study populations before combining results in an NMA. Fixed effects and random effects models were explored. However, as typically only one trial informed each pair-wise comparison and hence there were little data to inform the between trial heterogeneity, a pragmatic decision was made to use the fixed effects model for all outcomes. Statistical heterogeneity was assessed using the I^2 statistic for pairwise comparisons and deviance information criteria (DIC) for NMA. Inconsistency between direct and indirect effect estimates was assessed in closed loops in the network. Implications of observed clinical and statistical heterogeneity and inconsistency are described in the results.

For peer review only - http://bmjopen.bmj.com/site/about/guidelines.xhtml

Where NMA was possible, it was conducted according to the guidance described in the NICE Decisions Support Unit's Technical Support Documents for Evidence Synthesis.[15] A Bayesian Markov Chain Monte Carlo (MCMC) approach was taken in WinBUGS version 1.4.3 software[16] (codes included in the supplementary file) implementing uninformed priors and a burn-in of 30,000 iterations. Everolimus was specified as the baseline treatment. Data from multi-arm studies were adjusted to account for correlations in relative treatment effects.[17] OS and PFS were analysed as HRs, and response as odds ratios (ORs) using participants as the unit of analysis; no formal analysis could be performed for adverse effects or HRQoL due to between-study variation in reporting. A 95% Credible Interval can be interpreted as a 95% probability that the parameter falls within this range. If a 95% CrI doesn't include one this can, therefore, be interpreted as a statistically significant result (at the 5% level of significance). Primary analyses were based on studies of low, unclear or moderate risk of bias. Sensitivity analyses were planned for OS and PFS including RCTs of high risk of bias and non-RCTs of serious risk of bias. Non-RCTs at critical risk were excluded from all analyses.

RESULTS

Results of the searches

Results of the original and update search and selection process are shown in Figure 1.

The searches carried out in June 2016 led to the inclusion of 44 records relating to 12 studies. Five of these studies have been excluded from this review because of the update of the scope excluding sunitinib as it is not recommended at second line in the most up-to-date ESMO guidance for RCC.[3] Five new studies, one RCT and four retrospective chart

For peer review only - http://bmjopen.bmj.com/site/about/guidelines.xhtml

reviews, were identified in the update and extension searches (including terms for lenvatinib with everolimus) run in January 2018, making a total of 12 included studies.[4, 6, 10, 11, 18-25]

Included studies

Twelve studies (n = 5,144) met the inclusion criteria (Table 1): five RCTs (one double-blind[20] and four open-label[4, 6, 11, 19]); and seven non-RCTs[10, 18, 21-25] (retrospective cohort studies). Sample sizes varied from 101 (HOPE 205[6]) to 821 (CheckMate 025[26]) participants.

For peer review only

For peer review only - http://bmjopen.bmj.com/site/about/guidelines.xhtml

Table 1. Study characteristics

Study	Design	Location, funding	Prior treatments	Intervention	N	Type	Median age years	Male %	ECOG 0/1 %	Treatment duration (follow-up) months
AXIS[11]	PIII OL RCT	175 sites in 22 countries, Pfizer	1 prior systemic treatment (sunitinib, cytokine or other), prior sunitinib subgroup 54%	Axitinib	361	CC	61	73	99	8.2 (NR)
				Sorafenib	362		61	71	100	5.2 (NR)
CheckMate 025[26]	PIII OL RCT	146 sites in 24 countries, BMS	1 or 2 prior targeted treatments (TKI or other, no mTORi)	Nivolumab	410	CC	62	77	NR	5.5 (NR)
				Everolimus	411		62	74		3.7 (NR)
HOPE 205[6]	PII OL RCT	37 sites in Czech Republic, Poland, Spain, UK, US, Eisai	1 prior TKI, no prior mTORi	Lenvatinib+eve	51	CC	61	69	100	7.6 (NR)
				Everolimus	50		59	76	100	4.1 (NR)
METEOR[4]	PIII OL RCT	173 sites in 26 countries, Exelixis	1 or more prior TKIs; no prior mTORi	Cabozantinib	330	CC	63*	77	100	8.3 (18.7)
				Everolimus	328		62*	73	100	4.4 (18.8)
RECORD-1[20]	PIII DB RCT, Novartis	86 sites in Australia, Canada Europe, Japan, US, Novartis	1 or 2 prior TKIs; no prior mTORi	Everolimus	277	CC	61*	78	NR	4.6 (NR)
				BSC/placebo	139		60*	76		1.9 (NR)
Guida 2017[24]	Chart review	1 site in France, NR	1 prior targeted treatment (TKI or other)	Everolimus	81	92% CC	57	69	85	NR (33)
				Axitinib	45		54	78	82	NR (26)
Heng 2016[10]	Chart review	UK, Germany, France, Netherlands, Novartis	1 prior TKI (sunitinib or pazopanib)	Everolimus	115	NR	60.2	66.7	91.8% ≤ 2	NR (NR)
				Axitinib	96					NR (NR)
				Sorafenib	98					NR (NR)
Iacovelli 2015[18]	Chart review	23 sites in Italy, NR	2 prior targeted treatments (TKI or other)	Sorafenib	90	CC	63	74	81	NR (NR)
				Everolimus	143					NR (NR)
Lakomy 2017[25]	Chart	Czech national	1 prior targeted	Everolimus	520	94%	65	75	95	6.1 (NR)

For peer review only

For peer review only - http://bmjopen.bmj.com/site/about/guidelines.xhtml

	review	registry, **	treatment (TKI or other)	Sorafenib	240	CC	62	75	90	7.1 (NR)
SPAZO-2[23]	Chart review	50 sites in Spain, Novartis	1 prior TKI (pazopanib)	Everolimus	101	88% CC	66	64	NR	NR (28)
				Axitinib	88		63	68		
Vogelzang 2016[21]	Chart review	US, Novartis	1 prior TKI; no prior cytokines	Everolimus	325	85% CC	61*	70	80	NR (15*)
				Axitinib	127		60*	65	84	NR (13*)
Wong 2014[22]	Chart review	US, Novartis	1 prior TKI; no prior mTORi, cytokines, bevacizumab	Everolimus	233	91% CC	64	70	NR	NR (12.9)
				Sorafenib	123		66	72		NR (12.1)

Abbreviations: NR, not reported; RCT, randomised controlled trials; BSC, best supportive care; mRCC, metastatic renal cell carcinoma; amRCC, advanced or metastatic RCC; cc, clear cell variant; ncc, non-clear cell variant; TKI, tyrosine kinase inhibitor; mTORi, mammalian target of rapamaycin inhibitor. Notes: ECOG percentages that do not total 100 are due to missing data;
*mean values where median was not reported.
** Ministry of Health of the Czech Republic, Central European Institute of Technology, The Ministry of Education, Youth and Sports. RENIS registry part funded by Pfizer, Bayer and Novartis.

For peer review only - http://bmjopen.bmj.com/site/about/guidelines.xhtml

All studies recruited adults with amRCC who had received at least one prior VEGF-targeted treatment. AXIS[11] also included people who had not received prior anti-VEGF treatment, but OS and PFS data were available for the subset who had. In eight of the included studies people had only received one prior VEGF-targeted treatment;[5, 10, 21-25, 27] the remaining five studies allowed one or more prior treatments.[4, 18, 26, 28] Populations were predominantly male and Caucasian, and mean age was generally between 55 and 65 years. Where reported, most people had stage 3 or 4 clear-cell RCC and the majority had baseline ECOG performance status of 0 or 1. Baseline characteristics were generally well balanced between treatment groups within trials, with the exception of HOPE 205[5], in which there were some imbalances in baseline characteristics, which may favour lenvatinib with everolimus over everolimus.

Where dose was reported, it was started at the standard licensed dose and adjusted according to clinical judgement. Treatment was reported in the RCTs to be continued until disease progression, unacceptable toxicity or withdrawal of consent, except for METEOR[4] and CheckMate 025[26] in which people could be treated beyond progression. Median treatment duration in the five studies where it was reported varied from 1.9 months (placebo [BSC] group of RECORD-1[20]) to 8.3 months (cabozantinib group of METEOR[4]). Median length of follow-up ranged from 12.1 months to 23.6 months, but was only reported in four studies.

Most studies gave limited information regarding treatments received subsequent to the study drug. In RECORD-1,[20] 76% of people randomised to placebo received open-label everolimus at progression, but the confounding of OS was reduced by using crossover-

For peer review only - http://bmjopen.bmj.com/site/about/guidelines.xhtml

adjusted data in the NMA. Treatment crossover was not reported to have occurred in any other studies.

Treatments compared in each of the studies are shown in Table 1 and Figure 2. Direct comparisons made by RCTs are shown by black lines, and the additional connections possible by incorporating non-RCTs are shown with green lines; axitinib and sorafenib did not connect to the other treatments using only RCT evidence. Nivolumab could not be connected in the PFS network because it was not appropriate to analyse CheckMate 025[29] data with a Cox proportional hazards model.

Risk of bias

The five RCTs[4, 6, 11, 20, 29] were of good methodological quality; all are at low risk of bias for random sequence generation and allocation concealment. RECORD-1[20] was the only blinded study so there is a risk of performance bias in the others. In general, OS and PFS are considered low risk of detection and reporting biases for all RCTs except for a high risk of PFS detection bias in CheckMate 025[26] because it was not assessed by an independent review committee. None of the outcomes in the RCTs were at high risk of attrition bias; all used appropriate censoring for the time-to-event analyses, although OS data from CheckMate 025[26] and METEOR[4] are immature. Other possible sources of bias pertain to group differences in the rate and type of subsequent treatments received, which were poorly reported in most trials. RECORD-1[20] was the only trial allowing cross-over for people in the placebo arm, although cross-over adjusted results were reported. Despite appropriate randomisation in HOPE 205[6], which is a small phase II trial, there were some imbalances in

For peer review only - http://bmjopen.bmj.com/site/about/guidelines.xhtml

the baseline characteristics of the people in the trial, which may indicate a better prognosis for the lenvatinib with everolimus group compared with everolimus alone. In addition, alpha was set to 0.15, compared to the usual 0.05, and HOPE 205 is therefore of a higher risk of a false positive result and possibly of over estimating the effect size.

The non-RCTs included in the OS and PFS sensitivity analyses are at a higher risk of bias than the RCTs. Overall ROBINS-I ratings were at best moderate, for OS,[10, 21, 23] and serious risk of bias for PFS. One study was at critical risk of bias for both PFS and OS,[25] which was excluded from the sensitivity analyses. In all studies the potential for inadequate control for confounding was thought to increase the risk of bias. All studies reporting PFS also had an increased risk of bias for this outcome due to the lack of standardised measurement for assessing progression and that outcome assessors were aware of the interventions.

One of the observational studies was publicly funded,[25] two studies did not report their funding source[18, 24] and the remaining non-RCTs and all RCTs were sponsored by various pharmaceutical companies. Risk of bias assessments for all included studies are provided in the supplementary file.

Overall survival

Lenvatinib with everolimus, cabozantinib and nivolumab all showed statistically significant benefits over the baseline treatment, everolimus, in in the primary OS analysis (Table 2). Lenvatinib with everolimus had the highest probability (61%) of being the most effective treatment out of those compared in the primary analysis. These results were mirrored in the sensitivity analysis including non-RCTs. The sensitivity analysis also suggests everolimus may

For peer review only - http://bmjopen.bmj.com/site/about/guidelines.xhtml

be more effective than axitinib, sorafenib and BSC for overall survival. However, there is evidence of inconsistency between the direct and indirect evidence for axitinib, sorafenib and everolimus, which indicates that there is heterogeneity between the studies and highlights the uncertainty around the true estimates of the relative effect of these treatments.

Table 2. Results of the network meta-analyses for the primary outcomes (OS and PFS) and grade 3 or 4 adverse events

	**Primary NMA of RCTs**		**Sensitivity NMA of RCTs and non-RCTs**
**Overall survival**	Probability most effective (%)	HR *versus* everolimus (95% credible interval)	
Lenvatinib+everolimus	61	**0.61 (0.36 to 0.96)**	**0.61 (0.36 to 0.96)**
Cabozantinib	28	**0.66 (0.53 to 0.82)**	**0.66 (0.53 to 0.83)**
Nivolumab	10	**0.74 (0.57 to 0.93)**	**0.74 (0.57 to 0.93)**
Axitinib	-	-	1.14 (0.95 to 1.37)
Sorafenib	-	-	1.38 (1.12 to 1.68)
BSC	2	1.90 (0.61 to 4.53)	1.90 (0.60 to 4.56)
**Progression-free survival**	Probability best (%)	HR *versus* everolimus (95% credible interval)	
Lenvatinib+everolimus	67	**0.47 (0.26 to 0.77)**	**0.47 (0.26 to 0.77)**
Cabozantinib	34	**0.51 (0.41 to 0.63)**	**0.51 (0.41 to 0.63)**
Axitinib	-	-	0.84 (0.70 to 1.00)
Sorafenib	-	-	1.17 (0.95 to 1.43)
BSC	0	3.06 (2.31 to 3.97)	3.06 (2.31 to 3.97)
**Grade 3 or 4 adverse events**	Probability least harmful (%)	OR *versus* everolimus (95% credible interval)	
Lenvatinib+everolimus	0	2.67 (1.05 to 5.68)	-
Cabozantinib	0	1.66 (1.18 to 2.27)	-
Nivolumab	100	0.40 (0.29 to 0.55)	-

Progression-free survival

As with OS, lenvatinib with everolimus and cabozantinib both showed statistically significant benefits over everolimus, and lenvatinib with everolimus had the highest probability (66.5%) of being the most effective treatment out of those compared in the primary analysis of PFS

For peer review only - http://bmjopen.bmj.com/site/about/guidelines.xhtml

(Table 2). The results of the sensitivity analysis including non-RCT data indicate that axitinib also improves PFS compared with everolimus, whereas BSC leads to significantly shorter PFS compared with everolimus, and there was no statistically significant difference between everolimus and sorafenib. For PFS there was no evidence of inconsistency between the direct and indirect evidence of axitinib, sorafenib and everolimus.

Nivolumab was not included in the analyses of PFS because the proportional hazards assumption does not hold for this outcome in CheckMate 025.[26]

Objective response rate

Two of the four RCTs that could be included in the NMA for ORR observed no events in one treatment arm (everolimus in HOPE 205[6] and BSC in RECORD-1[20, 28]), causing the results from the NMA to be unreliable and lack face validity. Results using a 0.5 correction for 0 values indicate that treatment with cabozantinib, lenvatinib with everolimus, and nivolumab all lead to a better response rates than treatment with everolimus, which in turn in significantly better than BSC (supplementary file).

Adverse effects

In terms of safety, nivolumab had the highest probability of being least harmful, i.e. the rate of grade 3 or 4 AEs was significantly lower with nivolumab (18.7%) than with everolimus (36.5%),[26] whereas treatment with either cabozantinib or lenvatinib with everolimus resulted in significantly higher rates of grade 3 or 4 AEs than everolimus (METEOR[4]: cabozantinib 71.0%, everolimus 59.9%; HOPE 205[6]: lenvatinib + everolimus 71%, everolimus

For peer review only - http://bmjopen.bmj.com/site/about/guidelines.xhtml

50%). Rates of grade 3 or 4 AEs were not reported for axitinib or BSC in AXIS and RECORD-1.[27, 28]

Health-related quality of life

Treatments could not be compared using NMA for HRQoL as different measures and tools were used for assessments. HRQoL scores were similar between axitinib and sorafenib in AXIS[27] and results favoured nivolumab over everolimus in CheckMate 025.[26] Results in RECORD-1[20] favoured BSC over everolimus, although this effect was only apparent if models were used to account for data not missing at random. METEOR[4] results were similar for everolimus and cabozantinib. HRQoL was not measured in HOPE 205[6]. A summary of results from each of the five RCTs is provided in the Supplementary File.

DISCUSSION

This comprehensive review of the effectiveness and safety of all approved treatments for people with amRCC who has previously been treated with a VEGF-targeted treatment, suggests that for PFS and OS lenvatinib with everolimus is likely to be the most effective treatment, followed by cabozantinib and then nivolumab. However, nivolumab treatment may be associated with fewer grade 3 or 4 AEs than treatment with both lenvatinib with everolimus and cabozantinib. All treatments considered in this review appear to delay disease progression and prolong survival more than providing BSC. The results for ORR supported the primary OS and PFS analyses. Due to differences in reporting and HRQoL tools used, it was not possible to perform NMAs on safety or HRQoL.

For peer review only

For peer review only - http://bmjopen.bmj.com/site/about/guidelines.xhtml

This is a robust and comprehensive systematic review based on the principles published by Centre for Reviews and Dissemination[12] using the MOOSE[30] and PRISMA[31] reporting guidelines, and conducted according to prespecified methods in a prospectively registered protocol (PROSPERO CRD42017071540). The inclusion of all recently approved treatments increases the relevance and timeliness of the review. The review is also highly relevant as it focuses on the effectiveness and safety of these treatments when used after first line TKI treatment, as recommended in clinical guidelines.[3] However, there is not enough evidence available to answer questions about the sequencing of later lines of treatments.

Although this study focuses on high quality RCT evidence, the inclusion criteria were widened to incorporate comparative observational evidence in sensitivity analyses to enable estimates for axitib and sorafenib, which otherwise could not be connected to the network.

However, the robustness of the evidence in this review is limited by several factors:

1) PFS for nivolumab compared with the other treatments could not be estimated in this review because the proportional hazards assumption didn't hold for this outcome in the one trial including nivolumab.[26]
2) Relevant RCT data for axitinib and sorafenib were limited to a subgroup analysis conducted in one study that did not connect to the network of other RCTs.[27] Axitinib and sorafenib could only be compared to the other treatment options by including observational studies which were generally at a serious risk of different kinds of bias.
3) The trial assessing the efficacy of lenvatinib with everolimus is a small phase II trial, with an alpha set to 0.15 and therefore a higher than usual risk of false positive results and overestimation of the treatment effect. In this trial there were also some

For peer review only - http://bmjopen.bmj.com/site/about/guidelines.xhtml

differences in baseline characteristics likely to lead to an over estimation of the treatment effect of lenvatinib and everolimus compared with everolimus, which introduces uncertainty around the true treatment effect.

4) Although the baseline characteristics were well balanced within most of the trials, there were some differences in performance status and number of prior VEGF targeted treatments between the trials. There were also differences in trial design with some trials being double blind or open label. Outcome assessment was not always done by an independent review committee (IRC). However, in the nivolumab trial, CheckMate 025[26], progression was only assessed by non-blinded trial investigator. There were too few studies to explore the effects of these differences between studies, which is a limitation and increases the uncertainty of the results.

Although we can be confident that lenvatinib with everolimus, cabozantinib and nivolumab are effective treatments for prolonging PFS and probably also OS compared to everolimus, there is still considerable uncertainty around how they compare to each other and how much better they are than the first generation of targeted treatments, axitinib and sorafenib.

Two NMAs of different subsets of treatments for previously treated amRCC have recently been published.[32, 33] Unlike these studies, this review provides an alternative approach and a comparison between all recently approved treatments. Rassy *et al.*[33] and Amzal *et al.*[32] combine evidence for people who had either received prior cytokines or VEGF-targeted agents. This enabled a connected network using only RCT data, but type of prior treatment has been shown to be a potential treatment effect modifier,[27] which could introduce bias

For peer review only - http://bmjopen.bmj.com/site/about/guidelines.xhtml

into the analysis. In addition, results for people who have only had prior cytokines are less relevant to clinical practice than for prior VEGF-targeted treatments as most people receive a TKI first line, in line with clinical guidelines.[3] The NMAs of Amzal *et al.*[32] and Rassy *et al*.[33] are also limited by the reliance on the TARGET trial[34] to link axitinib and sorafenib to the network analysed. TARGET[34] is an RCT of sorafenib and placebo in which people only had prior cytokines and not prior TKI. The results from the TARGET trial are also confounded by crossover, which has only been partly accounted for by using immature data censored at crossover, and the lack of proportional hazards between the trial arms for PFS and OS.

For the trials that are shared between Amzal *et al*.[32] and this review and Rassy *et al*.[33] and this review the order, in terms of efficacy, is similar. However, this systematic review focuses specifically on the most relevant population, who have previously received a VEGF-targeted treatment, and avoids the issues with the TARGET[34] trial by including both randomised and non-randomised evidence, and thereby provides more relevant and reliable estimates of the relative efficacy between all the interventions.

All treatments considered in this review delay disease progression and prolong survival more than BSC, and although this review gives an indication of the ranking of the most effective treatments for treating recurrent amRCC there is still much uncertainty around how much these treatments differ from each other in terms of effectiveness and safety. The choice of treatment should take into account patient preference, comorbidities, symptoms, tumour burden and how aggressive the cancer is. Policy makers also need to consider the cost-effectiveness of the treatments.

For peer review only - http://bmjopen.bmj.com/site/about/guidelines.xhtml

It would be preferable to have high quality RCT data comparing all the available RCC treatment options, but this is unlikely to be commissioned due to the high costs of clinical trials. However, what is more likely and still needed is a larger RCT of lenvatanib with everolimus to confirm the efficacy data from the current phase II trial with its small sample size. RCT data of axitinib and sorafenib versus other comparators in the network are also required to enable higher quality evidence for these comparisons. As there is no cure for amRCC and as virtually all people progress, research is needed into the development of resistance to treatments. Further research is also required into the impact of different sequencing of drugs from second line and onwards as more people are well enough to tolerate additional lines of treatment and most of these drugs are approved for use also beyond second line (cabozantinib, everolimus, and nivolumab).

AUTHOR CONTRIBUTIONS

Charlotta Karner PhD validated data extraction, carried out and validated meta-analyses, and drafted and edited the manuscript. Kayleigh Kew MA (Cantab) carried out additional searches, contributed to the appraisal of title and abstracts, assessment of full publications for inclusion, data extraction and validation, carried out meta-analyses, and drafted the manuscript. Victoria Wakefield MBChB devised and carried out database searches, contributed to the appraisal of title and abstracts, assessment of full publications for inclusion, and data extraction. Natalie Masento PhD contributed to the appraisal of title and abstracts, the assessment of full publications for inclusion, and data extraction. Steven Edwards DPhil supervised the production of the manuscript and acted as methodological advisor.

For peer review only - http://bmjopen.bmj.com/site/about/guidelines.xhtml

ACKNOWLEDGEMENTS

The authors would like to thank Clare Fiatikoski (BMJ) and George Osei-Assibey (BMJ) for their contribution to the original literature searching and data extraction underpinning this review, and Samantha Barton (BMJ) for providing comments on the draft manuscript. The authors would also like to thank Dr Amit Bahl (University Hospitals Bristol NHS Foundation Trust, Bristol), Dr Lisa Pickering (St Georges Hospital, London), Dr Sarah Rudman (Guys and St Thomas Hospital, London), Dr Penny Kechagioglou (University Hospitals Coventry and Warwickshire, Coventry), Professor Martin Gore (Royal Marsden NHS Foundation Trust, London) and Dr Naveed Sarwar (Imperial College Healthcare NHS Trust, London) for providing clinical advice throughout the original project.

COMPETING INTERESTS

No competing interests were declared which affect the impartiality of this report. BMJ-TAG and the editorial team of *The BMJ* work independently to one another.

FUNDING

The review presents an update and extension to independent research funded by the UK National Institute for Health Research (NIHR) health technology assessment programme (HTA 16/58/01). The views and opinions expressed in this report are those of the authors and not necessarily those of the NHS, the NIHR or the Department of Health and Social Care.

FIGURE LEGENDS

Figure 1. PRISMA diagram

Abbreviation: RCT, randomised controlled trials

Figure 2. Network diagram

For peer review only - http://bmjopen.bmj.com/site/about/guidelines.xhtml

For peer review only

For peer review only - http://bmjopen.bmj.com/site/about/guidelines.xhtml

REFERENCE LIST

1. Cancer Research UK (CRUK). Kidney Cancer Statistics. *Available at:* http://wwwcancerresearchukorg/health-professional/cancer-statistics/statistics-by-cancer-type/kidney-cancer#heading-Zero *Accessed: 10 March 2016* 2015.
2. Ferlay J, Steliarova-Foucher E, Lortet-Tieulent J, Rosso S, Coebergh JW, Comber H, et al. Cancer incidence and mortality patterns in Europe: estimates for 40 countries in 2012. *European journal of cancer* 2013; **49**: 1374-403.
3. Escudier B, Porta C, Schmidinger M, Rioux-Leclercq N, Bex A, Khoo V, et al. Renal cell carcinoma: ESMO Clinical Practice Guidelines for diagnosis, treatment and follow-up. *Annals of oncology : official journal of the European Society for Medical Oncology / ESMO* 2016; **27**: v58 – v68.
4. Choueiri T, Escudier B, Powles T, Tannir N, Mainwaring P, Rin iB. Cabozantinib versus everolimus in advanced renal cell carcinoma (METEOR): final results from a randomised, open-label, phase 3 trial. *The Lancet Oncology* 2016; **17**: 917-27.
5. Motzer RJ, Escudier B, McDermott DF, George S, Hammers HJ, Srinivas S, et al. Nivolumab versus Everolimus in Advanced Renal-Cell Carcinoma. *New England Journal of Medicine* 2015; **373**: 1803-13.
6. Motzer RJ, Hutson TE, Glen H, Michaelson MD, Molina A, Eisen T, et al. Lenvatinib, everolimus, and the combination in patients with metastatic renal cell carcinoma: a randomised, phase 2,open-label, multicentre trial. *Lancet Oncology* 2015; **16**: 1473-82.
7. Larkin J, Paine A, Tumur I, Cappelleri JC, Healey PJ, Sr., Foley G, et al. Second-line treatments for the management of advanced renal cell carcinoma: systematic review and meta-analysis. *Expert Opin Pharmacother* 2013; **14**: 27-39.
8. Dranitsaris G, Schmitz S, Broom RJ. Small molecule targeted therapies for the second-line treatment for metastatic renal cell carcinoma: a systematic review and indirect comparison of safety and efficacy. *J Cancer Res Clin Oncol* 2013; **139**: 1917-26.
9. Wang H, Man L, Li G, Huang G, Wang J. Comparative efficacy and safety of axitinib versus sorafenib in metastatic renal cell carcinoma: a systematic review and meta-analysis. *Onco Targets Ther* 2016; **9**: 3423-32.
10. Heng DY, Signorovitch J, Swallow E, Li N, Zhong Y, Qin P, et al. Comparative Effectiveness of Second-Line Targeted Therapies for Metastatic Renal Cell Carcinoma: A Systematic Review and Meta-Analysis of Real-World Observational Studies. *PLoS ONE [Electronic Resource]* 2014; **9**: e114264.
11. Motzer RJ, Escudier B, Tomczak P, Hutson TE, Michaelson MD, Negrier S, et al. Axitinib versus sorafenib as second-line treatment for advanced renal cell carcinoma: overall survival analysis and updated results from a randomised phase 3 trial, 2013. Available from: http://onlinelibrary.wiley.com/o/cochrane/clcentral/articles/055/CN-00864055/frame.html. Date accessed.
12. Centre for Reviews and Dissemination (CRD). CRD's guidance for undertaking reviews in healthcare, 2011. Date accessed: 24 July 2017.
13. Higgins JP, Altman DG, Gotzsche PC, Juni P, Moher D, Oxman AD, et al. The Cochrane Collaboration's tool for assessing risk of bias in randomised trials. *Bmj* 2011; **343**: d5928.
14. Sterne JAC, Hernán MA, Reeves BC, Savović J, Berkman ND, Viswanathan M, et al. ROBINS-I: a tool for assessing risk of bias in non-randomized studies of interventions. *BMJ* 2016; **355**: i4919.
15. Dias S, Welton NJ, Sutton AJ, Ades AE. NICE DSU Technical Support Document 1: introduction to evidence synthesis for decision making. *Available at* http://wwwnicedsuorguk/TSD1%20Introductionfinal080512pdf *Accessed 7 March 2017* 2011 (updated 2012).
16. Lunn DJ, Thomas A, Best N, Spiegelhalter D. WinBUGS – A Bayesian modelling framework: Concepts, structure, and extensibility. *Statistics and Computing* 2000; **10**: 325-37.

For peer review only - http://bmjopen.bmj.com/site/about/guidelines.xhtml

17. Woods BS, Hawkins N, Scott DA. Network meta-analysis on the log-hazard scale, combining count and hazard ratio statistics accounting for multi-arm trials: a tutorial. *BMC Med Res Methodol* 2010; **10**: 54.
18. Iacovelli R, Santini D, Rizzo M, Felici A, Santoni M, Verzoni E, et al. Bone metastases affect prognosis but not effectiveness of third-line targeted therapies in patients with metastatic renal cell carcinoma. *Can Urol Assoc J* 2015; **9**: 263-7.
19. Motzer RJ, Escudier B, McDermott DF, George S, Hammers HJ, Srinivas S, et al. Nivolumab versus Everolimus in Advanced Renal-Cell Carcinoma, 2015. Available from: http://onlinelibrary.wiley.com/o/cochrane/clcentral/articles/537/CN-01108537/frame.html. Date accessed.
20. Motzer RJ, Escudier B, Oudard S, Hutson TE, Porta C, Bracarda S, et al. Efficacy of everolimus in advanced renal cell carcinoma: a double-blind, randomised, placebo-controlled phase III trial, 2008. Available from: http://onlinelibrary.wiley.com/o/cochrane/clcentral/articles/982/CN-00649982/frame.html. Date accessed.
21. Vogelzang NJ, Pal SK, Signorovitch JE, Reichmann WM, Li N, Yang C, et al. Comparative effectiveness of everolimus and axitinib as second targeted therapies for metastatic renal cell carcinoma in the US: A retrospective chart review. *Curr Med Res Opin* 2016; **32**: 741-7.
22. Wong MK, Yang H, Signorovitch JE, Wang X, Liu Z, Liu NS, et al. Comparative outcomes of everolimus, temsirolimus and sorafenib as second targeted therapies for metastatic renal cell carcinoma: A US medical record review. *Current Medical Research and Opinion* 2014; **30**: 537-45.
23. Arranz Arija J, Perez-Valderrama B, Rodriguez Sanchez A, Puertas Alvarez J, Pinto Marin A, Maximiano Alonso C, et al. SPAZO2 (SOGUG): Comparative effectiveness of everolimus (Ev) vs axitinib (Ax) as second-line after first-line pazopanib (1stPz) in metastatic renal carcinoma (mRC). *Annals of Oncology* 2017; **28 (Supplement 5)**: v313.
24. Guida A, Albiges L, Derosa L, Loriot Y, Massard C, Fizazi K, et al. Everolimus Versus Axitinib as Second-line Therapy in Metastatic Renal Cell Carcinoma: Experience From Institut Gustave Roussy. *Clinical Genitourinary Cancer* 2017; **15**: e1081-e8.
25. Lakomy R, Poprach A, Bortlicek Z, Melichar B, Chloupkova R, Vyzula R, et al. Utilization and efficacy of second-line targeted therapy in metastatic renal cell carcinoma: Data from a national registry. *BMC Cancer* 2017; **17 (1) (no pagination)**.
26. Motzer RJ, Escudier B, McDermott DF, George S, Hammers HJ, Srinivas S, et al. Nivolumab versus Everolimus in Advanced Renal-Cell Carcinoma. *N Engl J Med* 2015; **373**: 1803-13.
27. Motzer RJ, Escudier B, Tomczak P, Hutson TE, Michaelson MD, Negrier S, et al. Axitinib versus sorafenib as second-line treatment for advanced renal cell carcinoma: overall survival analysis and updated results from a randomised phase 3 trial. *The Lancet Oncology* 2013; **14**: 552-62.
28. Motzer RJ, Escudier B, Oudard S, Hutson TE, Porta C, Bracarda S, et al. Phase 3 trial of everolimus for metastatic renal cell carcinoma : final results and analysis of prognostic factors, 2010. Available from: http://onlinelibrary.wiley.com/o/cochrane/clcentral/articles/159/CN-00762159/frame.html. Date accessed.
29. Motzer R, Sharma P, McDermott D, George S, HJ H, Srinivas S, editors. CheckMate 025 phase III trial: Outcomes by key baseline factors and prior therapy for nivolumab (NIVO) versus everolimus (EVE) in advanced renal cell carcinoma (RCC). ESMO 2016; 2016; Copenhagen: Journal of Clinical Oncology.

For peer review only - http://bmjopen.bmj.com/site/about/guidelines.xhtml

30. Stroup DF, Berlin JA, Morton SC, Olkin I, Williamson GD, Rennie D, et al. Meta-analysis of observational studies in epidemiology: a proposal for reporting. Meta-analysis Of Observational Studies in Epidemiology (MOOSE) group. *Jama* 2000; **283**: 2008-12.
31. Moher D, Liberati A, Tetzlaff J, Altman DG, Group P. Preferred reporting items for systematic reviews and meta-analyses: the PRISMA statement. *Bmj* 2009; **339**: b2535.
32. Amzal B, Fu S, Meng J, Lister J, Karcher H. Cabozantinib versus everolimus, nivolumab, axitinib, sorafenib and best supportive care: A network meta-analysis of progression-free survival and overall survival in second line treatment of advanced renal cell carcinoma. *PLoS One* 2017; **12**: e0184423.
33. El Rassy E, Aoun F, Sleilaty G, Kattan J, Banyurwabuke B, Zanaty M, et al. Network meta-analysis of second-line treatment in metastatic renal cell carcinoma: efficacy and safety. *Future Oncol* 2017; **13**: 2709-17.
34. Escudier B, Eisen T, Stadler WM, Szczylik C, Oudard S, Siebels M, et al. Sorafenib in advanced clear-cell renal-cell carcinoma. *N Engl J Med* 2007; **356**: 125-34.

For peer review only

For peer review only - http://bmjopen.bmj.com/site/about/guidelines.xhtml

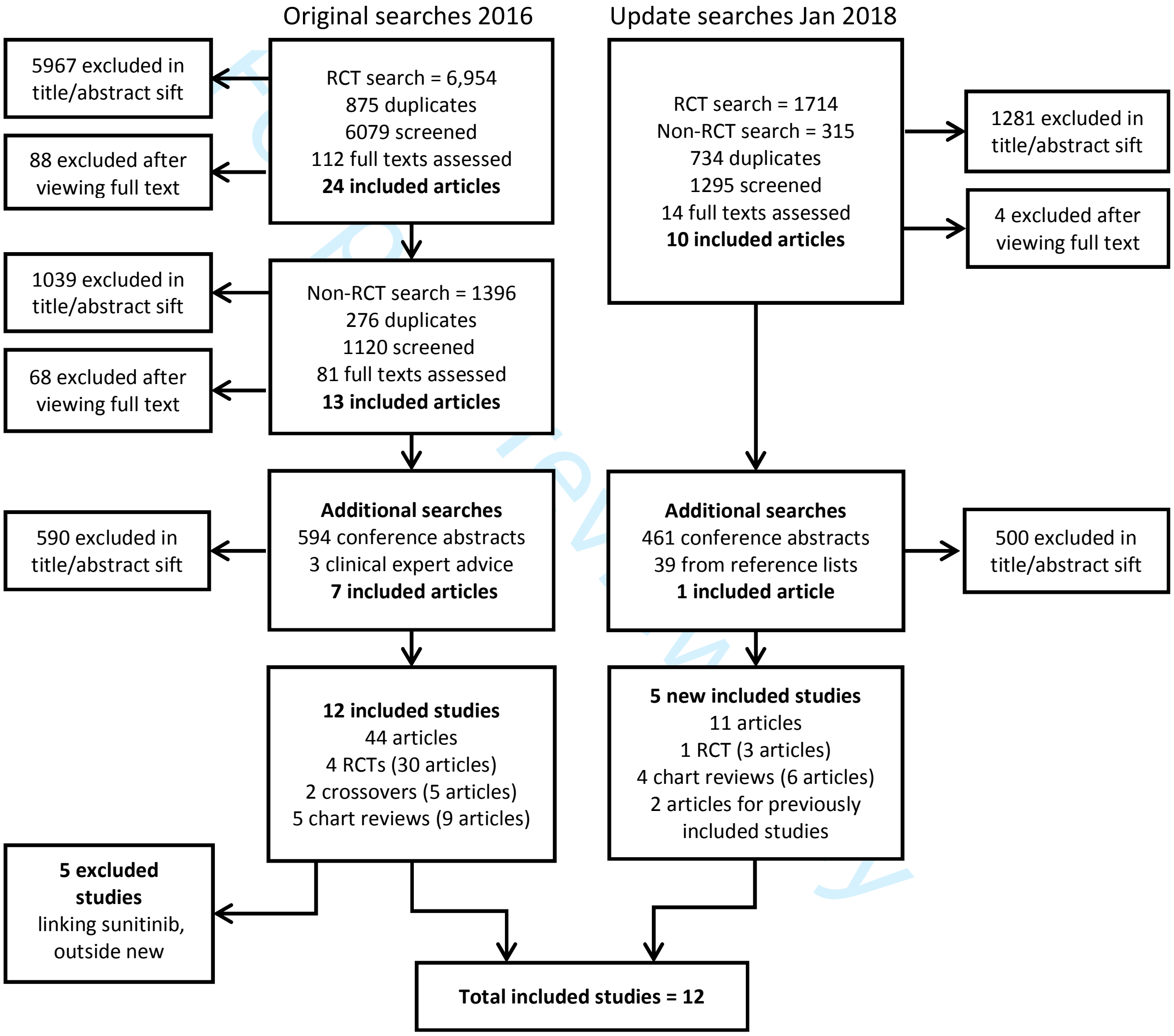
Original searches 2016
Update searches Jan 2018
5967 excluded in title/abstract sift
RCT search = 6,954
875 duplicates
6079 screened
112 full texts assessed
24 included articles
88 excluded after viewing full text
1039 excluded in title/abstract sift
Non-RCT search = 1396
276 duplicates
1120 screened
81 full texts assessed
13 included articles
68 excluded after viewing full text
RCT search = 1714
Non-RCT search = 315
734 duplicates
1295 screened
14 full texts assessed
10 included articles
1281 excluded in title/abstract sift
4 excluded after viewing full text
590 excluded in title/abstract sift
Additional searches
594 conference abstracts
3 clinical expert advice
7 included articles
Additional searches
461 conference abstracts
39 from reference lists
1 included article
500 excluded in title/abstract sift
12 included studies
44 articles
4 RCTs (30 articles)
2 crossovers (5 articles)
5 chart reviews (9 articles)
5 new included studies
11 articles
1 RCT (3 articles)
4 chart reviews (6 articles)
2 articles for previously included studies
5 excluded studies
linking sunitinib, outside new
Total included studies = 12

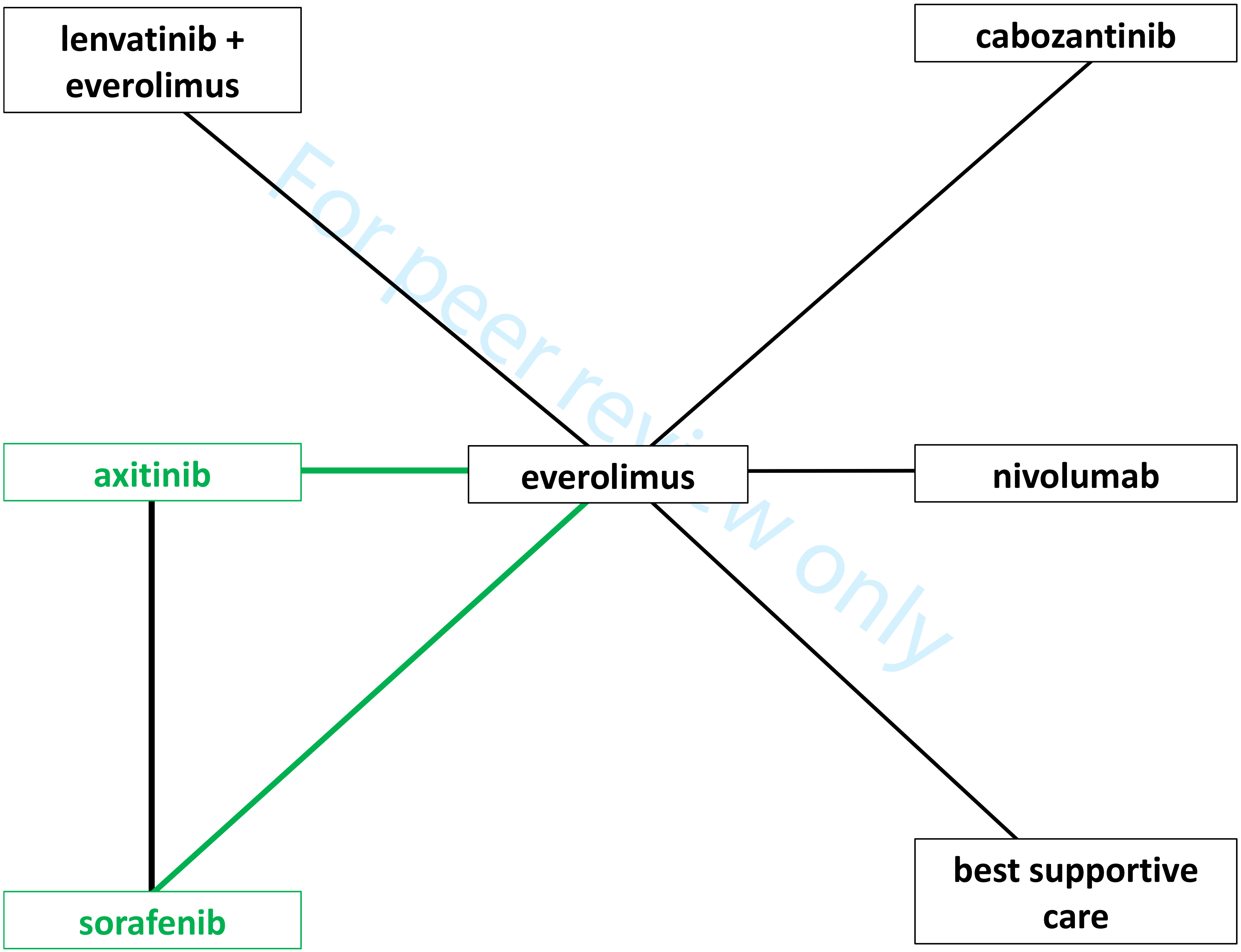

For peer review only - http://bmjopen.bmj.com/site/about/guidelines.xhtml

SUPPLEMENTARY FILE

Targeted therapies for previously treated advanced or metastatic renal cell carcinoma: systematic review and network meta-analysis

Contents

SEARCH STRATEGY 2

DATA EXTRACTION 4

RISK OF BIAS SUMMARIES 7

DATASETS 9

WINBUGS CODE 12

RESULTS 14

REFERENCES 15

For peer review only

For peer review only - http://bmjopen.bmj.com/site/about/guidelines.xhtml

SUPPLEMENTARY FILE

Targeted therapies for previously treated advanced or metastatic renal cell carcinoma: systematic review and network meta-analysis

SEARCH STRATEGY

Table 1. Example search strategy (EMBASE update search for randomised controlled trials)

OVID: EMBASE 1974 to July 03 (searched on 4th July 2017 from Week 3 2016 to Week 27 2017)		
#	Search Terms	Results
1	Carcinoma, Renal Cell/	20712
2	(renal cell carcinoma$ or cell renal carcinoma$ or renal carcinoma$ or kidney carcinoma$ or kidney cell carcinoma$ or renal adenocarcinoma$ or kidney adenocarcinoma$ or adenocarcinoma$renal or adenocarcinoma$kidney$).mp.	66950
3	(hypernephroma$ or nephroid carcinoma$ or hypernephroid carcinoma$ or kidney hypernephroma$ or kidney pelvic carcinoma$ or kidney pyelocarcinoma$ or renal hypernephroma$ or grawitz tumo?r$ or renal cell neoplasm$ or renal cell cancer$ or renal tumo?r$ or carcinoma chromophobe cell kidney$ or chromophobe cell kidney carcinoma$).mp.	17922
4	kidney neoplasms/	10255
5	(cancer$ adj2 kidney$1).ti,ab.	5836
6	(neoplasm$1 adj2 kidney$1).ti,ab.	329
7	(neoplasm$1 adj2 renal).ti,ab.	2153
8	(cancer$ adj2 renal).ti,ab.	12586
9	(tumo?r$1 adj2 kidney$1).ti,ab.	4838
10	(tumo?r$1 adj2 renal).ti,ab.	14674
11	or/1-10	92199
12	(axitinib or inlyta or AG013736 or "AG 013736").mp.	3492
13	(sorafenib or nexavar or bay 43-9006 or bay 439006 or bay43-9006 or bay439006).mp.	23166
14	(sunitinib or sutent or pha 2909040ad or pha2909040ad or "su 010398" or "su 011248" or su 10398 or su10398 or su 11248 or su010398 or su011248 or su11248).mp.	18935
15	(everolimus or afinitor or certican or zortress or nvp-rad-001 or rad-001 or rad 001a or rad001 or rad001a or sdz rad).mp.	23010
16	(nivolumab or opdivo or ONO4538 or ONO 4538 or BMS936558 or BMS 936558 or MDX1106 or MDX 1106).mp.	4666
17	(temsirolimus or cci-779 or cell-cycle-inhibitor-779 or nsc 683864 or nsc683864 or torisel).mp.	7267
18	(bevacizumab or avastin or nsc 704865 or nsc704865 or anti-vegf or rhumab-vegf).mp.	50865
19	(armala or pazopanib or gw786034 or gw 786034 or sb 710468 or sb710468 or votrient).mp.	5612
20	or/12-19	99220
21	Clinical trial/	934498
22	Randomized controlled trial/	460454
23	Randomization/	74486
24	Single blind procedure/	28210
25	Double blind procedure/	140589
26	Crossover procedure/	52369
27	Placebo/	309726
28	Randomi?ed controlled trial$.tw.	162409
29	Rct.tw.	24817
30	Random allocation.tw.	1704
31	Randomly allocated.tw.	28013
32	Allocated randomly.tw.	2271
33	(allocated adj2 random).tw.	867
34	Single blind$.tw.	19692
35	Double blind$.tw.	180274
36	((treble or triple) adj blind$).tw.	721
37	Placebo$.tw.	257991
38	Prospective study/	388181

For peer review only - http://bmjopen.bmj.com/site/about/guidelines.xhtml

SUPPLEMENTARY FILE

Targeted therapies for previously treated advanced or metastatic renal cell carcinoma: systematic review and network meta-analysis

39	or/21-38	1782373
40	Case study/	48285
41	Case report.tw.	342862
42	Abstract report/ or letter/	1025233
43	or/40-42	1408381
44	39 not 43	1736655
45	11 and 20 and 44	3942
46	Animals/ not Humans/	1295518
47	45 not 46	3942
48	(editorial or letter).pt.	1521255
49	47 not 48	3868
50	limit 49 to em=201603-201727	327

For peer review only

For peer review only - http://bmjopen.bmj.com/site/about/guidelines.xhtml

SUPPLEMENTARY FILE

Targeted therapies for previously treated advanced or metastatic renal cell carcinoma: systematic review and network meta-analysis

DATA EXTRACTION

Table 2. Data extraction template

**Study or trial name:**			**Publication source**
**Full reference for all publications:**			
**Design**			
Study design			
Number of centres & Country/countries			
Recruitment dates			
Length of follow-up [include study start date, data cut-off and completion date]			
Source of funding			
Eligibility criteria (inclusion and exclusion)			
**Participants and treatment arms**	**Intervention:**	**Comparator:**	**Publication, data cut-off**
Intervention, method of delivery, dose and frequency			
Concomitant medication(s) or therapies			
Cross-over or post-study interventions allowed<br>Number of patients (%)			
Number of cycles<br>At least one dose reduction n (%)			
Treatment duration (and the data cut offs for each publication for the study)			
Number randomised			
Number who received study medication			
Number withdrawn/ discontinued and reasons<br>[give breakdown]			
Disease stage and/or metastatic disease<br>[give breakdown]			
Previous systemic therapy treatments, n (%) [give breakdown]			
Age, years: median (range)			
Ethnicity, n (%)<br>[give breakdown]			
Male, n (%)			
Performance status n (%)			

For peer review only

For peer review only - http://bmjopen.bmj.com/site/about/guidelines.xhtml

SUPPLEMENTARY FILE

Targeted therapies for previously treated advanced or metastatic renal cell carcinoma: systematic review and network meta-analysis

[give tool and breakdown]			
Reported subgroups	None reported		
**Reported outcomes**			
Primary outcome			
Secondary outcomes			
Outcomes and time points with data reported for subgroups of prior baseline therapies			
Outcomes and time points with data reported for subgroups of baseline prognostic scores (e.g. ECOG, MSKCC)			
**Results**	**Intervention**	**Comparator**	**Publication, data cut-off**
**PFS**			
HR (95% CI)			
HR (95% CI) for subgroups based on prior therapy:			
PFS, median (95% CI) months			
PFS, median (95% CI), months for subgroups based on prior therapy			
Number of progression events n (%)			
**Overall survival**			
HR, (95% CI)			
HR, (95% CI) for subgroups based on prior therapy			
Number of deaths, n (%)			
Median OS, months (95% CI)			
Median OS, (95% CI) months for subgroup based on prior therapy			
Number of deaths, n (%) for subgroups based on prior therapy			
**Response**			
Objective response, n (%)			
Complete response, n (%)			
Partial response, n (%)			
Stable disease, n (%)			
Progressive disease, n (%)			
Time to response, months (median [range])			
Duration of response, median (95% CI), months			
Other measures of response			
**HRQoL**			

For peer review only - http://bmjopen.bmj.com/site/about/guidelines.xhtml

SUPPLEMENTARY FILE

Targeted therapies for previously treated advanced or metastatic renal cell carcinoma: systematic review and network meta-analysis

[Scale 1] Mean end of treatment			
[Scale 1] Mean difference (95% CI)			
Completion rate			
**Adverse events (AE's)**			
N in safety analysis			
Total AE's (any Grade)			
Total AE's Grade ≥3			
[enter list of individual AEs]			

**Risk of bias assessment based (RCTs)**		
**Domain**	**Risk assessment**	**Comments**
Random sequence generation	[Low/High/Unclear]	
Allocation concealment	[Low/High/Unclear]	
Blinding (who [participants, personnel], and method)	[Low/High/Unclear]	
Other biases	[Low/High/Unclear]	
Progression-free survival		
-Blinding of outcome assessment	[Low/High/Unclear]	
-Incomplete outcome data	[Low/High/Unclear]	
-Selective reporting	[Low/High/Unclear]	
Overall survival		
-Blinding of outcome assessment	[Low/High/Unclear]	
-Incomplete outcome data	[Low/High/Unclear]	
-Selective reporting	[Low/High/Unclear]	
Response (partial response, disease stabilisation, progressive disease)		
-Blinding of outcome assessment	[Low/High/Unclear]	
-Incomplete outcome data	[Low/High/Unclear]	
-Selective reporting	[Low/High/Unclear]	
HRQoL		
-Blinding of outcome assessment	[Low/High/Unclear]	
-Incomplete outcome data	[Low/High/Unclear]	
-Selective reporting	[Low/High/Unclear]	
Adverse events		
-Blinding of outcome assessment	[Low/High/Unclear]	
-Incomplete outcome data	[Low/High/Unclear]	
-Selective reporting	[Low/High/Unclear]	

For peer review only - http://bmjopen.bmj.com/site/about/guidelines.xhtml

SUPPLEMENTARY FILE

Targeted therapies for previously treated advanced or metastatic renal cell carcinoma: systematic review and network meta-analysis

RISK OF BIAS SUMMARIES

Table 3. Summary of Cochrane risk of bias assessment for randomised control trials

**Criteria**		**AXIS**	**Checkmate -025**	**HOPE 205**	**METEOR**	**RECORD-1**
All outcomes	Random sequence generation	✓	✓	✓	✓	✓
	Allocation concealment	✓	✓	✓	✓	✓
	Blinding: participant/personnel	x	X	X	X	✓
**Outcome-specific**						
OS	Blinding: outcome assessment	✓	✓	✓	✓	✓
	Incomplete outcome data	✓	?	✓	?	✓
	Selective Reporting	✓	✓	✓	✓	✓
	Other Biases	?	?	?	?	?
PFS	Blinding: outcome assessment	✓	X	✓	✓	✓
	Incomplete outcome data	✓	✓	✓	?	✓
	Selective Reporting	✓	✓	✓	✓	✓
	Other Biases	**NA**	**NA**	?	**NA**	**NA**
ORR	Blinding: outcome assessment	✓	X	✓	✓	✓
	Incomplete outcome data	?	✓	✓	?	✓
	Selective Reporting	✓	✓	✓	✓	?
	Other Biases	**NA**	**NA**	?	**NA**	**NA**
HRQoL	Blinding: outcome assessment	x	X	**NA**	X	x
	Incomplete outcome data	✓	✓	**NA**	?	✓
	Selective Reporting	✓	✓	**NA**	✓	x
	Other Biases	**NA**	**NA**	**NA**	**NA**	**NA**
AE	Blinding: outcome assessment	x	X	X	X	✓
	Incomplete outcome data	✓	✓	✓	✓	✓
	Selective Reporting	✓	✓	✓	✓	✓
	Other Biases	**NA**	**NA**	?	**NA**	**NA**

Key: ✓, low risk; ?, unclear risk; X, high risk; NA, not applicable.
Abbreviations: OS, overall survival; PFS, progression-free survival; ORR, objective response rate; HRQoL, health-related quality of life; AE, adverse effects.

For peer review only - http://bmjopen.bmj.com/site/about/guidelines.xhtml

SUPPLEMENTARY FILE

Targeted therapies for previously treated advanced or metastatic renal cell carcinoma: systematic review and network meta-analysis

Table 4. Summary of ROBINS-I risk of bias assessments in non-randomised studies

	Guida 2017	Heng 2016	Iacovelli 2015	Lakomy 2017	SPAZO-2	Vogelzang 2016	Wong 2014
**Overall survival**							
Confounding	~	~	x	x	~	~	~
Selection	✓	✓	✓	✓	✓	✓	✓
Intervention classification	✓	✓	✓	✓	✓	✓	✓
Intervention deviations	✓	✓	✓	✓	✓	✓	✓
Missing data	✓	NI	✓	✓	✓	✓	x
Outcome measures	✓	✓	✓	✓	✓	✓	✓
Outcome reporting	✓	✓	✓	✓	✓	✓	✓
**Overall judgement**	x	~	x	x	~	~	x
**Progression-free survival**							
Confounding	x	~	-	x	~	~	~
Selection	✓	✓	-	✓	✓	✓	✓
Intervention classification	✓	✓	-	✓	✓	✓	✓
Intervention deviations	✓	✓	-	✓	✓	✓	✓
Missing data	✓	NI	-	✓	✓	✓	x
Outcome measures	x	x	-	x	x	x	x
Outcome reporting	x	x	-	x	x	x	x
**Overall judgement**	x	x	-	x	x	x	x

Abbreviations: PFS = progression-free survival; OS = overall survival

Key: ✓, low risk; ~, moderate risk; x, serious risk; x, critical risk; NI, no information.

For peer review only - http://bmjopen.bmj.com/site/about/guidelines.xhtml

SUPPLEMENTARY FILE

Targeted therapies for previously treated advanced or metastatic renal cell carcinoma: systematic review and network meta-analysis

DATASETS

Table 5. Study data: overall survival

Study	Data details	T1	T2	HR (95% CI)	T1 median months (95% CI)	T2 median (95% CI)
**AXIS[1]**	**Prior sunitinib subset**	**5**	**6**	**1.00 (0.78 to 1.27)**	**NR**	**NR**
**CheckMate 025[2]**	**-**	**3**	**1**	**0.73 (0.57 to 0.93)**	**25.0 (21.8 to NE)**	**19.6 (17.6 to 23.1)**
**HOPE 205[3]**	**July 2015 cutoff**	**7**	**1**	**0.59 (0.36 to 0.97)**	**25.5 (16.4 to 32.1)**	**15.4 (11.8 to 20.6)**
**METEOR[4]**	**31 Dec 2015 cutoff**	**2**	**1**	**0.66 (0.53 to 0.83)**	**21.4 (18.7 to NE)**	**16.5 (14.7 to 18.8)**
**RECORD-1[5]**	**RPSFT adjusted**	**1**	**4**	**0.60 (0.22 to 1.65)**	**14.4 (NR)**	**10.0 (NR)**
Guida 2017[6]*		5	1	1.33 (0.8 to 2.1)	14.9 (7.4 to 22.4)	21.5 (16.5 to 26.5)
Heng 2016[7]	-	6	1	1.25 (0.75 to 2.10)	18.7 (NR)	23.0 (NR)
Heng 2016[7]	-	5	1	1.22 (0.77 to 1.94)	23.5 (NR)	23.0 (NR)
Iacovelli 2015[8]	-	6	1	2.21 (1.47 to 3.31)	NR	NR
SPAZO-2[9]	Adjusted results	5	1	0.81 (0.60 to 1.20)	11.6 (7 to 16)	9.5 (7 to 12)
Vogelzang 2016[10]	-	1	5	1.16 (0.74 to 1.82)	NR	NR
Wong 2014[11]	Full adjusted results	1	6	0.66 (0.44 to 0.99)	19.0 (NR)	13.8 (NR)

Abbreviations: T1, treatment 1; T2, treatment 2 (baseline); HR, hazard ratio; CI, confidence interval; NE, not estimable; RPSFT, rank preserving structural failure time model; NR, not reported.
Treatment codes: 1, everolimus; 2, cabozantinib; 3, nivolumab; 4, best supportive care/placebo; 5, axitinib; 6, sorafenib; 7, lenvatinib with everolimus. Studies in bold formed the primary analysis.
*Data from personal communication with the study author, 11 March 2018

Table 6. Study data: progression-free survival

Study	Data details	T1	T2	HR (95% CI)	T1 median months (95% CI)	T2 median (95% CI)
**HOPE 205[12]**	**IRR**	**7**	**1**	**0.45 (0.27 to 0.79)**	**12.8 (7.4 to 17.5)**	**5.6 (3.6 to 9.3)**
**METEOR[4]**	**IRR for ITT**	**2**	**1**	**0.51 (0.41 to 0.62)**	**7.4 (6.6 to 9.1)**	**3.9 (3.7 to 5.1)**
**RECORD-1[13]**	**Final analysis, ICR**	**1**	**4**	**0.33 (0.25 to 0.43)**	**4.9 (4.0 to 5.5)**	**1.9 (1.8 to 1.9)**
AXIS[1]	Prior sunitinib subset	5	6	0.72 (0.57 to 0.90)	6.5 (5.7 to 7.9)	4.4 (2.9 to 4.7)
Guida 2017[6]*		5	1	0.84 (0.55 to 1.2)	7.7 (5.3 to 10.2)	5.3 (4.0 to 6.6)
Heng 2016[7]	-	6	1	1.47 (0.95 to 2.28)	-	-
Heng 2016[7]	-	5	1	1.26 (0.81 to 1.95)	-	-
Iacovelli 2015[8]	-	6	1	NR	NR	NR
SPAZO-2[9]	Adjusted results	5	1	0.76 (0.5 to 1.1)	5.3 (3 to 7)	4.6 (3 to 6)
Vogelzang	Adjusted results	1	5	1.16 (0.85 to 1.59)	NR	NR

For peer review only - http://bmjopen.bmj.com/site/about/guidelines.xhtml

SUPPLEMENTARY FILE

Targeted therapies for previously treated advanced or metastatic renal cell carcinoma: systematic review and network meta-analysis

2016[10]						
Wong 2014[11]	Adjusted results	1	6	0.76 (0.55 to 1.04)	10.1 (NR)	8.6 (NR)

Abbreviations: T1, treatment 1; T2, treatment 2 (baseline); HR, hazard ratio; CI, confidence interval; NR, not reported.
Treatment codes: 1, everolimus; 2, cabozantinib; 3, nivolumab; 4, best supportive care/placebo; 5, axitinib; 6, sorafenib; 7, lenvatinib with everolimus. Studies in bold formed the primary analysis.
*Data from personal communication with the study author, 11 March 2018

Table 7. Study data: objective response rate

			**N**		**Objective response**	
**Study**	**T1**	**T2**	**T1**	**T2**	**T1**	**T2**
AXIS[14]	5	6	361	362	70	34
CheckMate 025[2]	3	1	410	411	103	22
HOPE 205[12]	7	1	51	50	18	0
METEOR[4]	2	1	330	328	57	11
RECORD-1[13]	1	4	277	139	5	0

Abbreviations: T1, treatment 1; T2, treatment 2 (baseline).
Treatment codes: 1, everolimus; 2, cabozantinib; 3, nivolumab; 4, best supportive care/placebo; 5, axitinib; 6, sorafenib; 7, lenvatinib with everolimus

Table 8. Study data: grade 3 or 4 adverse events

			**N**		**Grade 3 or 4 adverse events**	
**Study**	**T1**	**T2**	**T1**	**T2**	**T1**	**T2**
AXIS[1]	5	6	359	355	NR	NR
CheckMate 025[2]	3	1	406	397	76	145
HOPE 205[15]	7	1	51	50	36	25
METEOR[4]	2	1	331	322	235	193
RECORD-1[13]	1	4	274	137	NR	NR

Abbreviations: T1, treatment 1; T2, treatment 2 (baseline); HR, hazard ration; CI, confidence interval; NE, not estimable; RPSFT, rank preserving structural failure time model; NR, not reported.
Treatment codes: 1, everolimus; 2, cabozantinib; 3, nivolumab; 4, best supportive care/placebo; 5, axitinib; 6, sorafenib; 7, lenvatinib with everolimus

Table 9. Study data: health-related quality of life (not meta-analysed)

**Study**	**T1**	**T2**	**Study analysis details**	**FKSI scales**	**EuroQol scales**	**EORTC QLQ-C30**
AXIS[16]	5	6	End of treatment MD (95% CI)	DRS: MD 0.12 (-0.45 to 0.69) p = 0.68;<br>FKSI-15: MD 0.35 (-0.63 to 1.34) p = 0.48	5D Index: MD 0.02 (-0.01 to 0.05) p = 0.19<br>VAS: -0.53 (-2.77 to 1.72) p = 0.65	NR
CheckMate	3	1	Median change	DRS:	NR	NR

For peer review only - http://bmjopen.bmj.com/site/about/guidelines.xhtml

SUPPLEMENTARY FILE

Targeted therapies for previously treated advanced or metastatic renal cell carcinoma: systematic review and network meta-analysis

025[2]			(range) at week 104	niv -2 (-1 to 16)<br>evo 2 (-7 to 15)		
HOPE 205	7	1	NA	NR	NR	NR
METEOR	2	1	NA	NR	NR	NR
RECORD-1[17]	1	4	Time to deterioration HR (95% CI); results favour placebo	DRS: HR 0.82, 95% CI 0.75 to 0.92, p = 0.001	NR	Global health status HR 0.85 (0.75 to 0.96) p = 0.006<br>Physical functioning HR 0.84 (0.75 to 0.94) p = 0.001

Abbreviations: FKSI-DRS = Functional Assessment of Cancer Therapy (FACT) Kidney Cancer Symptom Index; DRS = Disease-related Symptoms subscale of the FKSI-15; EQ-5D = European Quality of Life self-report questionnaire; VAS = visual analogue scale; EORTC QLQ = European Organization for the Research and Treatment of Cancer Quality of Life Questionnaire; MD, mean difference; CI, confidence interval; HR, hazard ratio; NA, not applicable; NR, not reported

For peer review only

SUPPLEMENTARY FILE

Targeted therapies for previously treated advanced or metastatic renal cell carcinoma: systematic review and network meta-analysis

WINBUGS CODE

CODE 1: Fixed effect log hazard ratio NMA for 2-arm studies (overall survival primary and progression-free survival primary)

```
model{
#Model for log-hazard ratios
for(i in 1:ndp){
        prec[i]<- 1/(se[i]*se[i])
                lhr[i]~dnorm(md[i],prec[i])

#Fixed effect model for log hazard ratios
                md[i] <- d[t[i]] - d[b[i]]

#Deviance residuals for data i
  dev[i] <- (lhr[i] - md[i])*(lhr[i] - md[i])/(se[i]*se[i])
        }
        resdev <- sum(dev[])

#Give priors for log hazard ratios
        d[1]<-0
        for (k in 2:nt){
        d[k] ~ dnorm(0,.001)
        }

#Rank the treatment effects (with 1=best) & record the best treatment
for(k in 1:nt){
        rk[k]<- rank(d[],k)
        best[k]<-equals(rk[k],1)
        }

#All pairwise log hazard ratios and hazard ratios
for (c in 1:nt-1){
        for (k in (c+1):nt){
                lhzr[c,k] <- d[k] - d[c]
                HR[c,k] <- exp(lhzr[c,k])
                }
        }
}
```

CODE 2: Fixed effect odds ratio NMA (objective response rate analysis)

```
model{

for(i in 1:ns){

        delta[i,t[i,1]]<-0
        mu[i] ~ dnorm(0,.0001)

        for (k in 1:na[i]) {
                r[i,t[i,k]] ~ dbin(p[i,t[i,k]],n[i,t[i,k]])
```


For peer review only - http://bmjopen.bmj.com/site/about/guidelines.xhtml

SUPPLEMENTARY FILE

Targeted therapies for previously treated advanced or metastatic renal cell carcinoma: systematic review and network meta-analysis

```
				logit(p[i,t[i,k]])<-mu[i] + delta[i,t[i,k]]

		rhat[i,t[i,k]]<- p[i,t[i,k]] * n[i,t[i,k]]

		resdev[i,k]<- 2 * (r[i,t[i,k]] * (log(r[i,t[i,k]]) - log(rhat[i,t[i,k]])) + (n[i,t[i,k]] - r[i,t[i,k]]) * (log(n[i,t[i,k]] - r[i,t[i,k]]) -
log(n[i,t[i,k]] - rhat[i,t[i,k]])))
		}
		sumdev[i]<-sum(resdev[i,1:na[i]])

for (k in 2:na[i]) {
		delta[i,t[i,k]] <- d[t[i,k]] - d[t[i,1]]		# trial-specific LOR
		}
}

sumdevtot<- sum(sumdev[])

d[1]<-0
for (k in 2:nt){
		d[k] ~ dnorm(0,.0001)
		}

for (i in 1:ns) {
		mu1[i] <- mu[i] * equals(t[i,1],1)
		}

for (k in 1:nt) {
		logit(T[k])<- sum(mu1[])/nb +d[k]
		}

for (k in 1:nt) {
		rk[k]<-nt - rank(T[],k)
		best[k]<-equals(rk[k],1)
		}

for (c in 1:(nt-1)) {
		for (k in (c+1):nt) {
				lor[c,k] <- (d[k] - d[c])
				or[c,k]<-exp(lor[c,k])
						}
				}
}
```

CODE 3: Fixed effect log hazard ratio NMA to combine 2-arm and multi-arm studies (overall survival sensitivity and progression-free survival sensitivity)

```
model{

# Priors

   #On tx effect mean
   beta[1] < -0
   for (tt in 2:nt){
   beta[tt]~dnorm(0,1.0E-6)
   }

   #On individual study baseline effect
```

SUPPLEMENTARY FILE

Targeted therapies for previously treated advanced or metastatic renal cell carcinoma: systematic review and network meta-analysis

```
    for(ss in 1:ns){
    alpha[ss] ~ dnorm(0,1.0E-6)
    }

# Fit data
    for(ii in 1:ndp){
    mu[ii] < - alpha[t[ii]]*multi[ii] + beta[tx[ii]] - beta[b[ii]]
    prec[ii] < - 1/pow(se[ii],2)
    m[ii] ~ dnorm(mu[ii],prec[ii])
    }

# Calculate HRs
    for (hh in 1:nt) {
    hr[hh] < -exp(beta[hh])
    }

# Rank
    for (ll in 1:nt) {
                    rk[ll]<-rank(beta[],ll)
      best[ll] < - equals(rk[ll],1)
       }
    }
}
```

RESULTS

Table 10. Results of the RCT network meta-analyses for objective response rate, with 0.5 correction of 0 values

	Best supportive care	Lenvatinib+ everolimus	Nivolumab	Cabozantinib	Everolimus
Everolimus	0.24 (0.00 to 1.39)	91190 (9.30 to 34400)	6.23 (3.78 to 10.01)	6.61 (3.27 to 12.55)	-
Cabozantinib	0.04 (0.00 to 0.24)	14500 (1.36 to 5629)	1.06 (0.41 to 2.19)	-	0.15 (0.08 to 0.31)
Nivolumab	0.00 (0.00 to 0.24)	7.73 (1.46 to 5652)	-	0.94 (0.46 to 2.41)	0.16 (0.10 to 0.26)
Lenvatinib + everolimus	0.01 (0.00 to 0.04)	-	0.13 (0.00 to 0.68)	0.00 (0.00 to 0.74)	0.00 (0.00 to 0.11)
BSC	-	193 (24.99 to 4494382)	657000 (4.15 to 9580)	23.53 (4.10 to 30312)	4.02 (0.72 to 4826)

Abbreviations: BSC, best supportive care; OS, overall survival; PFS, progression-free survival
Results are odds ratios with 95% credible interval; odds ratios > 1 favour the treatment along the top row.

For peer review only - http://bmjopen.bmj.com/site/about/guidelines.xhtml

SUPPLEMENTARY FILE

Targeted therapies for previously treated advanced or metastatic renal cell carcinoma: systematic review and network meta-analysis

REFERENCES

1. Motzer RJ, Escudier B, Tomczak P, Hutson TE, Michaelson MD, Negrier S, et al. Axitinib versus sorafenib as second-line treatment for advanced renal cell carcinoma: overall survival analysis and updated results from a randomised phase 3 trial, 2013. Available from: http://onlinelibrary.wiley.com/o/cochrane/clcentral/articles/055/CN-00864055/frame.html. Date accessed.
2. Motzer RJ, Escudier B, McDermott DF, George S, Hammers HJ, Srinivas S, et al. Nivolumab versus Everolimus in Advanced Renal-Cell Carcinoma, 2015. Available from: http://onlinelibrary.wiley.com/o/cochrane/clcentral/articles/537/CN-01108537/frame.html. Date accessed.
3. Hutson TE, Dutcus CE, Ren M, Baig MA, Fishman MN. Subgroup analyses and updated overall survival from the phase II trial of lenvatinib (LEN), everolimus (EVE), and LEN+EVE in metastatic renal cell carcinoma (mRCC). Journal of Clinical Oncology Conference 2016; 34.
4. Choueiri T, Escudier B, Powles T, Tannir N, Mainwaring P, Rin iB. Cabozantinib versus everolimus in advanced renal cell carcinoma (METEOR): final results from a randomised, open-label, phase 3 trial. The Lancet Oncology 2016; 17: 917-27.
5. Korhonen P, Zuber E, Branson M, al. e. Correcting overall survival for the impact of crossover via a rank-preserving structural failure time (RPSFT) model in the RECORD-1 trial of everolimus in metastatic renal-cell carcinoma. J Biopharm Stat 2012; 22: 1258-71.
6. Guida A, Albiges L, Derosa L, Loriot Y, Massard C, Fizazi K, et al. Everolimus Versus Axitinib as Second-line Therapy in Metastatic Renal Cell Carcinoma: Experience From Institut Gustave Roussy. Clinical Genitourinary Cancer 2017; 15: e1081-e8.
7. Heng DY, Signorovitch J, Swallow E, Li N, Zhong Y, Qin P, et al. Comparative Effectiveness of Second-Line Targeted Therapies for Metastatic Renal Cell Carcinoma: A Systematic Review and Meta-Analysis of Real-World Observational Studies. PLoS ONE [Electronic Resource] 2014; 9: e114264.
8. Iacovelli R, Santini D, Rizzo M, Felici A, Santoni M, Verzoni E, et al. Bone metastases affect prognosis but not effectiveness of third-line targeted therapies in patients with metastatic renal cell carcinoma. Can Urol Assoc J 2015; 9: 263-7.
9. Arranz Arija J, Perez-Valderrama B, Rodriguez Sanchez A, Puertas Alvarez J, Pinto Marin A, Maximiano Alonso C, et al. SPAZO2 (SOGUG): Comparative effectiveness of everolimus (Ev) vs axitinib (Ax) as second-line after first-line pazopanib (1stPz) in metastatic renal carcinoma (mRC). Annals of Oncology 2017; 28 (Supplement 5): v313.
10. Vogelzang NJ, Pal SK, Signorovitch JE, Reichmann WM, Li N, Yang C, et al. Comparative effectiveness of everolimus and axitinib as second targeted therapies for metastatic renal cell carcinoma in the US: A retrospective chart review. Current Medical Research and Opinion 2016; 32: 741-7.
11. Wong MK, Yang H, Signorovitch JE, Wang X, Liu Z, Liu NS, et al. Comparative outcomes of everolimus, temsirolimus and sorafenib as second targeted therapies for metastatic renal cell carcinoma: A US medical record review. Current Medical Research and Opinion 2014; 30: 537-45.
12. Motzer RJ, Hutson TE, Ren M, Dutcus C, Larkin J. Independent assessment of lenvatinib plus everolimus in patients with metastatic renal cell carcinoma, 2016. Available from: http://onlinelibrary.wiley.com/o/cochrane/clcentral/articles/453/CN-01168453/frame.html. Date accessed.
13. Motzer RJ, Escudier B, Oudard S, Hutson TE, Porta C, Bracarda S, et al. Phase 3 trial of everolimus for metastatic renal cell carcinoma : final results and analysis of prognostic factors, 2010. Available from: http://onlinelibrary.wiley.com/o/cochrane/clcentral/articles/159/CN-00762159/frame.html. Date accessed.
14. Rini BI, Escudier B, Tomczak P, Kaprin A, Szczylik C, Hutson TE, et al. Comparative effectiveness of axitinib versus sorafenib in advanced renal cell carcinoma (AXIS): a randomised phase 3 trial, 2011. Available from: http://onlinelibrary.wiley.com/o/cochrane/clcentral/articles/402/CN-00804402/frame.html. Date accessed.
15. Motzer RJ, Hutson TE, Glen H, Michaelson MD, Molina A, Eisen T, et al. Lenvatinib, everolimus, and the combination in patients with metastatic renal cell carcinoma: a randomised, phase 2,open-label, multicentre trial. Lancet Oncology 2015; 16: 1473-82.

For peer review only - http://bmjopen.bmj.com/site/about/guidelines.xhtml

SUPPLEMENTARY FILE

Targeted therapies for previously treated advanced or metastatic renal cell carcinoma: systematic review and network meta-analysis

16. Cella D, Escudier B, Rini B, Chen C, Bhattacharyya H, Tarazi J, et al. Patient-reported outcomes for axitinib vs sorafenib in metastatic renal cell carcinoma: phase III (AXIS) trial. British Journal of Cancer 2013; 108: 1571-8.
17. Beaumont JL, Butt Z, Baladi J, Motzer RJ, Haas T, Hollaender N, et al. Patient-reported outcomes in a phase iii study of everolimus versus placebo in patients with metastatic carcinoma of the kidney that has progressed on vascular endothelial growth factor receptor tyrosine kinase inhibitor therapy, 2011. Available from: http://onlinelibrary.wiley.com/o/cochrane/clcentral/articles/925/CN-00800925/frame.html

For peer review only

For peer review only - http://bmjopen.bmj.com/site/about/guidelines.xhtml

PRISMA 2009 Checklist

Section/topic	#	Checklist item	Reported on page #
**TITLE**			
Title	1	Identify the report as a systematic review, meta-analysis, or both.	1
**ABSTRACT**			
Structured summary	2	Provide a structured summary including, as applicable: background; objectives; data sources; study eligibility criteria, participants, and interventions; study appraisal and synthesis methods; results; limitations; conclusions and implications of key findings; systematic review registration number.	2-4
**INTRODUCTION**			
Rationale	3	Describe the rationale for the review in the context of what is already known.	7
Objectives	4	Provide an explicit statement of questions being addressed with reference to participants, interventions, comparisons, outcomes, and study design (PICOS).	7
**METHODS**			
Protocol and registration	5	Indicate if a review protocol exists, if and where it can be accessed (e.g., Web address), and, if available, provide registration information including registration number.	3
Eligibility criteria	6	Specify study characteristics (e.g., PICOS, length of follow-up) and report characteristics (e.g., years considered, language, publication status) used as criteria for eligibility, giving rationale.	7-9
Information sources	7	Describe all information sources (e.g., databases with dates of coverage, contact with study authors to identify additional studies) in the search and date last searched.	9-10
Search	8	Present full electronic search strategy for at least one database, including any limits used, such that it could be repeated.	Suppl. 2-3, Table 1
Study selection	9	State the process for selecting studies (i.e., screening, eligibility, included in systematic review, and, if applicable, included in the meta-analysis).	9, 11
Data collection process	10	Describe method of data extraction from reports (e.g., piloted forms, independently, in duplicate) and any processes for obtaining and confirming data from investigators.	9-10
Data items	11	List and define all variables for which data were sought (e.g., PICOS, funding sources) and any assumptions and simplifications made.	9-10, suppl. 4-6, Table 2
Risk of bias in individual studies	12	Describe methods used for assessing risk of bias of individual studies (including specification of whether this was done at the study or outcome level), and how this information is to be used in any data synthesis.	10
Summary measures	13	State the principal summary measures (e.g., risk ratio, difference in means).	11
Synthesis of results	14	Describe the methods of handling data and combining results of studies, if done, including measures of consistency (e.g., I^2) for each meta-analysis.	10-11

For peer review only - http://bmjopen.bmj.com/site/about/guidelines.xhtml

PRISMA 2009 Checklist

Section/topic	#	Checklist item	Reported on page #
Risk of bias across studies	15	Specify any assessment of risk of bias that may affect the cumulative evidence (e.g., publication bias, selective reporting within studies).	10
Additional analyses	16	Describe methods of additional analyses (e.g., sensitivity or subgroup analyses, meta-regression), if done, indicating which were pre-specified.	11
**RESULTS**			
Study selection	17	Give numbers of studies screened, assessed for eligibility, and included in the review, with reasons for exclusions at each stage, ideally with a flow diagram.	Fig. 1
Study characteristics	18	For each study, present characteristics for which data were extracted (e.g., study size, PICOS, follow-up period) and provide the citations.	Table 1
Risk of bias within studies	19	Present data on risk of bias of each study and, if available, any outcome level assessment (see item 12).	16-17, suppl. 7-8, Table 3-4
Results of individual studies	20	For all outcomes considered (benefits or harms), present, for each study: (a) simple summary data for each intervention group (b) effect estimates and confidence intervals, ideally with a forest plot.	Suppl. 9-11, Table 5-9
Synthesis of results	21	Present results of each meta-analysis done, including confidence intervals and measures of consistency.	17-19, Table 2, Suppl. 14, Table 10
Risk of bias across studies	22	Present results of any assessment of risk of bias across studies (see Item 15).	4, 16-18
Additional analysis	23	Give results of additional analyses, if done (e.g., sensitivity or subgroup analyses, meta-regression [see Item 16]).	17-18, Table 2
**DISCUSSION**			
Summary of evidence	24	Summarize the main findings including the strength of evidence for each main outcome; consider their relevance to key groups (e.g., healthcare providers, users, and policy makers).	20, 23
Limitations	25	Discuss limitations at study and outcome level (e.g., risk of bias), and at review-level (e.g., incomplete retrieval of identified research, reporting bias).	21-22
Conclusions	26	Provide a general interpretation of the results in the context of other evidence, and implications for future research.	22-23
**FUNDING**			
Funding	27	Describe sources of funding for the systematic review and other support (e.g., supply of data); role of funders for the systematic review.	25

For peer review only - http://bmjopen.bmj.com/site/about/guidelines.xhtml

PRISMA 2009 Checklist

From: Moher D, Liberati A, Tetzlaff J, Altman DG, The PRISMA Group (2009). Preferred Reporting Items for Systematic Reviews and Meta-Analyses: The PRISMA Statement. PLoS Med 6(7): e1000097. doi:10.1371/journal.pmed1000097

For more information, visit: **www.prisma-statement.org**.

For peer review only

Reporting checklist for meta-analysis of observational studies.

Based on the MOOSE guidelines.

Instructions to authors

Complete this checklist by entering the page numbers from your manuscript where readers will find each of the items listed below.

Your article may not currently address all the items on the checklist. Please modify your text to include the missing information. If you are certain that an item does not apply, please write "n/a" and provide a short explanation.

Upload your completed checklist as an extra file when you submit to a journal.

In your methods section, say that you used the MOOSE reporting guidelines, and cite them as:

Stroup DF, Berlin JA, Morton SC, Olkin I, Williamson GD, Rennie D, Moher D, Becker BJ, Sipe TA, Thacker SB. Meta-analysis of observational studies in epidemiology: a proposal for reporting. Meta-analysis Of Observational Studies in Epidemiology (MOOSE) group. JAMA. 2000; 283(15):2008-2012.

	Reporting Item	Page Number
#1	Identify the study as a meta-analysis of observational research	2
#2	Provide a structured summary including, as applicable: background; objectives; data sources; study eligibility criteria, participants, and interventions; study appraisal and synthesis methods; results; limitations; conclusions and implications of key findings; systematic review registration number (From PRISMA checklist)	2-4
#3a	Problem definition	7
#3b	Hypothesis statement	n/a
#3c	Description of study outcomes	8
#3d	Type of exposure or intervention used	8
#3e	Type of study designs used	8

For peer review only

	#3f	Study population	8
Search strategy	#4a	Qualifications of searchers (eg, librarians and investigators)	24
	#4b	Search strategy, including time period included in the synthesis and keywords	9-10, Suppl. 2-3, Table 1
	#4c	Effort to include all available studies, including contact with authors	10
	#4d	Databases and registries searched	9
	#4e	Search software used, name and version, including special features used (eg, explosion)	Suppl. 2, Table 1
	#4f	Use of hand searching (eg, reference lists of obtained articles)	9
	#4g	List of citations located and those excluded, including justification	n/a, word count restriction
	#4h	Method of addressing articles published in languages other than English	9
	#4i	Method of handling abstracts and unpublished studies	9
	#4j	Description of any contact with authors	10
	#5a	Description of relevance or appropriateness of studies gathered for assessing the hypothesis to be tested	12-17
	#5b	Rationale for the selection and coding of data (eg, sound clinical principles or convenience)	9-10
	#5c	Documentation of how data were classified and coded (eg, multiple raters, blinding, and interrater reliability)	9-10
	#5d	Assessment of confounding (eg, comparability of cases and controls in studies where appropriate)	10
	#5e	Assessment of study quality, including blinding of quality assessors; stratification or regression on possible predictors of study results	10
	#5f	Assessment of heterogeneity	10
	#5g	Description of statistical methods (eg, complete description of fixed or random effects models, justification of whether the chosen	10-11

For peer review only

	models account for predictors of study results, dose-response models, or cumulative meta-analysis) in sufficient detail to be replicated	
#5h	Provision of appropriate tables and graphics	Figure 1,2, p13-14, Table 1, p18 Table 2, Suppl. all Tables
#6a	Graphic summarizing individual study estimates and overall estimate	18, Table 2, suppl. 9-11, Table 5-9
#6b	Table giving descriptive information for each study included	13-14, Table 1
#6c	Results of sensitivity testing (eg, subgroup analysis)	17-18, Table 2
#6d	Indication of statistical uncertainty of findings	18, Table 2
#7a	Quantitative assessment of bias (eg. publication bias)	10, 17-18
#7b	Justification for exclusion (eg, exclusion of non–English-language citations)	9
#7c	Assessment of quality of included studies	16-17, suppl. 7-8, Table 3-4
#8a	Consideration of alternative explanations for observed results	21
#8b	Generalization of the conclusions (ie, appropriate for the data presented and within the domain of the literature review)	22-23
#8c	Guidelines for future research	23
#8d	Disclosure of funding source	25

Reproduced with permission from JAMA. 2000. 283(15):2008-2012. Copyright © 2000 American Medical Association. All rights reserved.This checklist can be completed online using https://www.goodreports.org/, a tool made by the EQUATOR Network in collaboration with Penelope.ai

For peer review only

BMJ Open

TARGETED THERAPIES FOR PREVIOUSLY TREATED ADVANCED OR METASTATIC RENAL CELL CARCINOMA: SYSTEMATIC REVIEW AND NETWORK META-ANALYSIS

Journal:	*BMJ Open*
Manuscript ID	bmjopen-2018-024691.R1
Article Type:	Research
Date Submitted by the Author:	28-Nov-2018
Complete List of Authors:	Karner, Charlotta; British Medical Journal Technology Assessment Group (BMJ-TAG) BMA House Tavistock Square London, UK WC1H 9JR<br>Kew, Kayleigh; British Medical Journal Technology Assessment Group (BMJ-TAG) BMA House Tavistock Square London, UK WC1H 9JR<br>Wakefield, Victoria; British Medical Journal Technology Assessment Group (BMJ-TAG) BMA House Tavistock Square London, UK WC1H 9JR<br>Masento, Natalie; British Medical Journal Technology Assessment Group (BMJ-TAG) BMA House Tavistock Square London, UK WC1H 9JR<br>Edwards, S; British Medical Journal Technology Assessment Group (BMJ-TAG) BMA House Tavistock Square London, UK WC1H 9JR
**Primary Subject Heading**:	Oncology
Secondary Subject Heading:	Renal medicine, Pharmacology and therapeutics, Evidence based practice, Oncology
Keywords:	metastatic renal cell carcinoma, network meta-analysis, overall survival, progression-free survival, systematic reveiw

SCHOLARONE™
Manuscripts

TARGETED THERAPIES FOR PREVIOUSLY TREATED ADVANCED OR METASTATIC RENAL CELL CARCINOMA: SYSTEMATIC REVIEW AND NETWORK META-ANALYSIS

Charlotta Karner PhD, Kayleigh Kew MA (Cantab), Victoria Wakefield MBChB, Natalie Masento PhD, Steven J. Edwards DPhil*

Affiliations: BMJ Technology Assessment Group (BMJ-TAG), BMA House, Tavistock Square, London, WC1H 9JP, UK (all authors)

*corresponding author: sedwards@bmj.com

Word count: 4227 (excluding title page, abstract, article summary, references, figures and tables)

Topic area: Oncology

Key words: metastatic renal cell carcinoma, network meta-analysis, overall survival, progression-free survival, systematic review

Checklists completed: PRISMA 2009, MOOSE

For peer review only - http://bmjopen.bmj.com/site/about/guidelines.xhtml

ABSTRACT

Objective

To compare the effectiveness and safety of treatments for advanced or metastatic renal cell carcinoma (amRCC) after treatment with vascular endothelial growth factor (VEGF)-targeted treatment.

Design

Systematic review and network meta-analysis of randomised controlled trials (RCTs) and comparative observational studies. MEDLINE, EMBASE, and The Cochrane Library were searched up to January 2018. Data extraction and critical appraisals were conducted in duplicate.

Participants

People with amRCC requiring treatment after VEGF-targeted treatment.

Interventions

Axitinib, cabozantinib, everolimus, lenvatinib with everolimus, nivolumab, sorafenib and best supportive care (BSC).

Outcomes

Primary outcomes were overall survival (OS) and progression-free survival (PFS); secondary outcomes were objective response rate (ORR), adverse events, and health-related quality of life (HRQoL).

Results

Twelve studies were included (n = 5,144): five RCTs and seven observational studies. Lenvatinib with everolimus significantly increased OS and PFS over everolimus (HR 0.61, 95%

For peer review only
For peer review only - http://bmjopen.bmj.com/site/about/guidelines.xhtml

Credible Interval [95%CrI]: 0.36 to 0.96 and 0.47, 95%CrI: 0.26 to 0.77, respectively) as did cabozantinib (HR 0.66, 95%CrI: 0.53 to 0.82 and 0.51, 95%CrI: 0.41 to 0.63, respectively). This remained the case when observational evidence was included. Nivolumab also significantly improved OS versus everolimus (HR 0.74, 95%CrI: 0.57 to 0.93). OS sensitivity analysis, including observational studies, indicates everolimus being more effective than axitinib followed by sorafenib. However, inconsistency was identified in the OS sensitivity analysis. PFS sensitivity analysis suggests axitinib is more effective than everolimus, which is more effective than sorafenib. The results for ORR supported the primary OS and PFS analyses. Nivolumab is associated with fewer grade 3 or 4 adverse events than lenvatinib with everolimus or cabozantinib. HRQoL could not be analysed due to differences in tools used.

Conclusions

Lenvatinib with everolimus, cabozantinib and nivolumab are effective in prolonging survival for people with amRCC subsequent to VEGF-targeted treatment, but there is considerable uncertainty about how they compare to each other and how much better they are than the first generation of targeted treatments, axitinib and sorafenib.

Protocol registration: PROSPERO CRD42017071540

Data sharing statement: search strategies, data extraction form, risk of bias summaries, data inputs, NMA code and results tables are provided in a supplementary file.

ARTICLE SUMMARY

For peer review only

For peer review only - http://bmjopen.bmj.com/site/about/guidelines.xhtml

Strengths and limitations of this study

- This review is highly relevant and timely as it includes all recently approved treatments and focuses on the effectiveness of these treatments when used after first line VEGF-targeted tyrosine kinase inhibitor (TKI) treatment, as recommended in European clinical guidelines.
- The review focuses on high quality RCT evidence, but inclusion of comparative observational evidence in sensitivity analyses enabled estimates for axitinib and sorafenib, which otherwise could not be connected in the network.
- The reliability of the results of this review is hampered by trial design limitations of some of the included studies: the proportional hazards assumption did not hold for PFS in the one trial including nivolumab, RCT data for axitinib and sorafenib were limited to a subgroup analysis conducted in one study which could only be compared to the other treatments by including observational studies, and the trial assessing lenvatinib with everolimus is a small phase II trial with an increased risk of a false positive result and of over estimating the effect size due to some differences in baseline characteristics and relatively low significance level (alpha 0.15).
- There were also some differences between the trials in the network in terms of baseline characteristics, number and type of prior VEGF targeted treatments, and trial blinding, but there were too few studies to explore the potentially treatment modifying effects of these differences.

For peer review only - http://bmjopen.bmj.com/site/about/guidelines.xhtml

BACKGROUND

Kidney cancers are among the most common cancers in Europe (age-standardised rates estimated at 17.2/100,000 males and 8.1/100,000 females)[1] and renal cell carcinoma (RCC) makes up 80–90% of new cases. RCC occurs most commonly in men over 60 years, and smoking, obesity, hypertension, germline mutations and advanced kidney disease are established risk factors.[2] . RCC is often asymptomatic until later stages, so most people are diagnosed with advanced or metastatic disease (amRCC); five-year survival of amRCC is less than 10% and the goal of treatment is to slow progression and treat symptoms.[2]

Targeted treatments are designed to interrupt the biological pathways needed for the cancer to grow and spread. Since 2006, eight targeted treatments have been approved by the European Medicines Agency (EMA) for the treatment of amRCC,[3-10] falling within three classes: mammalian target of rapamycin inhibitors (mTORis; everolimus[5]), tyrosine kinase inhibitors (TKIs; sunitinib,[3] pazopanib,[7] axitinib,[6] cabozantinib,[8] lenvatinib[9] [in combination with the mTORi everolimus] and sorafenib[4]), and PD-1 monoclonal antibodies (nivolumab[10]). The mechanism of action of each treatment affects tolerability and has implications for treatment choice based on patient biomarkers.[11]

The emergence of targeted treatments has changed the RCC treatment pathway substantially and targeted treatments have virtually replaced the use of cytokines in many European health systems.[12] As a result, published studies assessing second-line targeted agents in populations who received first-line cytokines, or indeed adjusted indirect comparisons combining studies that enrolled those having received prior cytokines, have limited applicability to current

For peer review only
For peer review only - http://bmjopen.bmj.com/site/about/guidelines.xhtml

practice. Sunitinib and pazopanib (VEGFRs) are the only recommended first-line treatments in the latest RCC European Society for Medical Oncology (ESMO) clinical practice guidelines.[12] ESMO recommends axitinib, cabozantinib, sorafenib, everolimus, nivolumab, and lenvatinib with everolimus as treatment options from second line.[12]

Second-line practice patterns are not well established, partly because some treatments have only relatively recently been approved by the EMA.[13-15] Randomised controlled trials (RCTs), cohorts and patient registry data are emerging but head-to-head comparisons remain limited. Given the high cost of RCTs, and the number of treatments available for use at second line, it is unlikely that every treatment will ever be compared to every other treatment available. As such, adjusted indirect treatment comparisons are required to provide estimates beyond trial comparators to help establish an evidence-based treatment sequence for amRCC. Before cabozantinib, nivolumab and lenvatinib with everolimus were approved, network meta analyses (NMAs) of RCTs or good quality observational cohorts favoured axitinib and everolimus over sorafenib, though primarily within populations who had received prior cytokines.[16-19] Two NMAs of RCTs comparing more recently approved drugs indicate that lenvatinib with everolimus or cabozantinib are likely to be the most effective option to extend overall survival (OS) and progression-free survival (PFS) in amRCC. However, neither study included all the relevant treatments and both NMAs combine evidence for people who had either received prior cytokines or VEGF-targeted agents, reflecting an outdated pathway and unreliable results given that type of prior treatment is a potential treatment effect modifier.[20]

This systematic review is the first to include randomised and observational evidence for all recently-approved targeted treatments for amRCC, focusing specifically on the relevant

For peer review only - http://bmjopen.bmj.com/site/about/guidelines.xhtml

population who have previously received a VEGF-targeted treatment. By doing so, the review aims to provide a full and clinically relevant assessment of treatment safety and clinical effectiveness, focusing on outcomes that are the most important to patients (OS, PFS, overall response rate (ORR), quality of life, and adverse events).

OBJECTIVE

To compare the safety and clinical effectiveness of targeted treatments for amRCC previously treated with VEGF-targeted therapy.

METHODS

Methods for the review are reported in more detail in the published protocol (CRD42017071540) and were based on the principles published by the National Health Service Centre for Reviews and Dissemination.[21] The review reported here is an update and extension of a project commissioned by the UK National Institute for Health Research (NIHR), registered as CRD42016042384. This review was reregistered and updated to make the results applicable outside the UK and to include treatments that have received European marketing authorisation subsequent to publication of the first iteration of the review.

Patient and public involvement

Patients were not directly involved in the development of this review update but the original review was based on a scope produced by the National Institute for Health and Care Excellence (NICE) within which patients and patient groups were registered stakeholders.

Eligibility criteria

For peer review only - http://bmjopen.bmj.com/site/about/guidelines.xhtml

Study design

RCTs formed the basis of the primary analyses for all outcomes. As per the published protocol, comparative observational studies were included in sensitivity analyses for OS and PFS to provide a connected network for all interventions of interest. Preclinical studies, animal studies, narrative reviews, editorials, opinions and case reports were not eligible.

Population

Adults (18+ years) with a diagnosis of amRCC who had received previous treatment with a VEGF-targeted treatment.

Interventions

Interventions of interest were axitinib, cabozantinib, everolimus, lenvatinib with everolimus, nivolumab and sorafenib. Studies were included if they compared any of the listed interventions with each other, placebo or best supportive care (BSC). For the purposes of this review, placebo was assumed to be the equivalent of BSC. Studies comparing an intervention of interest with another treatment were only included if there were insufficient direct comparisons to provide a connected network that included all treatments of interest.

Outcomes

The primary outcomes were OS and PFS. Secondary outcomes were predefined as objective response rate (ORR), adverse events of Grade 3 and above (as defined by the Common Terminology Criteria for Adverse Events), and health-related quality of life (HRQoL).

Studies were excluded if none of the outcomes of interest were reported. Comparative observational studies were only included if they reported OS or PFS in a way that could be

For peer review only

For peer review only - http://bmjopen.bmj.com/site/about/guidelines.xhtml

incorporated into the NMA (i.e. as a hazard ratio [HR] or where a HR could be estimated from a Kaplan-Meier curve with the number of people at risk).

Search and selection process

Electronic searches for the original project were run in January 2016 (for RCTs; MEDLINE, EMBASE and CENTRAL) and June 2016 (observational studies; MEDLINE and EMBASE), and subsequently extended to cover a new intervention (lenvatinib with everolimus) and updated to January 2018. Manual searches of conference proceedings and bibliographies of included studies and systematic review were also updated to January 2018. Searches combined terms for the interventions of interest with condition terms for RCC and the relevant design filter (RCT or observational; example strategy provided in the Supplementary file [Table 1]). No date or language restrictions were applied. Searches for observational evidence were limited to interventions required to connect the network of treatments.

Unpublished and ongoing studies were identified by contacting experts in the field and searching ClinicalTrials.gov and the EU Clinical Trials Register.

Two reviewers screened all titles and abstracts independently. Full texts were retrieved and reviewed for records identified as potentially relevant by one or both reviewers. Discrepancies were resolved by consensus or by involving a third reviewer.

Data extraction and quality assessment

Data extraction was carried out independently by two reviewers and cross-checked for accuracy; as with study selection, discrepancies were resolved by discussion or by involving a third reviewer. A standard data extraction form was piloted and used to capture information

For peer review only

For peer review only - http://bmjopen.bmj.com/site/about/guidelines.xhtml

about study conduct, population, interventions, outcomes and risk of bias from each study, including the information source where more than one was available for a given study (template available in the Supplementary file [Table 2] together with extracted datasets for all outcomes). Where there were incomplete information study authors were contacted to gain further details.

Methodological quality was assessed independently by two reviewers using the Cochrane Risk of Bias tool for RCTs[22] and the ROBINS-I for comparative observational studies.[23] Where appropriate, risk of bias was assessed separately for each outcome within a study. Disagreements were resolved by consensus or by involving a third reviewer. The likely direction and magnitude of bias across the evidence as a whole was considered during interpretation of the results.

Data synthesis

Baseline characteristics of the included studies were compared to assess similarity of the study populations before combining results in an NMA. Fixed effects and random effects models were explored. However, as typically only one trial informed each pair-wise comparison and hence there were little data to inform the between trial heterogeneity, a pragmatic decision was made to use the fixed effects model for all outcomes. Statistical heterogeneity was assessed using the I^2 statistic for pairwise comparisons and deviance information criteria (DIC) for NMA. Inconsistency between direct and indirect effect estimates was assessed in closed loops in the network. Implications of observed clinical and statistical heterogeneity and inconsistency are described in the results.

For peer review only - http://bmjopen.bmj.com/site/about/guidelines.xhtml

Where NMA was possible, it was conducted according to the guidance described in the NICE Decisions Support Unit's Technical Support Documents for Evidence Synthesis.[24] A Bayesian Markov Chain Monte Carlo (MCMC) approach was taken in WinBUGS version 1.4.3 software[25] (codes included in the supplementary file) implementing uninformed priors and a burn-in of 30,000 iterations. Everolimus was specified as the baseline treatment. Data from multi-arm studies were adjusted to account for correlations in relative treatment effects.[26] OS and PFS were analysed as HRs, and response as odds ratios (ORs) using participants as the unit of analysis; no formal analysis could be performed for adverse effects or HRQoL due to between-study variation in reporting. A 95% Credible Interval can be interpreted as a 95% probability that the parameter falls within this range. If a 95% CrI doesn't include one this can, therefore, be interpreted as a statistically significant result (at the 5% level of significance). Primary analyses were based on studies of low, unclear or moderate risk of bias. Sensitivity analyses were planned for OS and PFS including RCTs of high risk of bias and observational studies of serious risk of bias. Observational studies at critical risk of bias were excluded from all analyses.

RESULTS

Results of the searches

Results of the original and update search and selection process are shown in Figure 1.

The searches carried out in June 2016 led to the inclusion of 44 records relating to 12 studies. Five of these studies have been excluded from this review because of the update of the scope excluding sunitinib as it is not recommended at second line in the most up-to-date ESMO

For peer review only

guidance for RCC.[12] Five new studies, one RCT and four retrospective chart reviews, were identified in the update and extension searches (including terms for lenvatinib with everolimus) run in January 2018, making a total of 12 included studies.[13, 15, 19, 20, 27-34]

Included studies

Twelve studies (n = 5,144) met the inclusion criteria (Table 1): five RCTs (one double-blind[29] and four open-label[13, 15, 20, 28]); and seven observational studies[19, 27, 30-34] (retrospective cohort studies). Sample sizes varied from 101 (HOPE 205[15]) to 821 (CheckMate 025[35]) participants.

For peer review only

For peer review only - http://bmjopen.bmj.com/site/about/guidelines.xhtml

Table 1. Study characteristics

Study	Design	Location, funding	Prior treatments	Intervention	N	Type	Median age years	Male %	ECOG 0/1 %	Treatment duration (follow-up) months
AXIS[20]	PIII OL RCT	175 sites in 22 countries, Pfizer	1 prior systemic treatment (sunitinib, cytokine or other), prior sunitinib subgroup 54%	Axitinib	361	CC	61	73	99	8.2 (NR)
				Sorafenib	362		61	71	100	5.2 (NR)
CheckMate 025[35]	PIII OL RCT	146 sites in 24 countries, BMS	1 or 2 prior targeted treatments (TKI or other, no mTORi)	Nivolumab	410	CC	62	77	NR	5.5 (NR)
				Everolimus	411		62	74		3.7 (NR)
HOPE 205[15]	PII OL RCT	37 sites in Czech Republic, Poland, Spain, UK, US, Eisai	1 prior TKI, no prior mTORi	Lenvatinib+eve	51	CC	61	69	100	7.6 (NR)
				Everolimus	50		59	76	100	4.1 (NR)
METEOR[13]	PIII OL RCT	173 sites in 26 countries, Exelixis	1 or more prior TKIs; no prior mTORi	Cabozantinib	330	CC	63*	77	100	8.3 (18.7)
				Everolimus	328		62*	73	100	4.4 (18.8)
RECORD-1[29]	PIII DB RCT, Novartis	86 sites in Australia, Canada Europe, Japan, US, Novartis	1 or 2 prior TKIs; no prior mTORi	Everolimus	277	CC	61*	78	NR	4.6 (NR)
				BSC/placebo	139		60*	76		1.9 (NR)
Guida 2017[33]	Chart review	1 site in France, NR	1 prior targeted treatment (TKI or other)	Everolimus	81	92% CC	57	69	85	NR (33)
				Axitinib	45		54	78	82	NR (26)
Heng 2016[19]	Chart review	UK, Germany, France, Netherlands, Novartis	1 prior TKI (sunitinib or pazopanib)	Everolimus	115	NR	60.2	66.7	91.8% ≤ 2	NR (NR)
				Axitinib	96					NR (NR)
				Sorafenib	98					NR (NR)
Iacovelli 2015[27]	Chart review	23 sites in Italy, NR	2 prior targeted treatments (TKI or other)	Sorafenib	90	CC	63	74	81	NR (NR)
				Everolimus	143					NR (NR)
Lakomy 2017[34]	Chart	Czech national	1 prior targeted	Everolimus	520	94%	65	75	95	6.1 (NR)

For peer review only - http://bmjopen.bmj.com/site/about/guidelines.xhtml

For peer review only

	review	registry, **	treatment (TKI or other)	Sorafenib	240	CC	62	75	90	7.1 (NR)
SPAZO-2[32]	Chart review	50 sites in Spain, Novartis	1 prior TKI (pazopanib)	Everolimus	101	88% CC	66	64	NR	NR (28)
				Axitinib	88		63	68		
Vogelzang 2016[30]	Chart review	US, Novartis	1 prior TKI; no prior cytokines	Everolimus	325	85% CC	61*	70	80	NR (15*)
				Axitinib	127		60*	65	84	NR (13*)
Wong 2014[31]	Chart review	US, Novartis	1 prior TKI; no prior mTORi, cytokines, bevacizumab	Everolimus	233	91% CC	64	70	NR	NR (12.9)
				Sorafenib	123		66	72		NR (12.1)

Abbreviations: NR, not reported; RCT, randomised controlled trials; BSC, best supportive care; mRCC, metastatic renal cell carcinoma; amRCC, advanced or metastatic RCC; cc, clear cell variant; ncc, non-clear cell variant; TKI, tyrosine kinase inhibitor; mTORi, mammalian target of rapamaycin inhibitor. Notes: ECOG percentages that do not total 100 are due to missing data;
*mean values where median was not reported.
** Ministry of Health of the Czech Republic, Central European Institute of Technology, The Ministry of Education, Youth and Sports. RENIS registry part funded by Pfizer, Bayer and Novartis.

For peer review only

For peer review only - http://bmjopen.bmj.com/site/about/guidelines.xhtml

All studies recruited adults with amRCC who had received at least one prior VEGF-targeted treatment. AXIS[20] also included people who had not received prior anti-VEGF treatment, but OS and PFS data were available for the subset who had. In eight of the included studies people had only received one prior VEGF-targeted treatment;[14, 19, 30-34, 36] the remaining five studies allowed one or more prior treatments.[13, 27, 35, 37] Populations were predominantly male and Caucasian, and mean age was generally between 55 and 65 years. Where reported, most people had stage 3 or 4 clear-cell RCC and the majority had baseline ECOG performance status of 0 or 1. Baseline characteristics were generally well balanced between treatment groups within trials, with the exception of HOPE 205[14], in which there were some imbalances in baseline characteristics, which may favour lenvatinib with everolimus over everolimus.

Where dose was reported, it was started at the standard licensed dose and adjusted according to clinical judgement. Treatment was reported in the RCTs to be continued until disease progression, unacceptable toxicity or withdrawal of consent, except for METEOR[13] and CheckMate 025[35] in which people could be treated beyond progression. Median treatment duration in the five studies where it was reported varied from 1.9 months (placebo [BSC] group of RECORD-1[29]) to 8.3 months (cabozantinib group of METEOR[13]). Median length of follow-up ranged from 12.1 months to 23.6 months, but was only reported in four studies.

Most studies gave limited information regarding treatments received subsequent to the study drug. In RECORD-1,[29] 76% of people randomised to placebo received open-label everolimus at progression, but the confounding of OS was reduced by using crossover-adjusted data in the NMA. Treatment crossover was not reported to have occurred in any other studies.

Treatments compared in each of the studies are shown in Table 1 and Figure 2. Direct comparisons made by RCTs are shown by black lines, and the additional connections possible

For peer review only - http://bmjopen.bmj.com/site/about/guidelines.xhtml

by incorporating comparative observational studies are shown with green lines; axitinib and sorafenib did not connect to the other treatments using only RCT evidence. Nivolumab could not be connected in the PFS network because it was not appropriate to analyse CheckMate 025[38] data with a Cox proportional hazards model.

Risk of bias

The five RCTs[13, 15, 20, 29, 38] were of good methodological quality; all are at low risk of bias for random sequence generation and allocation concealment. RECORD-1[29] was the only blinded study so there is a risk of performance bias in the others. In general, OS and PFS are considered low risk of detection and reporting biases for all RCTs except for a high risk of PFS detection bias in CheckMate 025[35] because it was not assessed by an independent review committee. None of the outcomes in the RCTs were at high risk of attrition bias; all used appropriate censoring for the time-to-event analyses, although OS data from CheckMate 025[35] and METEOR[13] are immature. Other possible sources of bias pertain to group differences in the rate and type of subsequent treatments received, which were poorly reported in most trials. RECORD-1[29] was the only trial allowing cross-over for people in the placebo arm, although cross-over adjusted results were reported. Despite appropriate randomisation in HOPE 205[15], which is a small phase II trial, there were some imbalances in the baseline characteristics of the people in the trial, which may indicate a better prognosis for the lenvatinib with everolimus group compared with everolimus alone. In addition, alpha was set to 0.15, compared to the usual 0.05, and HOPE 205 is therefore of a higher risk of a false positive result and possibly of over estimating the effect size.

For peer review only - http://bmjopen.bmj.com/site/about/guidelines.xhtml

The observational studies included in the OS and PFS sensitivity analyses are at a higher risk of bias than the RCTs. Overall ROBINS-I ratings were at best moderate, for OS,[19, 30, 32] and serious risk of bias for PFS. One study was at critical risk of bias for both PFS and OS,[34] which was excluded from the sensitivity analyses. In all studies the potential for inadequate control for confounding was thought to increase the risk of bias. All studies reporting PFS also had an increased risk of bias for this outcome due to the lack of standardised measurement for assessing progression and that outcome assessors were aware of the interventions.

One of the observational studies was publicly funded,[34] two studies did not report their funding source[27, 33] and the remaining observational studies and all RCTs were sponsored by various pharmaceutical companies. Risk of bias assessments for all included studies are provided in the supplementary file (Tables 3 and 4).

Overall survival

Lenvatinib with everolimus, cabozantinib and nivolumab all showed statistically significant benefits over the baseline treatment, everolimus, in in the primary OS analysis (Table 2). Lenvatinib with everolimus had the highest probability (61%) of being the most effective treatment out of those compared in the primary analysis. These results were mirrored in the sensitivity analysis including observational studies. The sensitivity analysis also suggests everolimus may be more effective than axitinib, sorafenib and BSC for overall survival. However, there is evidence of inconsistency between the direct and indirect evidence for axitinib, sorafenib and everolimus, which indicates that there is heterogeneity between the studies and highlights the uncertainty around the true estimates of the relative effect of these

For peer review only - http://bmjopen.bmj.com/site/about/guidelines.xhtml

treatments. Raw data for OS and all other outcomes are available in the supplementary file (Tables 5 to 9).

Table 2. Results of the network meta-analyses for the primary outcomes (OS and PFS) and grade 3 or 4 adverse events

	Primary NMA of RCTs		Sensitivity NMA of RCTs and observational studies
**Overall survival**	Probability most effective (%)	HR *versus* everolimus (95% credible interval)	
Lenvatinib+everolimus	61	**0.61 (0.36 to 0.96)**	**0.61 (0.36 to 0.96)**
Cabozantinib	28	**0.66 (0.53 to 0.82)**	**0.66 (0.53 to 0.83)**
Nivolumab	10	**0.74 (0.57 to 0.93)**	**0.74 (0.57 to 0.93)**
Axitinib	-	-	1.14 (0.95 to 1.37)
Sorafenib	-	-	1.38 (1.12 to 1.68)
BSC	2	1.90 (0.61 to 4.53)	1.90 (0.60 to 4.56)
**Progression-free survival**	Probability best (%)	HR *versus* everolimus (95% credible interval)	
Lenvatinib+everolimus	67	**0.47 (0.26 to 0.77)**	**0.47 (0.26 to 0.77)**
Cabozantinib	34	**0.51 (0.41 to 0.63)**	**0.51 (0.41 to 0.63)**
Axitinib	-	-	0.84 (0.70 to 1.00)
Sorafenib	-	**-**	1.17 (0.95 to 1.43)
BSC	0	3.06 (2.31 to 3.97)	3.06 (2.31 to 3.97)
**Grade 3 or 4 adverse events**	Probability least harmful (%)	OR *versus* everolimus (95% credible interval)	
Lenvatinib+everolimus	0	2.67 (1.05 to 5.68)	-
Cabozantinib	0	1.66 (1.18 to 2.27)	-
Nivolumab	100	0.40 (0.29 to 0.55)	-

Progression-free survival

As with OS, lenvatinib with everolimus and cabozantinib both showed statistically significant benefits over everolimus, and lenvatinib with everolimus had the highest probability (66.5%) of being the most effective treatment out of those compared in the primary analysis of PFS (Table 2). The results of the sensitivity analysis including observational study data indicate that axitinib also improves PFS compared with everolimus, whereas BSC leads to significantly shorter PFS compared with everolimus, and there was no statistically significant difference

For peer review only

For peer review only - http://bmjopen.bmj.com/site/about/guidelines.xhtml

between everolimus and sorafenib. For PFS there was no evidence of inconsistency between the direct and indirect evidence of axitinib, sorafenib and everolimus.

Nivolumab was not included in the analyses of PFS because the proportional hazards assumption does not hold for this outcome in CheckMate 025.[35]

Objective response rate

Two of the four RCTs that could be included in the NMA for ORR observed no events in one treatment arm (everolimus in HOPE 205[15] and BSC in RECORD-1[29, 37]), causing the results from the NMA to be unreliable and lack face validity. Results using a 0.5 correction for 0 values indicate that treatment with cabozantinib, lenvatinib with everolimus, and nivolumab all lead to a better response rates than treatment with everolimus, which in turn in significantly better than BSC (supplementary file, Table 10).

Adverse effects

In terms of safety, nivolumab had the highest probability of being least harmful, i.e. the rate of grade 3 or 4 AEs was significantly lower with nivolumab (18.7%) than with everolimus (36.5%),[35] whereas treatment with either cabozantinib or lenvatinib with everolimus resulted in significantly higher rates of grade 3 or 4 AEs than everolimus (METEOR[13]: cabozantinib 71.0%, everolimus 59.9%; HOPE 205[15]: lenvatinib + everolimus 71%, everolimus 50%). Rates of grade 3 or 4 AEs were not reported for axitinib or BSC in AXIS and RECORD-1.[36, 37]

Health-related quality of life

Treatments could not be compared using NMA for HRQoL as different measures and tools were used for assessments. HRQoL scores were similar between axitinib and sorafenib in

For peer review only - http://bmjopen.bmj.com/site/about/guidelines.xhtml

AXIS[36] and results favoured nivolumab over everolimus in CheckMate 025.[35] Results in RECORD-1[29] favoured BSC over everolimus, although this effect was only apparent if models were used to account for data not missing at random. METEOR[13] results were similar for everolimus and cabozantinib. HRQoL was not measured in HOPE 205[15]. A summary of results from each of the five RCTs is provided in the Supplementary File.

DISCUSSION

This systematic review and network meta-analysis suggests that lenvatinib with everolimus, cabozantinib and nivolumab all prolong PFS and are likely to increase OS compared to everolimus for people with amRCC previously treated with VEGF-targeted therapy. The results suggest lenvatinib with everolimus is likely to be the most effective treatment, followed by cabozantinib and then nivolumab, but there is considerable uncertainty around how they compare to each other and how much better they are than the first generation of targeted treatments, axitinib and sorafenib.Nivolumab may be associated with fewer grade 3 or 4 AEs than treatment with both lenvatinib with everolimus and cabozantinib. All treatments considered in this review appear to delay disease progression and prolong survival more than providing BSC, and results for ORR support the primary OS and PFS analyses. Due to differences in reporting and HRQoL tools used, it was not possible to perform NMAs on safety or HRQoL.

This is a robust and comprehensive systematic review and network meta-analysis based on the principles published by Centre for Reviews and Dissemination[21] using the MOOSE[39] and PRISMA[40] reporting guidelines, and conducted according to prespecified methods in a prospectively registered protocol (PROSPERO CRD42017071540). The inclusion of all recently

For peer review only - http://bmjopen.bmj.com/site/about/guidelines.xhtml

approved treatments increases the relevance and timeliness of the review. The review is also highly relevant as it focuses on the effectiveness and safety of these treatments when used after first line TKI treatment, as recommended in clinical guidelines.[12] However, there is not enough evidence available to answer questions about the sequencing of later lines of treatments.

Although this study focuses on high quality RCT evidence, the inclusion criteria were widened to incorporate comparative observational evidence in sensitivity analyses to enable estimates for axitinib and sorafenib, which otherwise could not be connected to the network.

However, the robustness of the evidence in this review is limited by several factors:

1) PFS for nivolumab compared with the other treatments could not be estimated in this review because the proportional hazards assumption didn't hold for this outcome in the one trial including nivolumab.[35]
2) Relevant RCT data for axitinib and sorafenib were limited to a subgroup analysis conducted in one study that did not connect to the network of other RCTs.[36] Axitinib and sorafenib could only be compared to the other treatment options by including observational studies which were generally at a serious risk of different kinds of bias.
3) The trial assessing the efficacy of lenvatinib with everolimus is a small phase II trial, with an alpha set to 0.15 and therefore a higher than usual risk of false positive results and overestimation of the treatment effect. In this trial there were also some differences in baseline characteristics likely to lead to an over estimation of the treatment effect of lenvatinib and everolimus compared with everolimus, which introduces uncertainty around the true treatment effect.

For peer review only - http://bmjopen.bmj.com/site/about/guidelines.xhtml

4) Although the baseline characteristics were well balanced within most of the trials, there were some differences in performance status and number of prior VEGF targeted treatments between the trials. There were also differences in trial design with some trials being double blind or open label. Outcome assessment was not always done by an independent review committee (IRC). However, in the nivolumab trial, CheckMate 025[35], progression was only assessed by non-blinded trial investigator. There were too few studies to explore the effects of these differences between studies, which is a limitation and increases the uncertainty of the results.
5) The number of studies identified prevented meaningful subgroup analyses to explore potentially important prognostic factors that varied across the included studies. For example, while the review was limited to populations who had received prior VEGF therapy, there was variation in eligibility and baseline criteria regarding the type of VEGF treatment received and number of prior lines (see Table 1).

Two NMAs of different subsets of treatments for previously treated amRCC have recently been published.[41, 42] Unlike these studies, this review provides an alternative approach and a comparison between all recently approved treatments. Rassy *et al.*[42] and Amzal *et al.*[41] combine evidence for people who had either received prior cytokines or VEGF-targeted agents. This enabled a connected network using only RCT data, but type of prior treatment has been shown to be a potential treatment effect modifier,[36] which could introduce bias into the analysis. In addition, results for people who have only had prior cytokines are less relevant to clinical practice than for prior VEGF-targeted treatments as most people receive a TKI first line, in line with clinical guidelines.[12] The NMAs of Amzal *et al.*[41] and Rassy *et al.*[42] are also

For peer review only - http://bmjopen.bmj.com/site/about/guidelines.xhtml

limited by the reliance on the TARGET trial[43] to link axitinib and sorafenib to the network analysed. TARGET[43] is an RCT of sorafenib and placebo in which people only had prior cytokines and not prior TKI. The results from the TARGET trial are also confounded by crossover, which has only been partly accounted for by using immature data censored at crossover, and the lack of proportional hazards between the trial arms for PFS and OS.

For the trials that are shared between Amzal *et al.*[41] and this review and Rassy *et al.*[42] and this review the order of treatments, in terms of OS and PFS, is similar. However, this systematic review focuses specifically on the most relevant population, who have previously received a VEGF-targeted treatment, and avoids the issues with the TARGET[43] trial by including both randomised and observational evidence, and thereby provides more relevant and reliable estimates of the relative efficacy between all the interventions.

Neither prior review planned to assessed ORR or HRQoL and so these outcomes cannot be compared with previous results. A narrative presentation of adverse events in Rassy *et al.*[42] is in line with our findings that lenvatinib with everolimus is likely to be less well tolerated than nivolumab; Rassy and colleagues highlight that similarly high proportions of patients experienced Grade 3–4 adverse events and discontinued treatment due to toxicity on cabozantinib and lenvatinib with everolimus, and the most commonly reported tolerability issues across treatments were fatigue and diarrhoea.

All treatments considered in this review delay disease progression and prolong survival more than BSC, and although this review gives an indication of the ranking of the most effective treatments for treating recurrent amRCC there is still much uncertainty around how much

For peer review only - http://bmjopen.bmj.com/site/about/guidelines.xhtml

these treatments differ from each other in terms of effectiveness and safety. The choice of treatment should take into account patient preference, comorbidities, symptoms, tumour burden and how aggressive the cancer is. Policy makers also need to consider the cost-effectiveness of the treatments.

It would be preferable to have high quality RCT data comparing all the available RCC treatment options, but this is unlikely to be commissioned due to the high costs of clinical trials. However, what is more likely and still needed is a larger RCT of lenvatanib with everolimus to confirm the efficacy data from the current phase II trial with its small sample size. RCT data of axitinib and sorafenib versus other comparators in the network are also required to enable higher quality evidence for these comparisons. As there is no cure for amRCC and as virtually all people progress, research is needed into the development of resistance to treatments. Further research is also required into the impact of different sequencing of drugs from second line and onwards as more people are well enough to tolerate additional lines of treatment and most of these drugs are approved for use also beyond second line (cabozantinib, everolimus, and nivolumab).

AUTHOR CONTRIBUTIONS

Charlotta Karner PhD validated data extraction, carried out and validated meta-analyses, and drafted and edited the manuscript. Kayleigh Kew MA (Cantab) carried out additional searches, contributed to the appraisal of title and abstracts, assessment of full publications for inclusion, data extraction and validation, carried out meta-analyses, and drafted the manuscript. Victoria Wakefield MBChB devised and carried out database searches, contributed to the appraisal of title and abstracts, assessment of full publications for

For peer review only - http://bmjopen.bmj.com/site/about/guidelines.xhtml

inclusion, and data extraction. Natalie Masento PhD contributed to the appraisal of title and abstracts, the assessment of full publications for inclusion, and data extraction. Steven Edwards DPhil supervised the production of the manuscript and acted as methodological advisor.

ACKNOWLEDGEMENTS

The authors would like to thank Clare Fiatikoski (BMJ) and George Osei-Assibey (BMJ) for their contribution to the original literature searching and data extraction underpinning this review, and Samantha Barton (BMJ) for providing comments on the draft manuscript. The authors would also like to thank Dr Amit Bahl (University Hospitals Bristol NHS Foundation Trust, Bristol), Dr Lisa Pickering (St Georges Hospital, London), Dr Sarah Rudman (Guys and St Thomas Hospital, London), Dr Penny Kechagioglou (University Hospitals Coventry and Warwickshire, Coventry), Professor Martin Gore (Royal Marsden NHS Foundation Trust, London) and Dr Naveed Sarwar (Imperial College Healthcare NHS Trust, London) for providing clinical advice throughout the original project.

COMPETING INTERESTS

No competing interests were declared which affect the impartiality of this report. BMJ-TAG and the editorial team of *The BMJ* work independently to one another.

FUNDING

The review presents an update and extension to independent research funded by the UK National Institute for Health Research (NIHR) health technology assessment programme (HTA 16/58/01). The views and opinions expressed in this report are those of the authors and not necessarily those of the NHS, the NIHR or the Department of Health and Social Care.

For peer review only - http://bmjopen.bmj.com/site/about/guidelines.xhtml

FIGURE LEGENDS

Figure 1. PRISMA diagram

Abbreviation: RCT, randomised controlled trials

Figure 2. Network diagram

For peer review only

For peer review only - http://bmjopen.bmj.com/site/about/guidelines.xhtml

REFERENCE LIST

1. Ferlay J, Steliarova-Foucher E, Lortet-Tieulent J, Rosso S, Coebergh JW, Comber H, et al. Cancer incidence and mortality patterns in Europe: estimates for 40 countries in 2012. *Eur J Cancer* 2013; **49**: 1374-403.

2. Cancer Research UK (CRUK). Kidney Cancer Statistics. *Available at: http://wwwcancerresearchukorg/health-professional/cancer-statistics/statistics-by-cancer-type/kidney-cancer#heading-Zero Accessed: 10 March 2016* 2015.

3. European Medicines Agency (EMA). Sutent (sunitinib) European public assessment report (EPAR), 2007. Available from: https://www.ema.europa.eu/documents/overview/sutent-epar-summary-public_en.pdf. Date accessed: 14 Nov 2018.

4. European Medicines Agency (EMA). Nexavar (sorafenib) European public assessment report (EPAR), 2009. Available from: https://www.ema.europa.eu/documents/overview/nexavar-epar-summary-public_en.pdf. Date accessed: 14 Nov 2018.

5. European Medicines Agency (EMA). Afinitor (everolimus) European public assessment report (EPAR), 2010. Available from: https://www.ema.europa.eu/documents/overview/afinitor-epar-summary-public_en.pdf. Date accessed: 14 Nov 2018.

6. European Medicines Agency (EMA). Inlyta (axitinib) European public assessment report (EPAR), 2012. Available from: https://www.ema.europa.eu/documents/overview/inlyta-epar-summary-public_en.pdf. Date accessed: 14 Nov 2018.

7. European Medicines Agency (EMA). Votrient (pazopanib) European public assessment report (EPAR), 2013. Available from: https://www.ema.europa.eu/documents/overview/votrient-epar-summary-public_en.pdf. Date accessed: 14 Nov 2018.

8. European Medicines Agency (EMA). Cabometyx (cabozantinib) European public assessment report (EPAR), 2016. Available from: https://www.ema.europa.eu/documents/overview/cabometyx-epar-medicine-overview_en.pdf. Date accessed: 13 Nov 2018.

9. European Medicines Agency (EMA). Kisplyx (lenvatinib) European public assessment report (EPAR), 2016. Available from: https://www.ema.europa.eu/documents/overview/kisplyx-epar-summary-public_en.pdf. Date accessed: 14 Nov 2018.

10. European Medicines Agency (EMA). Opdivo (nivolumab) European public assessment report (EPAR), 2015. Available from: https://www.ema.europa.eu/documents/overview/opdivo-epar-summary-public_en.pdf. Date accessed: 14 Nov 2018.

11. Rodriguez-Vida A, Strijbos M, Hutson T. Predictive and prognostic biomarkers of targeted agents and modern immunotherapy in renal cell carcinoma. *ESMO Open* 2016; **1**: e000013.

12. Escudier B, Porta C, Schmidinger M, Rioux-Leclercq N, Bex A, Khoo V, et al. Renal cell carcinoma: ESMO Clinical Practice Guidelines for diagnosis, treatment and follow-up. *Ann Oncol* 2016; **27**: v58 – v68.

For peer review only - http://bmjopen.bmj.com/site/about/guidelines.xhtml

13. Choueiri T, Escudier B, Powles T, Tannir N, Mainwaring P, Rin iB. Cabozantinib versus everolimus in advanced renal cell carcinoma (METEOR): final results from a randomised, open-label, phase 3 trial. *The Lancet Oncology* 2016; **17**: 917-27.
14. Motzer RJ, Escudier B, McDermott DF, George S, Hammers HJ, Srinivas S, et al. Nivolumab versus Everolimus in Advanced Renal-Cell Carcinoma. *New England Journal of Medicine* 2015; **373**: 1803-13.
15. Motzer RJ, Hutson TE, Glen H, Michaelson MD, Molina A, Eisen T, et al. Lenvatinib, everolimus, and the combination in patients with metastatic renal cell carcinoma: a randomised, phase 2,open-label, multicentre trial. *Lancet Oncol* 2015; **16**: 1473-82.
16. Larkin J, Paine A, Tumur I, Cappelleri JC, Healey PJ, Sr., Foley G, et al. Second-line treatments for the management of advanced renal cell carcinoma: systematic review and meta-analysis. *Expert Opin Pharmacother* 2013; **14**: 27-39.
17. Dranitsaris G, Schmitz S, Broom RJ. Small molecule targeted therapies for the second-line treatment for metastatic renal cell carcinoma: a systematic review and indirect comparison of safety and efficacy. *J Cancer Res Clin Oncol* 2013; **139**: 1917-26.
18. Wang H, Man L, Li G, Huang G, Wang J. Comparative efficacy and safety of axitinib versus sorafenib in metastatic renal cell carcinoma: a systematic review and meta-analysis. *Onco Targets Ther* 2016; **9**: 3423-32.
19. Heng DY, Signorovitch J, Swallow E, Li N, Zhong Y, Qin P, et al. Comparative Effectiveness of Second-Line Targeted Therapies for Metastatic Renal Cell Carcinoma: A Systematic Review and Meta-Analysis of Real-World Observational Studies. *PLoS ONE [Electronic Resource]* 2014; **9**: e114264.
20. Motzer RJ, Escudier B, Tomczak P, Hutson TE, Michaelson MD, Negrier S, et al. Axitinib versus sorafenib as second-line treatment for advanced renal cell carcinoma: overall survival analysis and updated results from a randomised phase 3 trial, 2013. Available from: http://onlinelibrary.wiley.com/o/cochrane/clcentral/articles/055/CN-00864055/frame.html. Date accessed.
21. Centre for Reviews and Dissemination (CRD). CRD's guidance for undertaking reviews in healthcare, 2011. Date accessed: 24 July 2017.
22. Higgins JP, Altman DG, Gotzsche PC, Juni P, Moher D, Oxman AD, et al. The Cochrane Collaboration's tool for assessing risk of bias in randomised trials. *Bmj* 2011; **343**: d5928.
23. Sterne JAC, Hernán MA, Reeves BC, Savović J, Berkman ND, Viswanathan M, et al. ROBINS-I: a tool for assessing risk of bias in non-randomized studies of interventions. *BMJ* 2016; **355**: i4919.
24. Dias S, Welton NJ, Sutton AJ, Ades AE. NICE DSU Technical Support Document 1: introduction to evidence synthesis for decision making. *Available at http://wwwnicedsuorguk/TSD1%20Introductionfinal080512pdf Accessed 7 March 2017* 2011 (updated 2012).
25. Lunn DJ, Thomas A, Best N, Spiegelhalter D. WinBUGS – A Bayesian modelling framework: Concepts, structure, and extensibility. *Statistics and Computing* 2000; **10**: 325-37.
26. Woods BS, Hawkins N, Scott DA. Network meta-analysis on the log-hazard scale, combining count and hazard ratio statistics accounting for multi-arm trials: a tutorial. *BMC Med Res Methodol* 2010; **10**: 54.

For peer review only - http://bmjopen.bmj.com/site/about/guidelines.xhtml

27. Iacovelli R, Santini D, Rizzo M, Felici A, Santoni M, Verzoni E, et al. Bone metastases affect prognosis but not effectiveness of third-line targeted therapies in patients with metastatic renal cell carcinoma. *Canadian Urological Association Journal* 2015; **9**: 263-7.
28. Motzer RJ, Escudier B, McDermott DF, George S, Hammers HJ, Srinivas S, et al. Nivolumab versus Everolimus in Advanced Renal-Cell Carcinoma, 2015. Available from: http://onlinelibrary.wiley.com/o/cochrane/clcentral/articles/537/CN-01108537/frame.html. Date accessed.
29. Motzer RJ, Escudier B, Oudard S, Hutson TE, Porta C, Bracarda S, et al. Efficacy of everolimus in advanced renal cell carcinoma: a double-blind, randomised, placebo-controlled phase III trial, 2008. Available from: http://onlinelibrary.wiley.com/o/cochrane/clcentral/articles/982/CN-00649982/frame.html. Date accessed.
30. Vogelzang NJ, Pal SK, Signorovitch JE, Reichmann WM, Li N, Yang C, et al. Comparative effectiveness of everolimus and axitinib as second targeted therapies for metastatic renal cell carcinoma in the US: A retrospective chart review. *Current Medical Research and Opinion* 2016; **32**: 741-7.
31. Wong MK, Yang H, Signorovitch JE, Wang X, Liu Z, Liu NS, et al. Comparative outcomes of everolimus, temsirolimus and sorafenib as second targeted therapies for metastatic renal cell carcinoma: A US medical record review. *Curr Med Res Opin* 2014; **30**: 537-45.
32. Arranz Arija J, Perez-Valderrama B, Rodriguez Sanchez A, Puertas Alvarez J, Pinto Marin A, Maximiano Alonso C, et al. SPAZO2 (SOGUG): Comparative effectiveness of everolimus (Ev) vs axitinib (Ax) as second-line after first-line pazopanib (1stPz) in metastatic renal carcinoma (mRC). *Ann Oncol* 2017; **28 (Supplement 5)**: v313.
33. Guida A, Albiges L, Derosa L, Loriot Y, Massard C, Fizazi K, et al. Everolimus Versus Axitinib as Second-line Therapy in Metastatic Renal Cell Carcinoma: Experience From Institut Gustave Roussy. *Clin Genitourin Cancer* 2017; **15**: e1081-e8.
34. Lakomy R, Poprach A, Bortlicek Z, Melichar B, Chloupkova R, Vyzula R, et al. Utilization and efficacy of second-line targeted therapy in metastatic renal cell carcinoma: Data from a national registry. *BMC Cancer* 2017; **17 (1) (no pagination)**.
35. Motzer RJ, Escudier B, McDermott DF, George S, Hammers HJ, Srinivas S, et al. Nivolumab versus Everolimus in Advanced Renal-Cell Carcinoma. *N Engl J Med* 2015; **373**: 1803-13.
36. Motzer RJ, Escudier B, Tomczak P, Hutson TE, Michaelson MD, Negrier S, et al. Axitinib versus sorafenib as second-line treatment for advanced renal cell carcinoma: overall survival analysis and updated results from a randomised phase 3 trial. *Lancet Oncol* 2013; **14**: 552-62.
37. Motzer RJ, Escudier B, Oudard S, Hutson TE, Porta C, Bracarda S, et al. Phase 3 trial of everolimus for metastatic renal cell carcinoma : final results and analysis of prognostic factors, 2010. Available from: http://onlinelibrary.wiley.com/o/cochrane/clcentral/articles/159/CN-00762159/frame.html. Date accessed.
38. Motzer R, Sharma P, McDermott D, George S, HJ H, Srinivas S, editors. CheckMate 025 phase III trial: Outcomes by key baseline factors and prior therapy for nivolumab (NIVO) versus everolimus (EVE) in advanced renal cell carcinoma (RCC). ESMO 2016; 2016; Copenhagen: Journal of Clinical Oncology.

For peer review only - http://bmjopen.bmj.com/site/about/guidelines.xhtml

39. Stroup DF, Berlin JA, Morton SC, Olkin I, Williamson GD, Rennie D, et al. Meta-analysis of observational studies in epidemiology: a proposal for reporting. Meta-analysis Of Observational Studies in Epidemiology (MOOSE) group. *JAMA* 2000; **283**: 2008-12.
40. Moher D, Liberati A, Tetzlaff J, Altman DG, Group P. Preferred reporting items for systematic reviews and meta-analyses: the PRISMA statement. *BMJ* 2009; **339**: b2535.
41. Amzal B, Fu S, Meng J, Lister J, Karcher H. Cabozantinib versus everolimus, nivolumab, axitinib, sorafenib and best supportive care: A network meta-analysis of progression-free survival and overall survival in second line treatment of advanced renal cell carcinoma. *PLoS One* 2017; **12**: e0184423.
42. El Rassy E, Aoun F, Sleilaty G, Kattan J, Banyurwabuke B, Zanaty M, et al. Network meta-analysis of second-line treatment in metastatic renal cell carcinoma: efficacy and safety. *Future Oncol* 2017; **13**: 2709-17.
43. Escudier B, Eisen T, Stadler WM, Szczylik C, Oudard S, Siebels M, et al. Sorafenib in advanced clear-cell renal-cell carcinoma. *N Engl J Med* 2007; **356**: 125-34.

For peer review only

For peer review only - http://bmjopen.bmj.com/site/about/guidelines.xhtml

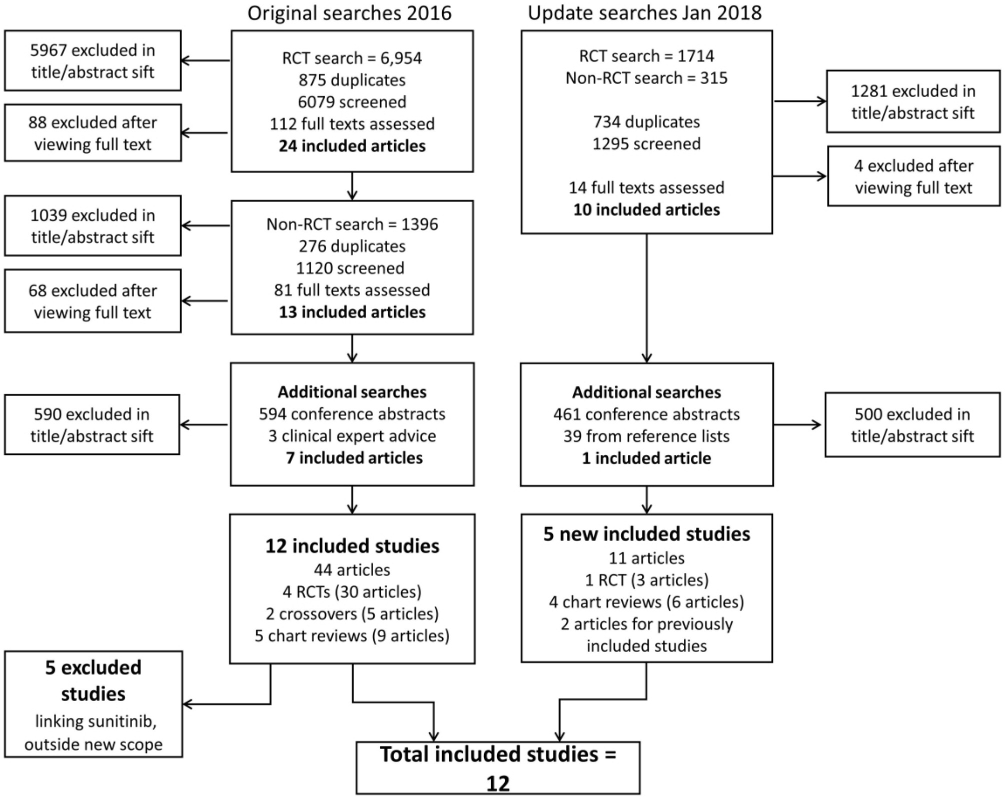

Figure 1. PRISMA diagram

288x243mm (300 x 300 DPI)

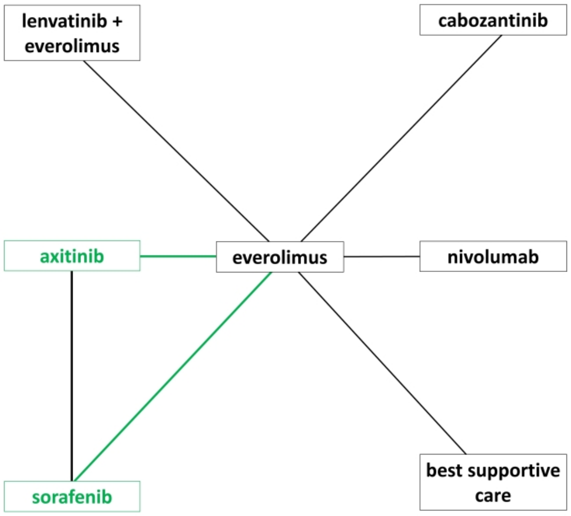

Figure 2. Network diagram

513x289mm (300 x 300 DPI)

SUPPLEMENTARY FILE

Targeted therapies for previously treated advanced or metastatic renal cell carcinoma: systematic review and network meta-analysis

Contents

SEARCH STRATEGY 2
DATA EXTRACTION 4
RISK OF BIAS SUMMARIES 7
DATASETS 9
WINBUGS CODE 12
RESULTS 14
REFERENCES 15

For peer review only

For peer review only - http://bmjopen.bmj.com/site/about/guidelines.xhtml

SUPPLEMENTARY FILE

Targeted therapies for previously treated advanced or metastatic renal cell carcinoma: systematic review and network meta-analysis

SEARCH STRATEGY

Table 1. Example search strategy (EMBASE update search for randomised controlled trials)

OVID: EMBASE 1974 to July 03 (searched on 4th July 2017 from Week 3 2016 to Week 27 2017)		
#	Search Terms	Results
1	Carcinoma, Renal Cell/	20712
2	(renal cell carcinoma$ or cell renal carcinoma$ or renal carcinoma$ or kidney carcinoma$ or kidney cell carcinoma$ or renal adenocarcinoma$ or kidney adenocarcinoma$ or adenocarcinoma$renal or adenocarcinoma$kidney$).mp.	66950
3	(hypernephroma$ or nephroid carcinoma$ or hypernephroid carcinoma$ or kidney hypernephroma$ or kidney pelvic carcinoma$ or kidney pyelocarcinoma$ or renal hypernephroma$ or grawitz tumo?r$ or renal cell neoplasm$ or renal cell cancer$ or renal tumo?r$ or carcinoma chromophobe cell kidney$ or chromophobe cell kidney carcinoma$).mp.	17922
4	kidney neoplasms/	10255
5	(cancer$ adj2 kidney$1).ti,ab.	5836
6	(neoplasm$1 adj2 kidney$1).ti,ab.	329
7	(neoplasm$1 adj2 renal).ti,ab.	2153
8	(cancer$ adj2 renal).ti,ab.	12586
9	(tumo?r$1 adj2 kidney$1).ti,ab.	4838
10	(tumo?r$1 adj2 renal).ti,ab.	14674
11	or/1-10	92199
12	(axitinib or inlyta or AG013736 or "AG 013736").mp.	3492
13	(sorafenib or nexavar or bay 43-9006 or bay 439006 or bay43-9006 or bay439006).mp.	23166
14	(sunitinib or sutent or pha 2909040ad or pha2909040ad or "su 010398" or "su 011248" or su 10398 or su10398 or su 11248 or su010398 or su011248 or su11248).mp.	18935
15	(everolimus or afinitor or certican or zortress or nvp-rad-001 or rad-001 or rad 001a or rad001 or rad001a or sdz rad).mp.	23010
16	(nivolumab or opdivo or ONO4538 or ONO 4538 or BMS936558 or BMS 936558 or MDX1106 or MDX 1106).mp.	4666
17	(temsirolimus or cci-779 or cell-cycle-inhibitor-779 or nsc 683864 or nsc683864 or torisel).mp.	7267
18	(bevacizumab or avastin or nsc 704865 or nsc704865 or anti-vegf or rhumab-vegf).mp.	50865
19	(armala or pazopanib or gw786034 or gw 786034 or sb 710468 or sb710468 or votrient).mp.	5612
20	or/12-19	99220
21	Clinical trial/	934498
22	Randomized controlled trial/	460454
23	Randomization/	74486
24	Single blind procedure/	28210
25	Double blind procedure/	140589
26	Crossover procedure/	52369
27	Placebo/	309726
28	Randomi?ed controlled trial$.tw.	162409
29	Rct.tw.	24817
30	Random allocation.tw.	1704
31	Randomly allocated.tw.	28013
32	Allocated randomly.tw.	2271
33	(allocated adj2 random).tw.	867
34	Single blind$.tw.	19692
35	Double blind$.tw.	180274
36	((treble or triple) adj blind$).tw.	721
37	Placebo$.tw.	257991
38	Prospective study/	388181

For peer review only - http://bmjopen.bmj.com/site/about/guidelines.xhtml

SUPPLEMENTARY FILE

Targeted therapies for previously treated advanced or metastatic renal cell carcinoma: systematic review and network meta-analysis

39	or/21-38	1782373
40	Case study/	48285
41	Case report.tw.	342862
42	Abstract report/ or letter/	1025233
43	or/40-42	1408381
44	39 not 43	1736655
45	11 and 20 and 44	3942
46	Animals/ not Humans/	1295518
47	45 not 46	3942
48	(editorial or letter).pt.	1521255
49	47 not 48	3868
50	limit 49 to em=201603-201727	327

For peer review only

For peer review only - http://bmjopen.bmj.com/site/about/guidelines.xhtml

SUPPLEMENTARY FILE

Targeted therapies for previously treated advanced or metastatic renal cell carcinoma: systematic review and network meta-analysis

DATA EXTRACTION

Table 2. Data extraction template

**Study or trial name:**			**Publication source**
**Full reference for all publications:**			
**Design**			
Study design			
Number of centres & Country/countries			
Recruitment dates			
Length of follow-up [include study start date, data cut-off and completion date]			
Source of funding			
Eligibility criteria (inclusion and exclusion)			
**Participants and treatment arms**	**Intervention:**	**Comparator:**	**Publication, data cut-off**
Intervention, method of delivery, dose and frequency			
Concomitant medication(s) or therapies			
Cross-over or post-study interventions allowed<br>Number of patients (%)			
Number of cycles<br>At least one dose reduction n (%)			
Treatment duration (and the data cut offs for each publication for the study)			
Number randomised			
Number who received study medication			
Number withdrawn/ discontinued and reasons<br>[give breakdown]			
Disease stage and/or metastatic disease<br>[give breakdown]			
Previous systemic therapy treatments, n (%) [give breakdown]			
Age, years: median (range)			
Ethnicity, n (%)<br>[give breakdown]			
Male, n (%)			
Performance status n (%)			

For peer review only

For peer review only - http://bmjopen.bmj.com/site/about/guidelines.xhtml

SUPPLEMENTARY FILE

Targeted therapies for previously treated advanced or metastatic renal cell carcinoma: systematic review and network meta-analysis

[give tool and breakdown]			
Reported subgroups	None reported		
**Reported outcomes**			
Primary outcome			
Secondary outcomes			
Outcomes and time points with data reported for subgroups of prior baseline therapies			
Outcomes and time points with data reported for subgroups of baseline prognostic scores (e.g. ECOG, MSKCC)			
**Results**	**Intervention**	**Comparator**	**Publication, data cut-off**
**PFS**			
HR (95% CI)			
HR (95% CI) for subgroups based on prior therapy:			
PFS, median (95% CI) months			
PFS, median (95% CI), months for subgroups based on prior therapy			
Number of progression events n (%)			
**Overall survival**			
HR, (95% CI)			
HR, (95% CI) for subgroups based on prior therapy			
Number of deaths, n (%)			
Median OS, months (95% CI)			
Median OS, (95% CI) months for subgroup based on prior therapy			
Number of deaths, n (%) for subgroups based on prior therapy			
**Response**			
Objective response, n (%)			
Complete response, n (%)			
Partial response, n (%)			
Stable disease, n (%)			
Progressive disease, n (%)			
Time to response, months (median [range])			
Duration of response, median (95% CI), months			
Other measures of response			
**HRQoL**			

For peer review only

For peer review only - http://bmjopen.bmj.com/site/about/guidelines.xhtml

SUPPLEMENTARY FILE

Targeted therapies for previously treated advanced or metastatic renal cell carcinoma: systematic review and network meta-analysis

[Scale 1] Mean end of treatment			
[Scale 1] Mean difference (95% CI)			
Completion rate			
**Adverse events (AE's)**			
N in safety analysis			
Total AE's (any Grade)			
Total AE's Grade ≥3			
[enter list of individual AEs]			

**Risk of bias assessment based (RCTs)**		
**Domain**	**Risk assessment**	**Comments**
Random sequence generation	[Low/High/Unclear]	
Allocation concealment	[Low/High/Unclear]	
Blinding (who [participants, personnel], and method)	[Low/High/Unclear]	
Other biases	[Low/High/Unclear]	
Progression-free survival		
-Blinding of outcome assessment	[Low/High/Unclear]	
-Incomplete outcome data	[Low/High/Unclear]	
-Selective reporting	[Low/High/Unclear]	
Overall survival		
-Blinding of outcome assessment	[Low/High/Unclear]	
-Incomplete outcome data	[Low/High/Unclear]	
-Selective reporting	[Low/High/Unclear]	
Response (partial response, disease stabilisation, progressive disease)		
-Blinding of outcome assessment	[Low/High/Unclear]	
-Incomplete outcome data	[Low/High/Unclear]	
-Selective reporting	[Low/High/Unclear]	
HRQoL		
-Blinding of outcome assessment	[Low/High/Unclear]	
-Incomplete outcome data	[Low/High/Unclear]	
-Selective reporting	[Low/High/Unclear]	
Adverse events		
-Blinding of outcome assessment	[Low/High/Unclear]	
-Incomplete outcome data	[Low/High/Unclear]	
-Selective reporting	[Low/High/Unclear]	

For peer review only - http://bmjopen.bmj.com/site/about/guidelines.xhtml

SUPPLEMENTARY FILE

Targeted therapies for previously treated advanced or metastatic renal cell carcinoma: systematic review and network meta-analysis

RISK OF BIAS SUMMARIES

Table 3. Summary of Cochrane risk of bias assessment for randomised control trials

Criteria		AXIS	Checkmate -025	HOPE 205	METEOR	RECORD-1
All outcomes	Random sequence generation	✓	✓	✓	✓	✓
	Allocation concealment	✓	✓	✓	✓	✓
	Blinding: participant/personnel	x	X	X	X	✓
**Outcome-specific**						
OS	Blinding: outcome assessment	✓	✓	✓	✓	✓
	Incomplete outcome data	✓	?	✓	?	✓
	Selective Reporting	✓	✓	✓	✓	✓
	Other Biases	?	?	?	?	?
PFS	Blinding: outcome assessment	✓	X	✓	✓	✓
	Incomplete outcome data	✓	✓	✓	?	✓
	Selective Reporting	✓	✓	✓	✓	✓
	Other Biases	NA	NA	?	NA	NA
ORR	Blinding: outcome assessment	✓	X	✓	✓	✓
	Incomplete outcome data	?	✓	✓	?	✓
	Selective Reporting	✓	✓	✓	✓	?
	Other Biases	NA	NA	?	NA	NA
HRQoL	Blinding: outcome assessment	x	X	NA	X	x
	Incomplete outcome data	✓	✓	NA	?	✓
	Selective Reporting	✓	✓	NA	✓	x
	Other Biases	NA	NA	NA	NA	NA
AE	Blinding: outcome assessment	x	X	X	X	✓
	Incomplete outcome data	✓	✓	✓	✓	✓
	Selective Reporting	✓	✓	✓	✓	✓
	Other Biases	NA	NA	?	NA	NA

Key: ✓, low risk; ?, unclear risk; X, high risk; NA, not applicable.
Abbreviations: OS, overall survival; PFS, progression-free survival; ORR, objective response rate; HRQoL, health-related quality of life; AE, adverse effects.

For peer review only - http://bmjopen.bmj.com/site/about/guidelines.xhtml

SUPPLEMENTARY FILE

Targeted therapies for previously treated advanced or metastatic renal cell carcinoma: systematic review and network meta-analysis

Table 4. Summary of ROBINS-I risk of bias assessments in non-randomised studies

	Guida 2017	Heng 2016	Iacovelli 2015	Lakomy 2017	SPAZO-2	Vogelzang 2016	Wong 2014
**Overall survival**							
Confounding	~	~	x	x	~	~	~
Selection	✓	✓	✓	✓	✓	✓	✓
Intervention classification	✓	✓	✓	✓	✓	✓	✓
Intervention deviations	✓	✓	✓	✓	✓	✓	✓
Missing data	✓	NI	✓	✓	✓	✓	x
Outcome measures	✓	✓	✓	✓	✓	✓	✓
Outcome reporting	✓	✓	✓	✓	✓	✓	✓
**Overall judgement**	x	~	x	x	~	~	x
**Progression-free survival**							
Confounding	x	~	-	x	~	~	~
Selection	✓	✓	-	✓	✓	✓	✓
Intervention classification	✓	✓	-	✓	✓	✓	✓
Intervention deviations	✓	✓	-	✓	✓	✓	✓
Missing data	✓	NI	-	✓	✓	✓	x
Outcome measures	x	x	-	x	x	x	x
Outcome reporting	x	x	-	x	x	x	x
**Overall judgement**	x	x	-	x	x	x	x

Abbreviations: PFS = progression-free survival; OS = overall survival

Key: ✓, low risk; ~, moderate risk; x, serious risk; x, critical risk; NI, no information.

For peer review only

SUPPLEMENTARY FILE

Targeted therapies for previously treated advanced or metastatic renal cell carcinoma: systematic review and network meta-analysis

DATASETS

Table 5. Study data: overall survival

Study	Data details	T1	T2	HR (95% CI)	T1 median months (95% CI)	T2 median (95% CI)
**AXIS[1]**	**Prior sunitinib subset**	**5**	**6**	**1.00 (0.78 to 1.27)**	**NR**	**NR**
**CheckMate 025[2]**	**-**	**3**	**1**	**0.73 (0.57 to 0.93)**	**25.0 (21.8 to NE)**	**19.6 (17.6 to 23.1)**
**HOPE 205[3]**	**July 2015 cutoff**	**7**	**1**	**0.59 (0.36 to 0.97)**	**25.5 (16.4 to 32.1)**	**15.4 (11.8 to 20.6)**
**METEOR[4]**	**31 Dec 2015 cutoff**	**2**	**1**	**0.66 (0.53 to 0.83)**	**21.4 (18.7 to NE)**	**16.5 (14.7 to 18.8)**
**RECORD-1[5]**	**RPSFT adjusted**	**1**	**4**	**0.60 (0.22 to 1.65)**	**14.4 (NR)**	**10.0 (NR)**
Guida 2017[6]*		5	1	1.33 (0.8 to 2.1)	14.9 (7.4 to 22.4)	21.5 (16.5 to 26.5)
Heng 2016[7]	-	6	1	1.25 (0.75 to 2.10)	18.7 (NR)	23.0 (NR)
Heng 2016[7]	-	5	1	1.22 (0.77 to 1.94)	23.5 (NR)	23.0 (NR)
Iacovelli 2015[8]	-	6	1	2.21 (1.47 to 3.31)	NR	NR
SPAZO-2[9]	Adjusted results	5	1	0.81 (0.60 to 1.20)	11.6 (7 to 16)	9.5 (7 to 12)
Vogelzang 2016[10]	-	1	5	1.16 (0.74 to 1.82)	NR	NR
Wong 2014[11]	Full adjusted results	1	6	0.66 (0.44 to 0.99)	19.0 (NR)	13.8 (NR)

Abbreviations: T1, treatment 1; T2, treatment 2 (baseline); HR, hazard ratio; CI, confidence interval; NE, not estimable; RPSFT, rank preserving structural failure time model; NR, not reported.
Treatment codes: 1, everolimus; 2, cabozantinib; 3, nivolumab; 4, best supportive care/placebo; 5, axitinib; 6, sorafenib; 7, lenvatinib with everolimus. Studies in bold formed the primary analysis.
*Data from personal communication with the study author, 11 March 2018

Table 6. Study data: progression-free survival

Study	Data details	T1	T2	HR (95% CI)	T1 median months (95% CI)	T2 median (95% CI)
**HOPE 205[12]**	**IRR**	**7**	**1**	**0.45 (0.27 to 0.79)**	**12.8 (7.4 to 17.5)**	**5.6 (3.6 to 9.3)**
**METEOR[4]**	**IRR for ITT**	**2**	**1**	**0.51 (0.41 to 0.62)**	**7.4 (6.6 to 9.1)**	**3.9 (3.7 to 5.1)**
**RECORD-1[13]**	**Final analysis, ICR**	**1**	**4**	**0.33 (0.25 to 0.43)**	**4.9 (4.0 to 5.5)**	**1.9 (1.8 to 1.9)**
AXIS[1]	Prior sunitinib subset	5	6	0.72 (0.57 to 0.90)	6.5 (5.7 to 7.9)	4.4 (2.9 to 4.7)
Guida 2017[6]*		5	1	0.84 (0.55 to 1.2)	7.7 (5.3 to 10.2)	5.3 (4.0 to 6.6)
Heng 2016[7]	-	6	1	1.47 (0.95 to 2.28)	-	-
Heng 2016[7]	-	5	1	1.26 (0.81 to 1.95)	-	-
Iacovelli 2015[8]	-	6	1	NR	NR	NR
SPAZO-2[9]	Adjusted results	5	1	0.76 (0.5 to 1.1)	5.3 (3 to 7)	4.6 (3 to 6)

For peer review only - http://bmjopen.bmj.com/site/about/guidelines.xhtml

SUPPLEMENTARY FILE

Targeted therapies for previously treated advanced or metastatic renal cell carcinoma: systematic review and network meta-analysis

Vogelzang 2016[10]	Adjusted results	1	5	1.16 (0.85 to 1.59)	NR	NR
Wong 2014[11]	Adjusted results	1	6	0.76 (0.55 to 1.04)	10.1 (NR)	8.6 (NR)

Abbreviations: T1, treatment 1; T2, treatment 2 (baseline); HR, hazard ratio; CI, confidence interval; NR, not reported.
Treatment codes: 1, everolimus; 2, cabozantinib; 3, nivolumab; 4, best supportive care/placebo; 5, axitinib; 6, sorafenib; 7, lenvatinib with everolimus. Studies in bold formed the primary analysis.
*Data from personal communication with the study author, 11 March 2018

Table 7. Study data: objective response rate

			N		Objective response	
**Study**	**T1**	**T2**	**T1**	**T2**	**T1**	**T2**
AXIS[14]	5	6	361	362	70	34
CheckMate 025[2]	3	1	410	411	103	22
HOPE 205[12]	7	1	51	50	18	0
METEOR[4]	2	1	330	328	57	11
RECORD-1[13]	1	4	277	139	5	0

Abbreviations: T1, treatment 1; T2, treatment 2 (baseline).
Treatment codes: 1, everolimus; 2, cabozantinib; 3, nivolumab; 4, best supportive care/placebo; 5, axitinib; 6, sorafenib; 7, lenvatinib with everolimus

Table 8. Study data: grade 3 or 4 adverse events

			N		Grade 3 or 4 adverse events	
**Study**	**T1**	**T2**	**T1**	**T2**	**T1**	**T2**
AXIS[1]	5	6	359	355	NR	NR
CheckMate 025[2]	3	1	406	397	76	145
HOPE 205[15]	7	1	51	50	36	25
METEOR[4]	2	1	331	322	235	193
RECORD-1[13]	1	4	274	137	NR	NR

Abbreviations: T1, treatment 1; T2, treatment 2 (baseline); HR, hazard ration; CI, confidence interval; NE, not estimable; RPSFT, rank preserving structural failure time model; NR, not reported.
Treatment codes: 1, everolimus; 2, cabozantinib; 3, nivolumab; 4, best supportive care/placebo; 5, axitinib; 6, sorafenib; 7, lenvatinib with everolimus

Table 9. Study data: health-related quality of life (not meta-analysed)

**Study**	**T1**	**T2**	**Study analysis details**	**FKSI scales**	**EuroQol scales**	**EORTC QLQ-C30**
AXIS[16]	5	6	End of treatment MD (95% CI)	DRS: MD 0.12 (-0.45 to 0.69) p = 0.68; FKSI-15: MD 0.35 (-0.63 to 1.34) p = 0.48	5D Index: MD 0.02 (-0.01 to 0.05) p = 0.19 VAS: -0.53 (-2.77 to 1.72) p = 0.65	NR

For peer review only - http://bmjopen.bmj.com/site/about/guidelines.xhtml

SUPPLEMENTARY FILE

Targeted therapies for previously treated advanced or metastatic renal cell carcinoma: systematic review and network meta-analysis

CheckMate 025[2]	3	1	Median change (range) at week 104	DRS:<br>niv -2 (-1 to 16)<br>evo 2 (-7 to 15)	NR	NR
HOPE 205	7	1	NA	NR	NR	NR
METEOR	2	1	NA	NR	NR	NR
RECORD-1[17]	1	4	Time to deterioration HR (95% CI); results favour placebo	DRS: HR 0.82, 95% CI 0.75 to 0.92, p = 0.001	NR	Global health status HR 0.85 (0.75 to 0.96) p = 0.006<br>Physical functioning HR 0.84 (0.75 to 0.94) p = 0.001
Abbreviations: FKSI-DRS = Functional Assessment of Cancer Therapy (FACT) Kidney Cancer Symptom Index; DRS = Disease-related Symptoms subscale of the FKSI-15; EQ-5D = European Quality of Life self-report questionnaire; VAS = visual analogue scale; EORTC QLQ = European Organization for the Research and Treatment of Cancer Quality of Life Questionnaire; MD, mean difference; CI, confidence interval; HR, hazard ratio; NA, not applicable; NR, not reported						

For peer review only

SUPPLEMENTARY FILE

Targeted therapies for previously treated advanced or metastatic renal cell carcinoma: systematic review and network meta-analysis

WINBUGS CODE

CODE 1: Fixed effect log hazard ratio NMA for 2-arm studies (overall survival primary and progression-free survival primary)

```
model{
#Model for log-hazard ratios
for(i in 1:ndp){
            prec[i]<- 1/(se[i]*se[i])
                  lhr[i]~dnorm(md[i],prec[i])

#Fixed effect model for log hazard ratios
                  md[i] <- d[t[i]] - d[b[i]]

#Deviance residuals for data i
   dev[i] <- (lhr[i] - md[i])*(lhr[i] - md[i])/(se[i]*se[i])
         }
         resdev <- sum(dev[])

#Give priors for log hazard ratios
         d[1]<-0
         for (k in 2:nt){
         d[k] ~ dnorm(0,.001)
         }

#Rank the treatment effects (with 1=best) & record the best treatment
for(k in 1:nt){
         rk[k]<- rank(d[],k)
         best[k]<-equals(rk[k],1)
         }

#All pairwise log hazard ratios and hazard ratios
for (c in 1:nt-1){
         for (k in (c+1):nt){
                  lhzr[c,k] <- d[k] - d[c]
                  HR[c,k] <- exp(lhzr[c,k])
                  }
         }
}
```

CODE 2: Fixed effect odds ratio NMA (objective response rate analysis)

```
model{

for(i in 1:ns){

         delta[i,t[i,1]]<-0
         mu[i] ~ dnorm(0,.0001)

         for (k in 1:na[i]) {
                  r[i,t[i,k]] ~ dbin(p[i,t[i,k]],n[i,t[i,k]])
```


For peer review only - http://bmjopen.bmj.com/site/about/guidelines.xhtml

SUPPLEMENTARY FILE

Targeted therapies for previously treated advanced or metastatic renal cell carcinoma: systematic review and network meta-analysis

```
                logit(p[i,t[i,k]])<-mu[i] + delta[i,t[i,k]]

        rhat[i,t[i,k]]<- p[i,t[i,k]] * n[i,t[i,k]]

        resdev[i,k]<- 2 * (r[i,t[i,k]] * (log(r[i,t[i,k]]) - log(rhat[i,t[i,k]])) + (n[i,t[i,k]] - r[i,t[i,k]]) * (log(n[i,t[i,k]] - r[i,t[i,k]]) - log(n[i,t[i,k]] - rhat[i,t[i,k]])))
        }
        sumdev[i]<-sum(resdev[i,1:na[i]])

for (k in 2:na[i]) {
        delta[i,t[i,k]] <- d[t[i,k]] - d[t[i,1]]        # trial-specific LOR
        }
}

sumdevtot<- sum(sumdev[])

d[1]<-0
for (k in 2:nt){
        d[k] ~ dnorm(0,.0001)
        }

for (i in 1:ns) {
        mu1[i] <- mu[i] * equals(t[i,1],1)
        }

for (k in 1:nt) {
        logit(T[k])<- sum(mu1[])/nb +d[k]
        }

for (k in 1:nt) {
        rk[k]<-nt - rank(T[],k)
        best[k]<-equals(rk[k],1)
        }

for (c in 1:(nt-1)) {
        for (k in (c+1):nt) {
                lor[c,k] <- (d[k] - d[c])
                or[c,k]<-exp(lor[c,k])
                        }
                }
}
```

CODE 3: Fixed effect log hazard ratio NMA to combine 2-arm and multi-arm studies (overall survival sensitivity and progression-free survival sensitivity)

```
model{

# Priors

    #On tx effect mean
    beta[1] < -0
    for (tt in 2:nt){
    beta[tt]~dnorm(0,1.0E-6)
    }

    #On individual study baseline effect
```


For peer review only - http://bmjopen.bmj.com/site/about/guidelines.xhtml

SUPPLEMENTARY FILE

Targeted therapies for previously treated advanced or metastatic renal cell carcinoma: systematic review and network meta-analysis

```
    for(ss in 1:ns){
    alpha[ss] ~ dnorm(0,1.0E-6)
    }

# Fit data
    for(ii in 1:ndp){
    mu[ii] < - alpha[t[ii]]*multi[ii] + beta[tx[ii]] - beta[b[ii]]
    prec[ii] < - 1/pow(se[ii],2)
    m[ii] ~ dnorm(mu[ii],prec[ii])
    }

# Calculate HRs
    for (hh in 1:nt) {
    hr[hh] < -exp(beta[hh])
    }

# Rank
    for (ll in 1:nt) {
                        rk[ll]<-rank(beta[],ll)
      best[ll] < - equals(rk[ll],1)
       }
    }
}
```

RESULTS

Table 10. Results of the RCT network meta-analyses for objective response rate, with 0.5 correction of 0 values

	Best supportive care	Lenvatinib+ everolimus	Nivolumab	Cabozantinib	Everolimus
Everolimus	0.24 (0.00 to 1.39)	91190 (9.30 to 34400)	6.23 (3.78 to 10.01)	6.61 (3.27 to 12.55)	-
Cabozantinib	0.04 (0.00 to 0.24)	14500 (1.36 to 5629)	1.06 (0.41 to 2.19)	-	0.15 (0.08 to 0.31)
Nivolumab	0.00 (0.00 to 0.24)	7.73 (1.46 to 5652)	-	0.94 (0.46 to 2.41)	0.16 (0.10 to 0.26)
Lenvatinib + everolimus	0.01 (0.00 to 0.04)	-	0.13 (0.00 to 0.68)	0.00 (0.00 to 0.74)	0.00 (0.00 to 0.11)
BSC	-	193 (24.99 to 4494382)	657000 (4.15 to 9580)	23.53 (4.10 to 30312)	4.02 (0.72 to 4826)

Abbreviations: BSC, best supportive care; OS, overall survival; PFS, progression-free survival
Results are odds ratios with 95% credible interval; odds ratios > 1 favour the treatment along the top row.

For peer review only - http://bmjopen.bmj.com/site/about/guidelines.xhtml

SUPPLEMENTARY FILE

Targeted therapies for previously treated advanced or metastatic renal cell carcinoma: systematic review and network meta-analysis

REFERENCES

1. Motzer RJ, Escudier B, Tomczak P, Hutson TE, Michaelson MD, Negrier S, et al. Axitinib versus sorafenib as second-line treatment for advanced renal cell carcinoma: overall survival analysis and updated results from a randomised phase 3 trial, 2013. Available from: http://onlinelibrary.wiley.com/o/cochrane/clcentral/articles/055/CN-00864055/frame.html. Date accessed.
2. Motzer RJ, Escudier B, McDermott DF, George S, Hammers HJ, Srinivas S, et al. Nivolumab versus Everolimus in Advanced Renal-Cell Carcinoma, 2015. Available from: http://onlinelibrary.wiley.com/o/cochrane/clcentral/articles/537/CN-01108537/frame.html. Date accessed.
3. Hutson TE, Dutcus CE, Ren M, Baig MA, Fishman MN. Subgroup analyses and updated overall survival from the phase II trial of lenvatinib (LEN), everolimus (EVE), and LEN+EVE in metastatic renal cell carcinoma (mRCC). Journal of Clinical Oncology Conference 2016; 34.
4. Choueiri T, Escudier B, Powles T, Tannir N, Mainwaring P, Rin iB. Cabozantinib versus everolimus in advanced renal cell carcinoma (METEOR): final results from a randomised, open-label, phase 3 trial. The Lancet Oncology 2016; 17: 917-27.
5. Korhonen P, Zuber E, Branson M, al. e. Correcting overall survival for the impact of crossover via a rank-preserving structural failure time (RPSFT) model in the RECORD-1 trial of everolimus in metastatic renal-cell carcinoma. J Biopharm Stat 2012; 22: 1258-71.
6. Guida A, Albiges L, Derosa L, Loriot Y, Massard C, Fizazi K, et al. Everolimus Versus Axitinib as Second-line Therapy in Metastatic Renal Cell Carcinoma: Experience From Institut Gustave Roussy. Clinical Genitourinary Cancer 2017; 15: e1081-e8.
7. Heng DY, Signorovitch J, Swallow E, Li N, Zhong Y, Qin P, et al. Comparative Effectiveness of Second-Line Targeted Therapies for Metastatic Renal Cell Carcinoma: A Systematic Review and Meta-Analysis of Real-World Observational Studies. PLoS ONE [Electronic Resource] 2014; 9: e114264.
8. Iacovelli R, Santini D, Rizzo M, Felici A, Santoni M, Verzoni E, et al. Bone metastases affect prognosis but not effectiveness of third-line targeted therapies in patients with metastatic renal cell carcinoma. Can Urol Assoc J 2015; 9: 263-7.
9. Arranz Arija J, Perez-Valderrama B, Rodriguez Sanchez A, Puertas Alvarez J, Pinto Marin A, Maximiano Alonso C, et al. SPAZO2 (SOGUG): Comparative effectiveness of everolimus (Ev) vs axitinib (Ax) as second-line after first-line pazopanib (1stPz) in metastatic renal carcinoma (mRC). Annals of Oncology 2017; 28 (Supplement 5): v313.
10. Vogelzang NJ, Pal SK, Signorovitch JE, Reichmann WM, Li N, Yang C, et al. Comparative effectiveness of everolimus and axitinib as second targeted therapies for metastatic renal cell carcinoma in the US: A retrospective chart review. Current Medical Research and Opinion 2016; 32: 741-7.
11. Wong MK, Yang H, Signorovitch JE, Wang X, Liu Z, Liu NS, et al. Comparative outcomes of everolimus, temsirolimus and sorafenib as second targeted therapies for metastatic renal cell carcinoma: A US medical record review. Current Medical Research and Opinion 2014; 30: 537-45.
12. Motzer RJ, Hutson TE, Ren M, Dutcus C, Larkin J. Independent assessment of lenvatinib plus everolimus in patients with metastatic renal cell carcinoma, 2016. Available from: http://onlinelibrary.wiley.com/o/cochrane/clcentral/articles/453/CN-01168453/frame.html. Date accessed.
13. Motzer RJ, Escudier B, Oudard S, Hutson TE, Porta C, Bracarda S, et al. Phase 3 trial of everolimus for metastatic renal cell carcinoma : final results and analysis of prognostic factors, 2010. Available from: http://onlinelibrary.wiley.com/o/cochrane/clcentral/articles/159/CN-00762159/frame.html. Date accessed.
14. Rini BI, Escudier B, Tomczak P, Kaprin A, Szczylik C, Hutson TE, et al. Comparative effectiveness of axitinib versus sorafenib in advanced renal cell carcinoma (AXIS): a randomised phase 3 trial, 2011. Available from: http://onlinelibrary.wiley.com/o/cochrane/clcentral/articles/402/CN-00804402/frame.html. Date accessed.
15. Motzer RJ, Hutson TE, Glen H, Michaelson MD, Molina A, Eisen T, et al. Lenvatinib, everolimus, and the combination in patients with metastatic renal cell carcinoma: a randomised, phase 2,open-label, multicentre trial. Lancet Oncology 2015; 16: 1473-82.

For peer review only - http://bmjopen.bmj.com/site/about/guidelines.xhtml

SUPPLEMENTARY FILE

Targeted therapies for previously treated advanced or metastatic renal cell carcinoma: systematic review and network meta-analysis

16. Cella D, Escudier B, Rini B, Chen C, Bhattacharyya H, Tarazi J, et al. Patient-reported outcomes for axitinib vs sorafenib in metastatic renal cell carcinoma: phase III (AXIS) trial. British Journal of Cancer 2013; 108: 1571-8.
17. Beaumont JL, Butt Z, Baladi J, Motzer RJ, Haas T, Hollaender N, et al. Patient-reported outcomes in a phase iii study of everolimus versus placebo in patients with metastatic carcinoma of the kidney that has progressed on vascular endothelial growth factor receptor tyrosine kinase inhibitor therapy, 2011. Available from: http://onlinelibrary.wiley.com/o/cochrane/clcentral/articles/925/CN-00800925/frame.html

For peer review only

For peer review only - http://bmjopen.bmj.com/site/about/guidelines.xhtml

PRISMA 2009 Checklist

Section/topic	#	Checklist item	Reported on page #
**TITLE**			
Title	1	Identify the report as a systematic review, meta-analysis, or both.	1
**ABSTRACT**			
Structured summary	2	Provide a structured summary including, as applicable: background; objectives; data sources; study eligibility criteria, participants, and interventions; study appraisal and synthesis methods; results; limitations; conclusions and implications of key findings; systematic review registration number.	2-4
**INTRODUCTION**			
Rationale	3	Describe the rationale for the review in the context of what is already known.	7
Objectives	4	Provide an explicit statement of questions being addressed with reference to participants, interventions, comparisons, outcomes, and study design (PICOS).	7
**METHODS**			
Protocol and registration	5	Indicate if a review protocol exists, if and where it can be accessed (e.g., Web address), and, if available, provide registration information including registration number.	3
Eligibility criteria	6	Specify study characteristics (e.g., PICOS, length of follow-up) and report characteristics (e.g., years considered, language, publication status) used as criteria for eligibility, giving rationale.	7-9
Information sources	7	Describe all information sources (e.g., databases with dates of coverage, contact with study authors to identify additional studies) in the search and date last searched.	9-10
Search	8	Present full electronic search strategy for at least one database, including any limits used, such that it could be repeated.	Suppl. 2-3, Table 1
Study selection	9	State the process for selecting studies (i.e., screening, eligibility, included in systematic review, and, if applicable, included in the meta-analysis).	9, 11
Data collection process	10	Describe method of data extraction from reports (e.g., piloted forms, independently, in duplicate) and any processes for obtaining and confirming data from investigators.	9-10
Data items	11	List and define all variables for which data were sought (e.g., PICOS, funding sources) and any assumptions and simplifications made.	9-10, suppl. 4-6, Table 2
Risk of bias in individual studies	12	Describe methods used for assessing risk of bias of individual studies (including specification of whether this was done at the study or outcome level), and how this information is to be used in any data synthesis.	10
Summary measures	13	State the principal summary measures (e.g., risk ratio, difference in means).	11
Synthesis of results	14	Describe the methods of handling data and combining results of studies, if done, including measures of consistency (e.g., I^2) for each meta-analysis.	10-11

For peer review only - http://bmjopen.bmj.com/site/about/guidelines.xhtml

PRISMA 2009 Checklist

Section/topic	#	Checklist item	Reported on page #
Risk of bias across studies	15	Specify any assessment of risk of bias that may affect the cumulative evidence (e.g., publication bias, selective reporting within studies).	10
Additional analyses	16	Describe methods of additional analyses (e.g., sensitivity or subgroup analyses, meta-regression), if done, indicating which were pre-specified.	11
**RESULTS**			
Study selection	17	Give numbers of studies screened, assessed for eligibility, and included in the review, with reasons for exclusions at each stage, ideally with a flow diagram.	Fig. 1
Study characteristics	18	For each study, present characteristics for which data were extracted (e.g., study size, PICOS, follow-up period) and provide the citations.	Table 1
Risk of bias within studies	19	Present data on risk of bias of each study and, if available, any outcome level assessment (see item 12).	16-17, suppl. 7-8, Table 3-4
Results of individual studies	20	For all outcomes considered (benefits or harms), present, for each study: (a) simple summary data for each intervention group (b) effect estimates and confidence intervals, ideally with a forest plot.	Suppl. 9-11, Table 5-9
Synthesis of results	21	Present results of each meta-analysis done, including confidence intervals and measures of consistency.	17-19, Table 2, Suppl. 14, Table 10
Risk of bias across studies	22	Present results of any assessment of risk of bias across studies (see Item 15).	4, 16-18
Additional analysis	23	Give results of additional analyses, if done (e.g., sensitivity or subgroup analyses, meta-regression [see Item 16]).	17-18, Table 2
**DISCUSSION**			
Summary of evidence	24	Summarize the main findings including the strength of evidence for each main outcome; consider their relevance to key groups (e.g., healthcare providers, users, and policy makers).	20, 23
Limitations	25	Discuss limitations at study and outcome level (e.g., risk of bias), and at review-level (e.g., incomplete retrieval of identified research, reporting bias).	21-22
Conclusions	26	Provide a general interpretation of the results in the context of other evidence, and implications for future research.	22-23
**FUNDING**			
Funding	27	Describe sources of funding for the systematic review and other support (e.g., supply of data); role of funders for the systematic review.	25

For peer review only

PRISMA 2009 Checklist

From: Moher D, Liberati A, Tetzlaff J, Altman DG, The PRISMA Group (2009). Preferred Reporting Items for Systematic Reviews and Meta-Analyses: The PRISMA Statement. PLoS Med 6(7): e1000097. doi:10.1371/journal.pmed1000097

For more information, visit: **www.prisma-statement.org**.

For peer review only

Reporting checklist for meta-analysis of observational studies.

Based on the MOOSE guidelines.

Instructions to authors

Complete this checklist by entering the page numbers from your manuscript where readers will find each of the items listed below.

Your article may not currently address all the items on the checklist. Please modify your text to include the missing information. If you are certain that an item does not apply, please write "n/a" and provide a short explanation.

Upload your completed checklist as an extra file when you submit to a journal.

In your methods section, say that you used the MOOSE reporting guidelines, and cite them as:

Stroup DF, Berlin JA, Morton SC, Olkin I, Williamson GD, Rennie D, Moher D, Becker BJ, Sipe TA, Thacker SB. Meta-analysis of observational studies in epidemiology: a proposal for reporting. Meta-analysis Of Observational Studies in Epidemiology (MOOSE) group. JAMA. 2000; 283(15):2008-2012.

	Reporting Item	Page Number
#1	Identify the study as a meta-analysis of observational research	2
#2	Provide a structured summary including, as applicable: background; objectives; data sources; study eligibility criteria, participants, and interventions; study appraisal and synthesis methods; results; limitations; conclusions and implications of key findings; systematic review registration number (From PRISMA checklist)	2-4
#3a	Problem definition	7
#3b	Hypothesis statement	n/a
#3c	Description of study outcomes	8
#3d	Type of exposure or intervention used	8
#3e	Type of study designs used	8

For peer review only

	#3f	Study population	8
Search strategy	#4a	Qualifications of searchers (eg, librarians and investigators)	24
	#4b	Search strategy, including time period included in the synthesis and keywords	9-10, Suppl. 2-3, Table 1
	#4c	Effort to include all available studies, including contact with authors	10
	#4d	Databases and registries searched	9
	#4e	Search software used, name and version, including special features used (eg, explosion)	Suppl. 2, Table 1
	#4f	Use of hand searching (eg, reference lists of obtained articles)	9
	#4g	List of citations located and those excluded, including justification	n/a, word count restriction
	#4h	Method of addressing articles published in languages other than English	9
	#4i	Method of handling abstracts and unpublished studies	9
	#4j	Description of any contact with authors	10
	#5a	Description of relevance or appropriateness of studies gathered for assessing the hypothesis to be tested	12-17
	#5b	Rationale for the selection and coding of data (eg, sound clinical principles or convenience)	9-10
	#5c	Documentation of how data were classified and coded (eg, multiple raters, blinding, and interrater reliability)	9-10
	#5d	Assessment of confounding (eg, comparability of cases and controls in studies where appropriate)	10
	#5e	Assessment of study quality, including blinding of quality assessors; stratification or regression on possible predictors of study results	10
	#5f	Assessment of heterogeneity	10
	#5g	Description of statistical methods (eg, complete description of fixed or random effects models, justification of whether the chosen	10-11

For peer review only

	models account for predictors of study results, dose-response models, or cumulative meta-analysis) in sufficient detail to be replicated	
#5h	Provision of appropriate tables and graphics	Figure 1,2, p13-14, Table 1, p18 Table 2, Suppl. all Tables
#6a	Graphic summarizing individual study estimates and overall estimate	18, Table 2, suppl. 9-11, Table 5-9
#6b	Table giving descriptive information for each study included	13-14, Table 1
#6c	Results of sensitivity testing (eg, subgroup analysis)	17-18, Table 2
#6d	Indication of statistical uncertainty of findings	18, Table 2
#7a	Quantitative assessment of bias (eg. publication bias)	10, 17-18
#7b	Justification for exclusion (eg, exclusion of non–English-language citations)	9
#7c	Assessment of quality of included studies	16-17, suppl. 7-8, Table 3-4
#8a	Consideration of alternative explanations for observed results	21
#8b	Generalization of the conclusions (ie, appropriate for the data presented and within the domain of the literature review)	22-23
#8c	Guidelines for future research	23
#8d	Disclosure of funding source	25

Reproduced with permission from JAMA. 2000. 283(15):2008-2012. Copyright © 2000 American Medical Association. All rights reserved.This checklist can be completed online using https://www.goodreports.org/, a tool made by the EQUATOR Network in collaboration with Penelope.ai

For peer review only

BMJ Open

TARGETED THERAPIES FOR PREVIOUSLY TREATED ADVANCED OR METASTATIC RENAL CELL CARCINOMA: SYSTEMATIC REVIEW AND NETWORK META-ANALYSIS

Journal:	*BMJ Open*
Manuscript ID	bmjopen-2018-024691.R2
Article Type:	Research
Date Submitted by the Author:	14-Jan-2019
Complete List of Authors:	Karner, Charlotta; British Medical Journal Technology Assessment Group (BMJ-TAG) BMA House Tavistock Square London, UK WC1H 9JR<br>Kew, Kayleigh; British Medical Journal Technology Assessment Group (BMJ-TAG) BMA House Tavistock Square London, UK WC1H 9JR<br>Wakefield, Victoria; British Medical Journal Technology Assessment Group (BMJ-TAG) BMA House Tavistock Square London, UK WC1H 9JR<br>Masento, Natalie; British Medical Journal Technology Assessment Group (BMJ-TAG) BMA House Tavistock Square London, UK WC1H 9JR<br>Edwards, S; British Medical Journal Technology Assessment Group (BMJ-TAG) BMA House Tavistock Square London, UK WC1H 9JR
**Primary Subject Heading**:	Oncology
Secondary Subject Heading:	Renal medicine, Pharmacology and therapeutics, Evidence based practice, Oncology
Keywords:	metastatic renal cell carcinoma, network meta-analysis, overall survival, progression-free survival, systematic reveiw

SCHOLARONE™
Manuscripts

TARGETED THERAPIES FOR PREVIOUSLY TREATED ADVANCED OR METASTATIC RENAL CELL CARCINOMA: SYSTEMATIC REVIEW AND NETWORK META-ANALYSIS

Charlotta Karner PhD, Kayleigh Kew MA (Cantab), Victoria Wakefield MBChB, Natalie Masento PhD, Steven J. Edwards DPhil*

Affiliations: BMJ Technology Assessment Group (BMJ-TAG), BMA House, Tavistock Square, London, WC1H 9JP, UK (all authors)

*corresponding author: sedwards@bmj.com

Word count: 4312 (excluding title page, abstract, article summary, references, figures and tables)

Topic area: Oncology

Key words: metastatic renal cell carcinoma, network meta-analysis, overall survival, progression-free survival, systematic review

Checklists completed: PRISMA 2009, MOOSE

For peer review only - http://bmjopen.bmj.com/site/about/guidelines.xhtml

ABSTRACT

Objective

To compare the effectiveness and safety of treatments for advanced or metastatic renal cell carcinoma (amRCC) after treatment with vascular endothelial growth factor (VEGF)-targeted treatment.

Design

Systematic review and network meta-analysis of randomised controlled trials (RCTs) and comparative observational studies. MEDLINE, EMBASE, and Cochrane Library were searched up to January 2018.

Participants

People with amRCC requiring treatment after VEGF-targeted treatment.

Interventions

Axitinib, cabozantinib, everolimus, lenvatinib with everolimus, nivolumab, sorafenib and best supportive care (BSC).

Outcomes

Primary outcomes were overall survival (OS) and progression-free survival (PFS); secondary outcomes were objective response rate (ORR), adverse events, and health-related quality of life (HRQoL).

Results

Twelve studies were included (n = 5,144): five RCTs and seven observational studies. Lenvatinib with everolimus significantly increased OS and PFS over everolimus (HR 0.61, 95% Credible Interval [95%CrI]: 0.36 to 0.96 and 0.47, 95%CrI: 0.26 to 0.77, respectively) as did

For peer review only

cabozantinib (HR 0.66, 95%CrI: 0.53 to 0.82 and 0.51, 95%CrI: 0.41 to 0.63, respectively). This remained the case when observational evidence was included. Nivolumab also significantly improved OS versus everolimus (HR 0.74, 95%CrI: 0.57 to 0.93). OS sensitivity analysis, including observational studies, indicates everolimus being more effective than axitinib and sorafenib. However, inconsistency was identified in the OS sensitivity analysis. PFS sensitivity analysis suggests axitinib is more effective than everolimus, which may be more effective than sorafenib. The results for ORR supported the OS and PFS analyses. Nivolumab is associated with fewer grade 3 or 4 adverse events than lenvatinib with everolimus or cabozantinib. HRQoL could not be analysed due to differences in tools used.

Conclusions

Lenvatinib with everolimus, cabozantinib and nivolumab are effective in prolonging survival for people with amRCC subsequent to VEGF-targeted treatment, but there is considerable uncertainty about how they compare to each other and how much better they are than axitinib and sorafenib.

Protocol registration: PROSPERO CRD42017071540

Data sharing statement: search strategies, data extraction form, risk of bias summaries, data inputs, NMA code and results tables are provided in a supplementary file.

ARTICLE SUMMARY

Strengths and limitations of this study

For peer review only

For peer review only - http://bmjopen.bmj.com/site/about/guidelines.xhtml

- This review is highly relevant and timely as it includes all recently approved treatments and focuses on the effectiveness of these treatments when used after first line VEGF-targeted tyrosine kinase inhibitor (TKI) treatment, as recommended in European clinical guidelines.
- The review focuses on high quality RCT evidence, but inclusion of comparative observational evidence in sensitivity analyses enabled estimates for axitinib and sorafenib, which otherwise could not be connected in the network.
- The reliability of the results of this review is hampered by trial design limitations of some of the included studies: the proportional hazards assumption did not hold for PFS in the one trial including nivolumab, RCT data for axitinib and sorafenib were limited to a subgroup analysis conducted in one study which could only be compared to the other treatments by including observational studies, and the trial assessing lenvatinib with everolimus is a small phase II trial with an increased risk of a false positive result and of over estimating the effect size due to some differences in baseline characteristics and relatively low significance level (alpha 0.15).
- There were also some differences between the trials in the network in terms of baseline characteristics, number and type of prior VEGF targeted treatments, and trial blinding, but there were too few studies to explore the potentially treatment modifying effects of these differences.

BACKGROUND

For peer review only

Kidney cancers are among the most common cancers in Europe (age-standardised rates estimated at 17.2/100,000 males and 8.1/100,000 females)[1] and renal cell carcinoma (RCC) makes up 80–90% of new cases. RCC occurs most commonly in men over 60 years, and smoking, obesity, hypertension, germline mutations and advanced kidney disease are established risk factors.[2] . RCC is often asymptomatic until later stages, so most people are diagnosed with advanced or metastatic disease (amRCC); five-year survival of amRCC is less than 10% and the goal of treatment is to slow progression and treat symptoms.[2]

Targeted treatments are designed to interrupt the biological pathways needed for the cancer to grow and spread. Since 2006, eight targeted treatments have been approved by the European Medicines Agency (EMA) for the treatment of amRCC,[3-10] falling within three classes: mammalian target of rapamycin inhibitors (mTORis; everolimus[5]), tyrosine kinase inhibitors (TKIs; sunitinib,[3] pazopanib,[7] axitinib,[6] cabozantinib,[8] lenvatinib[9] [in combination with the mTORi everolimus] and sorafenib[4]), and PD-1 monoclonal antibodies (nivolumab[10]). The mechanism of action of each treatment affects tolerability and has implications for treatment choice based on patient characteristics.[11]

The emergence of targeted treatments has changed the RCC treatment pathway substantially and targeted treatments have virtually replaced the use of cytokines in many European health systems.[12] As a result, published studies assessing second-line targeted agents in populations who received first-line cytokines, or indeed adjusted indirect comparisons combining studies that enrolled those having received prior cytokines, have limited applicability to current practice. Sunitinib and pazopanib (VEGFRs) are the only recommended first-line treatments in the latest RCC European Society for Medical Oncology (ESMO) clinical practice guidelines.[12]

For peer review only

For peer review only - http://bmjopen.bmj.com/site/about/guidelines.xhtml

ESMO recommends axitinib, cabozantinib, sorafenib, everolimus, nivolumab, and lenvatinib with everolimus as treatment options from second line.[12]

Second-line practice patterns are not well established, partly because some treatments have only relatively recently been approved by the EMA.[13-15] Randomised controlled trials (RCTs), cohorts and patient registry data are emerging but head-to-head comparisons remain limited. Given the high cost of RCTs, and the number of treatments available for use at second line, it is unlikely that every treatment will ever be compared to every other treatment available. As such, adjusted indirect treatment comparisons are required to provide estimates beyond trial comparators to help establish an evidence-based treatment sequence for amRCC. Before cabozantinib, nivolumab and lenvatinib with everolimus were approved, network meta analyses (NMAs) of RCTs or good quality observational cohorts favoured axitinib and everolimus over sorafenib, though primarily within populations who had received prior cytokines.[16-19] Two NMAs of RCTs comparing more recently approved drugs indicate that lenvatinib with everolimus or cabozantinib are likely to be the most effective option to extend overall survival (OS) and progression-free survival (PFS) in amRCC. However, neither study included all the relevant treatments and both NMAs combine evidence for people who had either received prior cytokines or VEGF-targeted agents, reflecting an outdated pathway and unreliable results given that type of prior treatment is a potential treatment effect modifier.[20]

This systematic review is the first to include randomised and observational evidence for all recently-approved targeted treatments for amRCC, focusing specifically on the relevant population who have previously received a VEGF-targeted treatment. By doing so, the review aims to provide a full and clinically relevant assessment of treatment safety and clinical

For peer review only - http://bmjopen.bmj.com/site/about/guidelines.xhtml

effectiveness, focusing on outcomes that are the most important to patients (OS, PFS, overall response rate (ORR), quality of life, and adverse events).

OBJECTIVE

To compare the safety and clinical effectiveness of targeted treatments for amRCC previously treated with VEGF-targeted therapy.

METHODS

Methods for the review are reported in more detail in the published protocol (CRD42017071540) and were based on the principles published by the National Health Service Centre for Reviews and Dissemination.[21] The review reported here is an update and extension of a project commissioned by the UK National Institute for Health Research (NIHR), registered as CRD42016042384. This review was reregistered and updated to make the results applicable outside the UK and to include treatments that have received European marketing authorisation subsequent to publication of the first iteration of the review.

Patient and public involvement

Patients were not directly involved in the development of this review update but the original review was based on a scope produced by the National Institute for Health and Care Excellence (NICE) within which patients and patient groups were registered stakeholders.

Eligibility criteria

Study design

For peer review only

For peer review only - http://bmjopen.bmj.com/site/about/guidelines.xhtml

RCTs formed the basis of the primary analyses for all outcomes. As per the published protocol, comparative observational studies were included in sensitivity analyses for OS and PFS to provide a connected network for all interventions of interest. Preclinical studies, animal studies, narrative reviews, editorials, opinions and case reports were not eligible.

Population

Adults (18+ years) with a diagnosis of amRCC who had received previous treatment with a VEGF-targeted treatment.

Interventions

Interventions of interest were axitinib, cabozantinib, everolimus, lenvatinib with everolimus, nivolumab and sorafenib. Studies were included if they compared any of the listed interventions with each other, placebo or best supportive care (BSC). For the purposes of this review, placebo was assumed to be the equivalent of BSC. Studies comparing an intervention of interest with another treatment were only included if there were insufficient direct comparisons to provide a connected network that included all treatments of interest.

Outcomes

The primary outcomes were OS and PFS. Secondary outcomes were predefined as objective response rate (ORR), adverse events of Grade 3 and above (as defined by the Common Terminology Criteria for Adverse Events), and health-related quality of life (HRQoL).

Studies were excluded if none of the outcomes of interest were reported. Comparative observational studies were only included if they reported OS or PFS in a way that could be

For peer review only - http://bmjopen.bmj.com/site/about/guidelines.xhtml

incorporated into the NMA (i.e. as a hazard ratio [HR] or where a HR could be estimated from a Kaplan-Meier curve with the number of people at risk).

Search and selection process

Electronic searches for the original project were run in January 2016 (for RCTs; MEDLINE, EMBASE and CENTRAL) and June 2016 (observational studies; MEDLINE and EMBASE), and subsequently extended to cover a new intervention (lenvatinib with everolimus) and updated to January 2018. Manual searches of conference proceedings and bibliographies of included studies and systematic review were also updated to January 2018. Searches combined terms for the interventions of interest with condition terms for RCC and the relevant design filter (RCT or observational; example strategy provided in the Supplementary file [Table 1]). No date or language restrictions were applied. Searches for observational evidence were limited to interventions required to connect the network of treatments.

Unpublished and ongoing studies were identified by contacting experts in the field and searching ClinicalTrials.gov and the EU Clinical Trials Register.

Two reviewers screened all titles and abstracts independently. Full texts were retrieved and reviewed for records identified as potentially relevant by one or both reviewers. Discrepancies were resolved by consensus or by involving a third reviewer.

Data extraction and quality assessment

Data extraction was carried out independently by two reviewers and cross-checked for accuracy; as with study selection, discrepancies were resolved by discussion or by involving a third reviewer. A standard data extraction form was piloted and used to capture information

For peer review only

For peer review only - http://bmjopen.bmj.com/site/about/guidelines.xhtml

about study conduct, population, interventions, outcomes and risk of bias from each study, including the information source where more than one was available for a given study (template available in the Supplementary file [Table 2] together with extracted datasets for all outcomes). Where there were incomplete information study authors were contacted to gain further details.

Methodological quality was assessed independently by two reviewers using the Cochrane Risk of Bias tool for RCTs[22] and the ROBINS-I for comparative observational studies.[23] Where appropriate, risk of bias was assessed separately for each outcome within a study. Disagreements were resolved by consensus or by involving a third reviewer. The likely direction and magnitude of bias across the evidence as a whole was considered during interpretation of the results.

Data synthesis

Baseline characteristics of the included studies were compared to assess similarity of the study populations before combining results in an NMA. Fixed effects and random effects models were explored. However, as typically only one trial informed each pair-wise comparison and hence there were little data to inform the between trial heterogeneity, a pragmatic decision was made to use the fixed effects model for all outcomes. Statistical heterogeneity was assessed using the I^2 statistic for pairwise comparisons and deviance information criteria (DIC) for NMA. Inconsistency between direct and indirect effect estimates was assessed in closed loops in the network. Implications of observed clinical and statistical heterogeneity and inconsistency are described in the results.

For peer review only - http://bmjopen.bmj.com/site/about/guidelines.xhtml

Where NMA was possible, it was conducted according to the guidance described in the NICE Decisions Support Unit's Technical Support Documents for Evidence Synthesis.[24] A Bayesian Markov Chain Monte Carlo (MCMC) approach was taken in WinBUGS version 1.4.3 software[25] (codes included in the supplementary file) implementing uninformed priors and a burn-in of 30,000 iterations. Everolimus was specified as the baseline treatment. Data from multi-arm studies were adjusted to account for correlations in relative treatment effects.[26] OS and PFS were analysed as HRs, and response as odds ratios (ORs) using participants as the unit of analysis; no formal analysis could be performed for adverse effects or HRQoL due to between-study variation in reporting. A 95% Credible Interval can be interpreted as a 95% probability that the parameter falls within this range. If a 95% CrI doesn't include one this can, therefore, be interpreted as a statistically significant result (at the 5% level of significance). Primary analyses were based on studies of low, unclear or moderate risk of bias. Sensitivity analyses were planned for OS and PFS including RCTs of high risk of bias and observational studies of serious risk of bias. Observational studies at critical risk of bias were excluded from all analyses.

RESULTS

Results of the searches

Results of the original and update search and selection process are shown in Figure 1.

The searches carried out in June 2016 led to the inclusion of 44 records relating to 12 studies. Five of these studies have been excluded from this review because of the update of the scope excluding sunitinib as it is not recommended at second line in the most up-to-date ESMO

For peer review only - http://bmjopen.bmj.com/site/about/guidelines.xhtml

guidance for RCC.[12] Five new studies, one RCT and four retrospective chart reviews, were identified in the update and extension searches (including terms for lenvatinib with everolimus) run in January 2018, making a total of 12 included studies.[13, 15, 19, 20, 27-34]

Included studies

Twelve studies (n = 5,144) met the inclusion criteria (Table 1): five RCTs (one double-blind[29] and four open-label[13, 15, 20, 28]); and seven observational studies[19, 27, 30-34] (retrospective cohort studies). Sample sizes varied from 101 (HOPE 205[15]) to 821 (CheckMate 025[35]) participants.

For peer review only - http://bmjopen.bmj.com/site/about/guidelines.xhtml

Table 1. Study characteristics

Study	Design	Location, funding	Prior treatments	Intervention	N	Type	Median age years	Male %	ECOG 0/1 %	Treatment duration (follow-up) months
AXIS[20]	PIII OL RCT	175 sites in 22 countries, Pfizer	1 prior systemic treatment (sunitinib, cytokine or other), prior sunitinib subgroup 54%	Axitinib	361	CC	61	73	99	8.2 (NR)
				Sorafenib	362		61	71	100	5.2 (NR)
CheckMate 025[35]	PIII OL RCT	146 sites in 24 countries, BMS	1 or 2 prior targeted treatments (TKI or other, no mTORi)	Nivolumab	410	CC	62	77	NR	5.5 (NR)
				Everolimus	411		62	74		3.7 (NR)
HOPE 205[15]	PII OL RCT	37 sites in Czech Republic, Poland, Spain, UK, US, Eisai	1 prior TKI, no prior mTORi	Lenvatinib+eve	51	CC	61	69	100	7.6 (NR)
				Everolimus	50		59	76	100	4.1 (NR)
METEOR[13]	PIII OL RCT	173 sites in 26 countries, Exelixis	1 or more prior TKIs; no prior mTORi	Cabozantinib	330	CC	63*	77	100	8.3 (18.7)
				Everolimus	328		62*	73	100	4.4 (18.8)
RECORD-1[29]	PIII DB RCT, Novartis	86 sites in Australia, Canada Europe, Japan, US, Novartis	1 or 2 prior TKIs; no prior mTORi	Everolimus	277	CC	61*	78	NR	4.6 (NR)
				BSC/placebo	139		60*	76		1.9 (NR)
Guida 2017[33]	Chart review	1 site in France, NR	1 prior targeted treatment (TKI or other)	Everolimus	81	92% CC	57	69	85	NR (33)
				Axitinib	45		54	78	82	NR (26)
Heng 2016[19]	Chart review	UK, Germany, France, Netherlands, Novartis	1 prior TKI (sunitinib or pazopanib)	Everolimus	115	NR	60.2	66.7	91.8% ≤ 2	NR (NR)
				Axitinib	96					NR (NR)
				Sorafenib	98					NR (NR)
Iacovelli 2015[27]	Chart review	23 sites in Italy, NR	2 prior targeted treatments (TKI or other)	Sorafenib	90	CC	63	74	81	NR (NR)
				Everolimus	143					NR (NR)
Lakomy 2017[34]	Chart	Czech national	1 prior targeted	Everolimus	520	94%	65	75	95	6.1 (NR)

For peer review only

For peer review only - http://bmjopen.bmj.com/site/about/guidelines.xhtml

	review	registry, **	treatment (TKI or other)	Sorafenib	240	CC	62	75	90	7.1 (NR)
SPAZO-2[32]	Chart review	50 sites in Spain, Novartis	1 prior TKI (pazopanib)	Everolimus	101	88% CC	66	64	NR	NR (28)
				Axitinib	88		63	68		
Vogelzang 2016[30]	Chart review	US, Novartis	1 prior TKI; no prior cytokines	Everolimus	325	85% CC	61*	70	80	NR (15*)
				Axitinib	127		60*	65	84	NR (13*)
Wong 2014[31]	Chart review	US, Novartis	1 prior TKI; no prior mTORi, cytokines, bevacizumab	Everolimus	233	91% CC	64	70	NR	NR (12.9)
				Sorafenib	123		66	72		NR (12.1)

Abbreviations: NR, not reported; RCT, randomised controlled trials; BSC, best supportive care; mRCC, metastatic renal cell carcinoma; amRCC, advanced or metastatic RCC; cc, clear cell variant; ncc, non-clear cell variant; TKI, tyrosine kinase inhibitor; mTORi, mammalian target of rapamaycin inhibitor. Notes: ECOG percentages that do not total 100 are due to missing data;
*mean values where median was not reported.
** Ministry of Health of the Czech Republic, Central European Institute of Technology, The Ministry of Education, Youth and Sports. RENIS registry part funded by Pfizer, Bayer and Novartis.

For peer review only

For peer review only - http://bmjopen.bmj.com/site/about/guidelines.xhtml

All studies recruited adults with amRCC who had received at least one prior VEGF-targeted treatment. AXIS[20] also included people who had not received prior anti-VEGF treatment, but OS and PFS data were available for the subset who had. In eight of the included studies people had only received one prior VEGF-targeted treatment;[14, 19, 30-34, 36] the remaining five studies allowed one or more prior treatments.[13, 27, 35, 37] Populations were predominantly male and Caucasian, and mean age was generally between 55 and 65 years. Where reported, most people had stage 3 or 4 clear-cell RCC and the majority had baseline ECOG performance status of 0 or 1. Baseline characteristics were generally well balanced between treatment groups within trials, with the exception of HOPE 205[14], in which there were some imbalances in baseline characteristics, which may favour lenvatinib with everolimus over everolimus.

Where dose was reported, it was started at the standard licensed dose and adjusted according to clinical judgement. Treatment was reported in the RCTs to be continued until disease progression, unacceptable toxicity or withdrawal of consent, except for METEOR[13] and CheckMate 025[35] in which people could be treated beyond progression. Median treatment duration in the five studies where it was reported varied from 1.9 months (placebo [BSC] group of RECORD-1[29]) to 8.3 months (cabozantinib group of METEOR[13]). Median length of follow-up ranged from 12.1 months to 23.6 months, but was only reported in four studies.

Most studies gave limited information regarding treatments received subsequent to the study drug. In RECORD-1,[29] 76% of people randomised to placebo received open-label everolimus at progression, but the confounding of OS was reduced by using crossover-adjusted data in the NMA. Treatment crossover was not reported to have occurred in any other studies.

Treatments compared in each of the studies are shown in Table 1 and Figure 2. Direct comparisons made by RCTs are shown by black lines, and the additional connections possible

For peer review only - http://bmjopen.bmj.com/site/about/guidelines.xhtml

by incorporating comparative observational studies are shown with green lines; axitinib and sorafenib did not connect to the other treatments using only RCT evidence. Nivolumab could not be connected in the PFS network because it was not appropriate to analyse CheckMate 025[38] data with a Cox proportional hazards model.

Risk of bias

The five RCTs[13, 15, 20, 29, 38] were of good methodological quality; all are at low risk of bias for random sequence generation and allocation concealment. RECORD-1[29] was the only blinded study so there is a risk of performance bias in the others. In general, OS and PFS are considered low risk of detection and reporting biases for all RCTs except for a high risk of PFS detection bias in CheckMate 025[35] because it was not assessed by an independent review committee. None of the outcomes in the RCTs were at high risk of attrition bias; all used appropriate censoring for the time-to-event analyses, although OS data from CheckMate 025[35] and METEOR[13] are immature. Other possible sources of bias pertain to group differences in the rate and type of subsequent treatments received, which were poorly reported in most trials. RECORD-1[29] was the only trial allowing cross-over for people in the placebo arm, although cross-over adjusted results were reported. Despite appropriate randomisation in HOPE 205[15], which is a small phase II trial, there were some imbalances in the baseline characteristics of the people in the trial, which may indicate a better prognosis for the lenvatinib with everolimus group compared with everolimus alone. In addition, alpha was set to 0.15, compared to the usual 0.05, and HOPE 205 is therefore of a higher risk of a false positive result and possibly of over estimating the effect size.

For peer review only

For peer review only - http://bmjopen.bmj.com/site/about/guidelines.xhtml

The observational studies included in the OS and PFS sensitivity analyses are at a higher risk of bias than the RCTs. Overall ROBINS-I ratings were at best moderate, for OS,[19, 30, 32] and serious risk of bias for PFS. One study was at critical risk of bias for both PFS and OS,[34] which was excluded from the sensitivity analyses. In all studies the potential for inadequate control for confounding was thought to increase the risk of bias. All studies reporting PFS also had an increased risk of bias for this outcome due to the lack of standardised measurement for assessing progression and that outcome assessors were aware of the interventions.

One of the observational studies was publicly funded,[34] two studies did not report their funding source[27, 33] and the remaining observational studies and all RCTs were sponsored by various pharmaceutical companies. Risk of bias assessments for all included studies are provided in the supplementary file (Tables 3 and 4).

Overall survival

Lenvatinib with everolimus, cabozantinib and nivolumab all showed statistically significant benefits over the baseline treatment, everolimus, in in the primary OS analysis (Table 2). Lenvatinib with everolimus had the highest probability (61%) of being the most effective treatment out of those compared in the primary analysis. These results were mirrored in the sensitivity analysis including observational studies. The sensitivity analysis also suggests everolimus may be more effective than axitinib, sorafenib and BSC for overall survival. However, there is evidence of inconsistency between the direct and indirect evidence for axitinib, sorafenib and everolimus, which indicates that there is heterogeneity between the studies and highlights the uncertainty around the true estimates of the relative effect of these

For peer review only - http://bmjopen.bmj.com/site/about/guidelines.xhtml

treatments. Raw data for OS and all other outcomes are available in the supplementary file (Tables 5 to 9).

Table 2. Results of the network meta-analyses for the primary outcomes (OS and PFS) and grade 3 or 4 adverse events

	**Primary NMA of RCTs**		**Sensitivity NMA of RCTs and observational studies**
**Overall survival**	Probability most effective (%)	HR *versus* everolimus (95% credible interval)	
Lenvatinib+everolimus	61	**0.61 (0.36 to 0.96)**	**0.61 (0.36 to 0.96)**
Cabozantinib	28	**0.66 (0.53 to 0.82)**	**0.66 (0.53 to 0.83)**
Nivolumab	10	**0.74 (0.57 to 0.93)**	**0.74 (0.57 to 0.93)**
Axitinib	-	-	1.14 (0.95 to 1.37)
Sorafenib	-	-	1.38 (1.12 to 1.68)
BSC	2	1.90 (0.61 to 4.53)	1.90 (0.60 to 4.56)
**Progression-free survival**	Probability best (%)	HR *versus* everolimus (95% credible interval)	
Lenvatinib+everolimus	67	**0.47 (0.26 to 0.77)**	**0.47 (0.26 to 0.77)**
Cabozantinib	34	**0.51 (0.41 to 0.63)**	**0.51 (0.41 to 0.63)**
Axitinib	-	-	0.84 (0.70 to 1.00)
Sorafenib	-	**-**	1.17 (0.95 to 1.43)
BSC	0	3.06 (2.31 to 3.97)	3.06 (2.31 to 3.97)
**Grade 3 or 4 adverse events**	Probability least harmful (%)	OR *versus* everolimus (95% credible interval)	
Lenvatinib+everolimus	0	2.67 (1.05 to 5.68)	-
Cabozantinib	0	1.66 (1.18 to 2.27)	-
Nivolumab	100	0.40 (0.29 to 0.55)	-

Progression-free survival

As with OS, lenvatinib with everolimus and cabozantinib both showed statistically significant benefits over everolimus, and lenvatinib with everolimus had the highest probability (66.5%) of being the most effective treatment out of those compared in the primary analysis of PFS (Table 2). The results of the sensitivity analysis including observational study data indicate that axitinib also improves PFS compared with everolimus, whereas BSC leads to significantly shorter PFS compared with everolimus, and there was no statistically significant difference

For peer review only

For peer review only - http://bmjopen.bmj.com/site/about/guidelines.xhtml

between everolimus and sorafenib. For PFS there was no evidence of inconsistency between the direct and indirect evidence of axitinib, sorafenib and everolimus.

Nivolumab was not included in the analyses of PFS because the proportional hazards assumption does not hold for this outcome in CheckMate 025.[35]

Objective response rate

Two of the four RCTs that could be included in the NMA for ORR observed no events in one treatment arm (everolimus in HOPE 205[15] and BSC in RECORD-1[29, 37]), causing the results from the NMA to be unreliable and lack face validity. Results using a 0.5 correction for 0 values indicate that treatment with cabozantinib, lenvatinib with everolimus, and nivolumab all lead to a better response rates than treatment with everolimus, which in turn in significantly better than BSC (supplementary file, Table 10).

Adverse effects

In terms of safety, nivolumab had the highest probability of being least harmful, i.e. the rate of grade 3 or 4 AEs was significantly lower with nivolumab (18.7%) than with everolimus (36.5%),[35] whereas treatment with either cabozantinib or lenvatinib with everolimus resulted in significantly higher rates of grade 3 or 4 AEs than everolimus (METEOR[13]: cabozantinib 71.0%, everolimus 59.9%; HOPE 205[15]: lenvatinib + everolimus 71%, everolimus 50%). Rates of grade 3 or 4 AEs were not reported for axitinib or BSC in AXIS and RECORD-1.[36, 37]

Health-related quality of life

Treatments could not be compared using NMA for HRQoL as different measures and tools were used for assessments. HRQoL scores were similar between axitinib and sorafenib in

For peer review only - http://bmjopen.bmj.com/site/about/guidelines.xhtml

AXIS[36] and results favoured nivolumab over everolimus in CheckMate 025.[35] Results in RECORD-1[29] favoured BSC over everolimus, although this effect was only apparent if models were used to account for data not missing at random. METEOR[13] results were similar for everolimus and cabozantinib. HRQoL was not measured in HOPE 205[15]. A summary of results from each of the five RCTs is provided in the Supplementary File.

DISCUSSION

This systematic review and network meta-analysis suggests that lenvatinib with everolimus, cabozantinib and nivolumab all prolong PFS and are likely to increase OS compared to everolimus for people with amRCC previously treated with VEGF-targeted therapy. The results suggest lenvatinib with everolimus is likely to be the most effective treatment, followed by cabozantinib and then nivolumab, but there is considerable uncertainty around how they compare to each other and how much better they are than the ealier generation of targeted treatments, axitinib and sorafenib. Nivolumab may be associated with fewer grade 3 or 4 AEs than treatment with both lenvatinib with everolimus and cabozantinib. All treatments considered in this review appear to delay disease progression and prolong survival more than providing BSC, and results for ORR support the primary OS and PFS analyses. Due to differences in reporting and HRQoL tools used, it was not possible to perform NMAs on safety or HRQoL.

This is a robust and comprehensive systematic review and network meta-analysis based on the principles published by Centre for Reviews and Dissemination[21] using the MOOSE[39] and PRISMA[40] reporting guidelines, and conducted according to prespecified methods in a prospectively registered protocol (PROSPERO CRD42017071540). The inclusion of all recently

For peer review only - http://bmjopen.bmj.com/site/about/guidelines.xhtml

approved treatments increases the relevance and timeliness of the review. The review is also highly relevant as it focuses on the effectiveness and safety of these treatments when used after first line TKI treatment, as recommended in clinical guidelines.[12] However, there is not enough evidence available to answer questions about the sequencing of later lines of treatments.

Although this study focuses on high quality RCT evidence, the inclusion criteria were widened to incorporate comparative observational evidence in sensitivity analyses to enable estimates for axitinib and sorafenib, which otherwise could not be connected to the network.

However, the robustness of the evidence in this review is limited by several factors:

1) PFS for nivolumab compared with the other treatments could not be estimated in this review because the proportional hazards assumption didn't hold for this outcome in the one trial including nivolumab.[35]
2) Relevant RCT data for axitinib and sorafenib were limited to a subgroup analysis conducted in one study that did not connect to the network of other RCTs.[36] Axitinib and sorafenib could only be compared to the other treatment options by including observational studies which were generally at a serious risk of different kinds of bias.
3) The trial assessing the efficacy of lenvatinib with everolimus is a small phase II trial, with an alpha set to 0.15 and therefore a higher than usual risk of false positive results and overestimation of the treatment effect. In this trial there were also some differences in baseline characteristics likely to lead to an over estimation of the treatment effect of lenvatinib and everolimus compared with everolimus, which introduces uncertainty around the true treatment effect.

For peer review only - http://bmjopen.bmj.com/site/about/guidelines.xhtml

4) Although the baseline characteristics were well balanced within most of the trials, there were some differences in performance status and number of prior VEGF targeted treatments between the trials. There were also differences in trial design with some trials being double blind or open label. Outcome assessment was not always done by an independent review committee (IRC). However, in the nivolumab trial, CheckMate 025[35], progression was only assessed by non-blinded trial investigator. There were too few studies to explore the effects of these differences between studies, which is a limitation and increases the uncertainty of the results.

5) The number of studies identified prevented meaningful subgroup analyses to explore potentially important prognostic factors that varied across the included studies. For example, while the review was limited to populations who had received prior VEGF therapy, there was variation in eligibility and baseline criteria regarding the type of VEGF treatment received and number of prior lines (see Table 1).

Two NMAs of different subsets of treatments for previously treated amRCC have recently been published.[41, 42] Unlike these studies, this review provides an alternative approach and a comparison between all recently approved treatments. Rassy *et al.*[42] and Amzal *et al.*[41] combine evidence for people who had either received prior cytokines or VEGF-targeted agents. This enabled a connected network using only RCT data, but type of prior treatment has been shown to be a potential treatment effect modifier,[36] which could introduce bias into the analysis. In addition, results for people who have only had prior cytokines are less relevant to clinical practice than for prior VEGF-targeted treatments as most people receive a TKI first line, in line with clinical guidelines.[12] The NMAs of Amzal *et al.*[41] and Rassy *et al.*[42] are also

For peer review only

For peer review only - http://bmjopen.bmj.com/site/about/guidelines.xhtml

limited by the reliance on the TARGET trial[43] to link axitinib and sorafenib to the network analysed. TARGET[43] is an RCT of sorafenib and placebo in which people only had prior cytokines and not prior TKI. The results from the TARGET trial are also confounded by crossover, which has only been partly accounted for by using immature data censored at crossover, and the lack of proportional hazards between the trial arms for PFS and OS.

For the trials that are shared between Amzal *et al*.[41] and this review and Rassy *et al*.[42] and this review the order of treatments, in terms of OS and PFS, is similar. However, this systematic review focuses specifically on the most relevant population, who have previously received a VEGF-targeted treatment, and avoids the issues with the TARGET[43] trial by including both randomised and observational evidence, and thereby provides more relevant and reliable estimates of the relative efficacy between all the interventions.

Neither prior review planned to assessed ORR or HRQoL and so these outcomes cannot be compared with previous results. A narrative presentation of adverse events in Rassy *et al*.[42] is in line with our findings that lenvatinib with everolimus is likely to be less well tolerated than nivolumab; Rassy and colleagues highlight that similarly high proportions of patients experienced Grade 3–4 adverse events and discontinued treatment due to toxicity on cabozantinib and lenvatinib with everolimus, and the most commonly reported tolerability issues across treatments were fatigue and diarrhoea.

All treatments considered in this review delay disease progression and prolong survival more than BSC, and although this review gives an indication of the ranking of the most effective treatments for treating recurrent amRCC there is still much uncertainty around how much

For peer review only - http://bmjopen.bmj.com/site/about/guidelines.xhtml

these treatments differ from each other in terms of effectiveness and safety. The choice of treatment should take into account patient preference, comorbidities, symptoms, tumour burden and how aggressive the cancer is. Policy makers also need to consider the cost-effectiveness of the treatments.

It would be preferable to have high quality RCT data comparing all the available RCC treatment options, but this is unlikely to be commissioned due to the high costs of clinical trials. However, what is more likely and still needed is a larger RCT of lenvatanib with everolimus to confirm the efficacy data from the current phase II trial with its small sample size. RCT data of axitinib and sorafenib versus other comparators in the network are also required to enable higher quality evidence for these comparisons. As there is no cure for amRCC and as virtually all people progress, research is needed into the development of resistance to treatments. Further research is also required into the impact of different sequencing of drugs from second line and onwards as more people are well enough to tolerate additional lines of treatment and most of these drugs are approved for use also beyond second line (cabozantinib, everolimus, and nivolumab).

AUTHOR CONTRIBUTIONS

Charlotta Karner PhD validated data extraction, carried out and validated meta-analyses, and drafted and edited the manuscript. Kayleigh Kew MA (Cantab) carried out additional searches, contributed to the appraisal of title and abstracts, assessment of full publications for inclusion, data extraction and validation, carried out meta-analyses, and drafted the manuscript. Victoria Wakefield MBChB devised and carried out database searches, contributed to the appraisal of title and abstracts, assessment of full publications for

For peer review only

For peer review only - http://bmjopen.bmj.com/site/about/guidelines.xhtml

inclusion, and data extraction. Natalie Masento PhD contributed to the appraisal of title and abstracts, the assessment of full publications for inclusion, and data extraction. Steven Edwards DPhil supervised the production of the manuscript and acted as methodological advisor.

ACKNOWLEDGEMENTS

The authors would like to thank Clare Fiatikoski (BMJ) and George Osei-Assibey (BMJ) for their contribution to the original literature searching and data extraction underpinning this review, and Samantha Barton (BMJ) for providing comments on the draft manuscript. The authors would also like to thank Dr Amit Bahl (University Hospitals Bristol NHS Foundation Trust, Bristol), Dr Lisa Pickering (St Georges Hospital, London), Dr Sarah Rudman (Guys and St Thomas Hospital, London), Dr Penny Kechagioglou (University Hospitals Coventry and Warwickshire, Coventry), Professor Martin Gore (Royal Marsden NHS Foundation Trust, London) and Dr Naveed Sarwar (Imperial College Healthcare NHS Trust, London) for providing clinical advice throughout the original project.

COMPETING INTERESTS

No competing interests were declared which affect the impartiality of this report. BMJ-TAG and the editorial team of *The BMJ* work independently to one another.

FUNDING

The review presents an update and extension to independent research funded by the UK National Institute for Health Research (NIHR) health technology assessment programme (HTA 16/58/01). The views and opinions expressed in this report are those of the authors and not necessarily those of the NHS, the NIHR or the Department of Health and Social Care.

For peer review only

FIGURE LEGENDS

Figure 1. PRISMA diagram

Abbreviation: RCT, randomised controlled trials

Figure 2. Network diagram

For peer review only

For peer review only - http://bmjopen.bmj.com/site/about/guidelines.xhtml

REFERENCE LIST

1. Ferlay J, Steliarova-Foucher E, Lortet-Tieulent J, Rosso S, Coebergh JW, Comber H, et al. Cancer incidence and mortality patterns in Europe: estimates for 40 countries in 2012. *Eur J Cancer* 2013; **49**: 1374-403.
2. Cancer Research UK (CRUK). Kidney Cancer Statistics. *Available at: http://wwwcancerresearchukorg/health-professional/cancer-statistics/statistics-by-cancer-type/kidney-cancer#heading-Zero Accessed: 10 March 2016* 2015.
3. European Medicines Agency (EMA). Sutent (sunitinib) European public assessment report (EPAR), 2007. Available from: https://www.ema.europa.eu/documents/overview/sutent-epar-summary-public_en.pdf. Date accessed: 14 Nov 2018.
4. European Medicines Agency (EMA). Nexavar (sorafenib) European public assessment report (EPAR), 2009. Available from: https://www.ema.europa.eu/documents/overview/nexavar-epar-summary-public_en.pdf. Date accessed: 14 Nov 2018.
5. European Medicines Agency (EMA). Afinitor (everolimus) European public assessment report (EPAR), 2010. Available from: https://www.ema.europa.eu/documents/overview/afinitor-epar-summary-public_en.pdf. Date accessed: 14 Nov 2018.
6. European Medicines Agency (EMA). Inlyta (axitinib) European public assessment report (EPAR), 2012. Available from: https://www.ema.europa.eu/documents/overview/inlyta-epar-summary-public_en.pdf. Date accessed: 14 Nov 2018.
7. European Medicines Agency (EMA). Votrient (pazopanib) European public assessment report (EPAR), 2013. Available from: https://www.ema.europa.eu/documents/overview/votrient-epar-summary-public_en.pdf. Date accessed: 14 Nov 2018.
8. European Medicines Agency (EMA). Cabometyx (cabozantinib) European public assessment report (EPAR), 2016. Available from: https://www.ema.europa.eu/documents/overview/cabometyx-epar-medicine-overview_en.pdf. Date accessed: 13 Nov 2018.
9. European Medicines Agency (EMA). Kisplyx (lenvatinib) European public assessment report (EPAR), 2016. Available from: https://www.ema.europa.eu/documents/overview/kisplyx-epar-summary-public_en.pdf. Date accessed: 14 Nov 2018.
10. European Medicines Agency (EMA). Opdivo (nivolumab) European public assessment report (EPAR), 2015. Available from: https://www.ema.europa.eu/documents/overview/opdivo-epar-summary-public_en.pdf. Date accessed: 14 Nov 2018.
11. Rodriguez-Vida A, Strijbos M, Hutson T. Predictive and prognostic biomarkers of targeted agents and modern immunotherapy in renal cell carcinoma. *ESMO Open* 2016; **1**: e000013.
12. Escudier B, Porta C, Schmidinger M, Rioux-Leclercq N, Bex A, Khoo V, et al. Renal cell carcinoma: ESMO Clinical Practice Guidelines for diagnosis, treatment and follow-up. *Ann Oncol* 2016; **27**: v58 – v68.

For peer review only - http://bmjopen.bmj.com/site/about/guidelines.xhtml

13. Choueiri T, Escudier B, Powles T, Tannir N, Mainwaring P, Rin iB. Cabozantinib versus everolimus in advanced renal cell carcinoma (METEOR): final results from a randomised, open-label, phase 3 trial. *The Lancet Oncology* 2016; **17**: 917-27.
14. Motzer RJ, Escudier B, McDermott DF, George S, Hammers HJ, Srinivas S, et al. Nivolumab versus Everolimus in Advanced Renal-Cell Carcinoma. *New England Journal of Medicine* 2015; **373**: 1803-13.
15. Motzer RJ, Hutson TE, Glen H, Michaelson MD, Molina A, Eisen T, et al. Lenvatinib, everolimus, and the combination in patients with metastatic renal cell carcinoma: a randomised, phase 2,open-label, multicentre trial. *Lancet Oncol* 2015; **16**: 1473-82.
16. Larkin J, Paine A, Tumur I, Cappelleri JC, Healey PJ, Sr., Foley G, et al. Second-line treatments for the management of advanced renal cell carcinoma: systematic review and meta-analysis. *Expert Opin Pharmacother* 2013; **14**: 27-39.
17. Dranitsaris G, Schmitz S, Broom RJ. Small molecule targeted therapies for the second-line treatment for metastatic renal cell carcinoma: a systematic review and indirect comparison of safety and efficacy. *J Cancer Res Clin Oncol* 2013; **139**: 1917-26.
18. Wang H, Man L, Li G, Huang G, Wang J. Comparative efficacy and safety of axitinib versus sorafenib in metastatic renal cell carcinoma: a systematic review and meta-analysis. *Onco Targets Ther* 2016; **9**: 3423-32.
19. Heng DY, Signorovitch J, Swallow E, Li N, Zhong Y, Qin P, et al. Comparative Effectiveness of Second-Line Targeted Therapies for Metastatic Renal Cell Carcinoma: A Systematic Review and Meta-Analysis of Real-World Observational Studies. *PLoS ONE [Electronic Resource]* 2014; **9**: e114264.
20. Motzer RJ, Escudier B, Tomczak P, Hutson TE, Michaelson MD, Negrier S, et al. Axitinib versus sorafenib as second-line treatment for advanced renal cell carcinoma: overall survival analysis and updated results from a randomised phase 3 trial, 2013. Available from: http://onlinelibrary.wiley.com/o/cochrane/clcentral/articles/055/CN-00864055/frame.html. Date accessed.
21. Centre for Reviews and Dissemination (CRD). CRD's guidance for undertaking reviews in healthcare, 2011. Date accessed: 24 July 2017.
22. Higgins JP, Altman DG, Gotzsche PC, Juni P, Moher D, Oxman AD, et al. The Cochrane Collaboration's tool for assessing risk of bias in randomised trials. *Bmj* 2011; **343**: d5928.
23. Sterne JAC, Hernán MA, Reeves BC, Savović J, Berkman ND, Viswanathan M, et al. ROBINS-I: a tool for assessing risk of bias in non-randomized studies of interventions. *BMJ* 2016; **355**: i4919.
24. Dias S, Welton NJ, Sutton AJ, Ades AE. NICE DSU Technical Support Document 1: introduction to evidence synthesis for decision making. *Available at http://wwwnicedsuorguk/TSD1%20Introductionfinal080512pdf Accessed 7 March 2017* 2011 (updated 2012).
25. Lunn DJ, Thomas A, Best N, Spiegelhalter D. WinBUGS – A Bayesian modelling framework: Concepts, structure, and extensibility. *Statistics and Computing* 2000; **10**: 325-37.
26. Woods BS, Hawkins N, Scott DA. Network meta-analysis on the log-hazard scale, combining count and hazard ratio statistics accounting for multi-arm trials: a tutorial. *BMC Med Res Methodol* 2010; **10**: 54.

For peer review only - http://bmjopen.bmj.com/site/about/guidelines.xhtml

27. Iacovelli R, Santini D, Rizzo M, Felici A, Santoni M, Verzoni E, et al. Bone metastases affect prognosis but not effectiveness of third-line targeted therapies in patients with metastatic renal cell carcinoma. *Canadian Urological Association Journal* 2015; **9**: 263-7.
28. Motzer RJ, Escudier B, McDermott DF, George S, Hammers HJ, Srinivas S, et al. Nivolumab versus Everolimus in Advanced Renal-Cell Carcinoma, 2015. Available from: http://onlinelibrary.wiley.com/o/cochrane/clcentral/articles/537/CN-01108537/frame.html. Date accessed.
29. Motzer RJ, Escudier B, Oudard S, Hutson TE, Porta C, Bracarda S, et al. Efficacy of everolimus in advanced renal cell carcinoma: a double-blind, randomised, placebo-controlled phase III trial, 2008. Available from: http://onlinelibrary.wiley.com/o/cochrane/clcentral/articles/982/CN-00649982/frame.html. Date accessed.
30. Vogelzang NJ, Pal SK, Signorovitch JE, Reichmann WM, Li N, Yang C, et al. Comparative effectiveness of everolimus and axitinib as second targeted therapies for metastatic renal cell carcinoma in the US: A retrospective chart review. *Current Medical Research and Opinion* 2016; **32**: 741-7.
31. Wong MK, Yang H, Signorovitch JE, Wang X, Liu Z, Liu NS, et al. Comparative outcomes of everolimus, temsirolimus and sorafenib as second targeted therapies for metastatic renal cell carcinoma: A US medical record review. *Curr Med Res Opin* 2014; **30**: 537-45.
32. Arranz Arija J, Perez-Valderrama B, Rodriguez Sanchez A, Puertas Alvarez J, Pinto Marin A, Maximiano Alonso C, et al. SPAZO2 (SOGUG): Comparative effectiveness of everolimus (Ev) vs axitinib (Ax) as second-line after first-line pazopanib (1stPz) in metastatic renal carcinoma (mRC). *Ann Oncol* 2017; **28 (Supplement 5)**: v313.
33. Guida A, Albiges L, Derosa L, Loriot Y, Massard C, Fizazi K, et al. Everolimus Versus Axitinib as Second-line Therapy in Metastatic Renal Cell Carcinoma: Experience From Institut Gustave Roussy. *Clin Genitourin Cancer* 2017; **15**: e1081-e8.
34. Lakomy R, Poprach A, Bortlicek Z, Melichar B, Chloupkova R, Vyzula R, et al. Utilization and efficacy of second-line targeted therapy in metastatic renal cell carcinoma: Data from a national registry. *BMC Cancer* 2017; **17 (1) (no pagination)**.
35. Motzer RJ, Escudier B, McDermott DF, George S, Hammers HJ, Srinivas S, et al. Nivolumab versus Everolimus in Advanced Renal-Cell Carcinoma. *N Engl J Med* 2015; **373**: 1803-13.
36. Motzer RJ, Escudier B, Tomczak P, Hutson TE, Michaelson MD, Negrier S, et al. Axitinib versus sorafenib as second-line treatment for advanced renal cell carcinoma: overall survival analysis and updated results from a randomised phase 3 trial. *Lancet Oncol* 2013; **14**: 552-62.
37. Motzer RJ, Escudier B, Oudard S, Hutson TE, Porta C, Bracarda S, et al. Phase 3 trial of everolimus for metastatic renal cell carcinoma : final results and analysis of prognostic factors, 2010. Available from: http://onlinelibrary.wiley.com/o/cochrane/clcentral/articles/159/CN-00762159/frame.html. Date accessed.
38. Motzer R, Sharma P, McDermott D, George S, HJ H, Srinivas S, editors. CheckMate 025 phase III trial: Outcomes by key baseline factors and prior therapy for nivolumab (NIVO) versus everolimus (EVE) in advanced renal cell carcinoma (RCC). ESMO 2016; 2016; Copenhagen: Journal of Clinical Oncology.

For peer review only - http://bmjopen.bmj.com/site/about/guidelines.xhtml

39. Stroup DF, Berlin JA, Morton SC, Olkin I, Williamson GD, Rennie D, et al. Meta-analysis of observational studies in epidemiology: a proposal for reporting. Meta-analysis Of Observational Studies in Epidemiology (MOOSE) group. *JAMA* 2000; **283**: 2008-12.
40. Moher D, Liberati A, Tetzlaff J, Altman DG, Group P. Preferred reporting items for systematic reviews and meta-analyses: the PRISMA statement. *BMJ* 2009; **339**: b2535.
41. Amzal B, Fu S, Meng J, Lister J, Karcher H. Cabozantinib versus everolimus, nivolumab, axitinib, sorafenib and best supportive care: A network meta-analysis of progression-free survival and overall survival in second line treatment of advanced renal cell carcinoma. *PLoS One* 2017; **12**: e0184423.
42. El Rassy E, Aoun F, Sleilaty G, Kattan J, Banyurwabuke B, Zanaty M, et al. Network meta-analysis of second-line treatment in metastatic renal cell carcinoma: efficacy and safety. *Future Oncol* 2017; **13**: 2709-17.
43. Escudier B, Eisen T, Stadler WM, Szczylik C, Oudard S, Siebels M, et al. Sorafenib in advanced clear-cell renal-cell carcinoma. *N Engl J Med* 2007; **356**: 125-34.

For peer review only

For peer review only - http://bmjopen.bmj.com/site/about/guidelines.xhtml

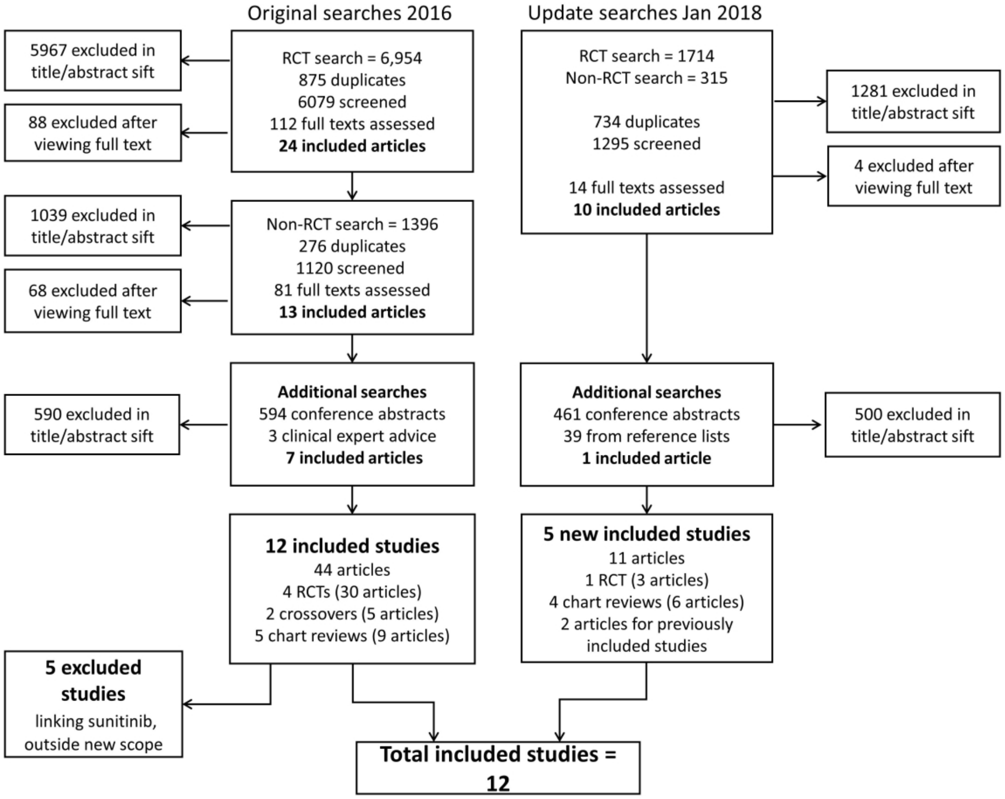

Figure 1. PRISMA diagram

288x243mm (300 x 300 DPI)

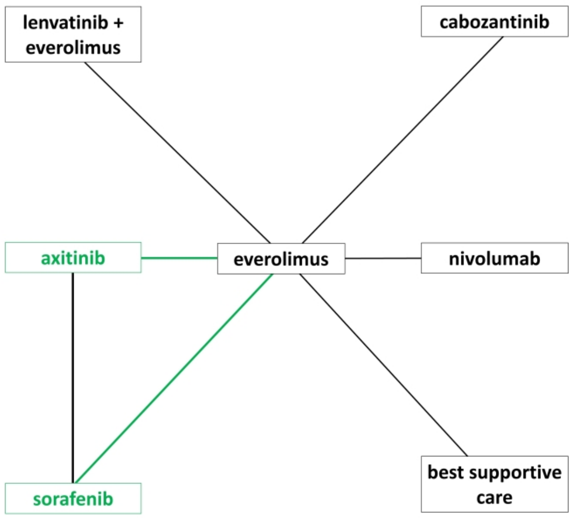

Figure 2. Network diagram

513x289mm (300 x 300 DPI)

SUPPLEMENTARY FILE

Targeted therapies for previously treated advanced or metastatic renal cell carcinoma: systematic review and network meta-analysis

Contents

SEARCH STRATEGY 2
DATA EXTRACTION 4
RISK OF BIAS SUMMARIES 7
DATASETS 9
WINBUGS CODE 12
RESULTS 14
REFERENCES 15

For peer review only

For peer review only - http://bmjopen.bmj.com/site/about/guidelines.xhtml

SUPPLEMENTARY FILE

Targeted therapies for previously treated advanced or metastatic renal cell carcinoma: systematic review and network meta-analysis

SEARCH STRATEGY

Table 1. Example search strategy (EMBASE update search for randomised controlled trials)

OVID: EMBASE 1974 to July 03 (searched on 4th July 2017 from Week 3 2016 to Week 27 2017)		
**#**	**Search Terms**	**Results**
1	Carcinoma, Renal Cell/	20712
2	(renal cell carcinoma$ or cell renal carcinoma$ or renal carcinoma$ or kidney carcinoma$ or kidney cell carcinoma$ or renal adenocarcinoma$ or kidney adenocarcinoma$ or adenocarcinoma$renal or adenocarcinoma$kidney$).mp.	66950
3	(hypernephroma$ or nephroid carcinoma$ or hypernephroid carcinoma$ or kidney hypernephroma$ or kidney pelvic carcinoma$ or kidney pyelocarcinoma$ or renal hypernephroma$ or grawitz tumo?r$ or renal cell neoplasm$ or renal cell cancer$ or renal tumo?r$ or carcinoma chromophobe cell kidney$ or chromophobe cell kidney carcinoma$).mp.	17922
4	kidney neoplasms/	10255
5	(cancer$ adj2 kidney$1).ti,ab.	5836
6	(neoplasm$1 adj2 kidney$1).ti,ab.	329
7	(neoplasm$1 adj2 renal).ti,ab.	2153
8	(cancer$ adj2 renal).ti,ab.	12586
9	(tumo?r$1 adj2 kidney$1).ti,ab.	4838
10	(tumo?r$1 adj2 renal).ti,ab.	14674
11	or/1-10	92199
12	(axitinib or inlyta or AG013736 or "AG 013736").mp.	3492
13	(sorafenib or nexavar or bay 43-9006 or bay 439006 or bay43-9006 or bay439006).mp.	23166
14	(sunitinib or sutent or pha 2909040ad or pha2909040ad or "su 010398" or "su 011248" or su 10398 or su10398 or su 11248 or su010398 or su011248 or su11248).mp.	18935
15	(everolimus or afinitor or certican or zortress or nvp-rad-001 or rad-001 or rad 001a or rad001 or rad001a or sdz rad).mp.	23010
16	(nivolumab or opdivo or ONO4538 or ONO 4538 or BMS936558 or BMS 936558 or MDX1106 or MDX 1106).mp.	4666
17	(temsirolimus or cci-779 or cell-cycle-inhibitor-779 or nsc 683864 or nsc683864 or torisel).mp.	7267
18	(bevacizumab or avastin or nsc 704865 or nsc704865 or anti-vegf or rhumab-vegf).mp.	50865
19	(armala or pazopanib or gw786034 or gw 786034 or sb 710468 or sb710468 or votrient).mp.	5612
20	or/12-19	99220
21	Clinical trial/	934498
22	Randomized controlled trial/	460454
23	Randomization/	74486
24	Single blind procedure/	28210
25	Double blind procedure/	140589
26	Crossover procedure/	52369
27	Placebo/	309726
28	Randomi?ed controlled trial$.tw.	162409
29	Rct.tw.	24817
30	Random allocation.tw.	1704
31	Randomly allocated.tw.	28013
32	Allocated randomly.tw.	2271
33	(allocated adj2 random).tw.	867
34	Single blind$.tw.	19692
35	Double blind$.tw.	180274
36	((treble or triple) adj blind$).tw.	721
37	Placebo$.tw.	257991
38	Prospective study/	388181

For peer review only - http://bmjopen.bmj.com/site/about/guidelines.xhtml

SUPPLEMENTARY FILE

Targeted therapies for previously treated advanced or metastatic renal cell carcinoma: systematic review and network meta-analysis

39	or/21-38	1782373
40	Case study/	48285
41	Case report.tw.	342862
42	Abstract report/ or letter/	1025233
43	or/40-42	1408381
44	39 not 43	1736655
45	11 and 20 and 44	3942
46	Animals/ not Humans/	1295518
47	45 not 46	3942
48	(editorial or letter).pt.	1521255
49	47 not 48	3868
50	limit 49 to em=201603-201727	327

For peer review only

For peer review only - http://bmjopen.bmj.com/site/about/guidelines.xhtml

SUPPLEMENTARY FILE

Targeted therapies for previously treated advanced or metastatic renal cell carcinoma: systematic review and network meta-analysis

DATA EXTRACTION

Table 2. Data extraction template

**Study or trial name:**			**Publication source**
**Full reference for all publications:**			
**Design**			
Study design			
Number of centres & Country/countries			
Recruitment dates			
Length of follow-up [include study start date, data cut-off and completion date]			
Source of funding			
Eligibility criteria (inclusion and exclusion)			
**Participants and treatment arms**	**Intervention:**	**Comparator:**	**Publication, data cut-off**
Intervention, method of delivery, dose and frequency			
Concomitant medication(s) or therapies			
Cross-over or post-study interventions allowed<br>Number of patients (%)			
Number of cycles<br>At least one dose reduction n (%)			
Treatment duration (and the data cut offs for each publication for the study)			
Number randomised			
Number who received study medication			
Number withdrawn/ discontinued and reasons<br>[give breakdown]			
Disease stage and/or metastatic disease<br>[give breakdown]			
Previous systemic therapy treatments, n (%) [give breakdown]			
Age, years: median (range)			
Ethnicity, n (%)<br>[give breakdown]			
Male, n (%)			
Performance status n (%)			

For peer review only

SUPPLEMENTARY FILE

Targeted therapies for previously treated advanced or metastatic renal cell carcinoma: systematic review and network meta-analysis

[give tool and breakdown]			
Reported subgroups	None reported		
**Reported outcomes**			
Primary outcome			
Secondary outcomes			
Outcomes and time points with data reported for subgroups of prior baseline therapies			
Outcomes and time points with data reported for subgroups of baseline prognostic scores (e.g. ECOG, MSKCC)			
**Results**	**Intervention**	**Comparator**	**Publication, data cut-off**
**PFS**			
HR (95% CI)			
HR (95% CI) for subgroups based on prior therapy:			
PFS, median (95% CI) months			
PFS, median (95% CI), months for subgroups based on prior therapy			
Number of progression events n (%)			
**Overall survival**			
HR, (95% CI)			
HR, (95% CI) for subgroups based on prior therapy			
Number of deaths, n (%)			
Median OS, months (95% CI)			
Median OS, (95% CI) months for subgroup based on prior therapy			
Number of deaths, n (%) for subgroups based on prior therapy			
**Response**			
Objective response, n (%)			
Complete response, n (%)			
Partial response, n (%)			
Stable disease, n (%)			
Progressive disease, n (%)			
Time to response, months (median [range])			
Duration of response, median (95% CI), months			
Other measures of response			
**HRQoL**			

For peer review only

For peer review only - http://bmjopen.bmj.com/site/about/guidelines.xhtml

SUPPLEMENTARY FILE

Targeted therapies for previously treated advanced or metastatic renal cell carcinoma: systematic review and network meta-analysis

[Scale 1] Mean end of treatment			
[Scale 1] Mean difference (95% CI)			
Completion rate			
**Adverse events (AE's)**			
N in safety analysis			
Total AE's (any Grade)			
Total AE's Grade ≥3			
[enter list of individual AEs]			

**Risk of bias assessment based (RCTs)**		
**Domain**	**Risk assessment**	**Comments**
Random sequence generation	[Low/High/Unclear]	
Allocation concealment	[Low/High/Unclear]	
Blinding (who [participants, personnel], and method)	[Low/High/Unclear]	
Other biases	[Low/High/Unclear]	
Progression-free survival		
-Blinding of outcome assessment	[Low/High/Unclear]	
-Incomplete outcome data	[Low/High/Unclear]	
-Selective reporting	[Low/High/Unclear]	
Overall survival		
-Blinding of outcome assessment	[Low/High/Unclear]	
-Incomplete outcome data	[Low/High/Unclear]	
-Selective reporting	[Low/High/Unclear]	
Response (partial response, disease stabilisation, progressive disease)		
-Blinding of outcome assessment	[Low/High/Unclear]	
-Incomplete outcome data	[Low/High/Unclear]	
-Selective reporting	[Low/High/Unclear]	
HRQoL		
-Blinding of outcome assessment	[Low/High/Unclear]	
-Incomplete outcome data	[Low/High/Unclear]	
-Selective reporting	[Low/High/Unclear]	
Adverse events		
-Blinding of outcome assessment	[Low/High/Unclear]	
-Incomplete outcome data	[Low/High/Unclear]	
-Selective reporting	[Low/High/Unclear]	

For peer review only - http://bmjopen.bmj.com/site/about/guidelines.xhtml

SUPPLEMENTARY FILE

Targeted therapies for previously treated advanced or metastatic renal cell carcinoma: systematic review and network meta-analysis

RISK OF BIAS SUMMARIES

Table 3. Summary of Cochrane risk of bias assessment for randomised control trials

**Criteria**		**AXIS**	**Checkmate -025**	**HOPE 205**	**METEOR**	**RECORD-1**
All outcomes	Random sequence generation	✓	✓	✓	✓	✓
	Allocation concealment	✓	✓	✓	✓	✓
	Blinding: participant/personnel	x	X	X	X	✓
**Outcome-specific**						
OS	Blinding: outcome assessment	✓	✓	✓	✓	✓
	Incomplete outcome data	✓	?	✓	?	✓
	Selective Reporting	✓	✓	✓	✓	✓
	Other Biases	?	?	?	?	?
PFS	Blinding: outcome assessment	✓	X	✓	✓	✓
	Incomplete outcome data	✓	✓	✓	?	✓
	Selective Reporting	✓	✓	✓	✓	✓
	Other Biases	**NA**	**NA**	?	**NA**	**NA**
ORR	Blinding: outcome assessment	✓	X	✓	✓	✓
	Incomplete outcome data	?	✓	✓	?	✓
	Selective Reporting	✓	✓	✓	✓	?
	Other Biases	**NA**	**NA**	?	**NA**	**NA**
HRQoL	Blinding: outcome assessment	x	X	**NA**	X	x
	Incomplete outcome data	✓	✓	**NA**	?	✓
	Selective Reporting	✓	✓	**NA**	✓	x
	Other Biases	**NA**	**NA**	**NA**	**NA**	**NA**
AE	Blinding: outcome assessment	x	X	X	X	✓
	Incomplete outcome data	✓	✓	✓	✓	✓
	Selective Reporting	✓	✓	✓	✓	✓
	Other Biases	**NA**	**NA**	?	**NA**	**NA**

Key: ✓, low risk; ?, unclear risk; X, high risk; NA, not applicable.
Abbreviations: OS, overall survival; PFS, progression-free survival; ORR, objective response rate; HRQoL, health-related quality of life; AE, adverse effects.

For peer review only - http://bmjopen.bmj.com/site/about/guidelines.xhtml

SUPPLEMENTARY FILE

Targeted therapies for previously treated advanced or metastatic renal cell carcinoma: systematic review and network meta-analysis

Table 4. Summary of ROBINS-I risk of bias assessments in non-randomised studies

	Guida 2017	Heng 2016	Iacovelli 2015	Lakomy 2017	SPAZO-2	Vogelzang 2016	Wong 2014
**Overall survival**							
Confounding	~	~	x	x	~	~	~
Selection	✓	✓	✓	✓	✓	✓	✓
Intervention classification	✓	✓	✓	✓	✓	✓	✓
Intervention deviations	✓	✓	✓	✓	✓	✓	✓
Missing data	✓	NI	✓	✓	✓	✓	x
Outcome measures	✓	✓	✓	✓	✓	✓	✓
Outcome reporting	✓	✓	✓	✓	✓	✓	✓
**Overall judgement**	x	~	x	x	~	~	x
**Progression-free survival**							
Confounding	x	~	-	x	~	~	~
Selection	✓	✓	-	✓	✓	✓	✓
Intervention classification	✓	✓	-	✓	✓	✓	✓
Intervention deviations	✓	✓	-	✓	✓	✓	✓
Missing data	✓	NI	-	✓	✓	✓	x
Outcome measures	x	x	-	x	x	x	x
Outcome reporting	x	x	-	x	x	x	x
**Overall judgement**	x	x	-	x	x	x	x

Abbreviations: PFS = progression-free survival; OS = overall survival

Key: ✓, low risk; ~, moderate risk; x, serious risk; x, critical risk; NI, no information.

For peer review only - http://bmjopen.bmj.com/site/about/guidelines.xhtml

SUPPLEMENTARY FILE

Targeted therapies for previously treated advanced or metastatic renal cell carcinoma: systematic review and network meta-analysis

DATASETS

Table 5. Study data: overall survival

Study	Data details	T1	T2	HR (95% CI)	T1 median months (95% CI)	T2 median (95% CI)
**AXIS[1]**	**Prior sunitinib subset**	**5**	**6**	**1.00 (0.78 to 1.27)**	**NR**	**NR**
**CheckMate 025[2]**	**-**	**3**	**1**	**0.73 (0.57 to 0.93)**	**25.0 (21.8 to NE)**	**19.6 (17.6 to 23.1)**
**HOPE 205[3]**	**July 2015 cutoff**	**7**	**1**	**0.59 (0.36 to 0.97)**	**25.5 (16.4 to 32.1)**	**15.4 (11.8 to 20.6)**
**METEOR[4]**	**31 Dec 2015 cutoff**	**2**	**1**	**0.66 (0.53 to 0.83)**	**21.4 (18.7 to NE)**	**16.5 (14.7 to 18.8)**
**RECORD-1[5]**	**RPSFT adjusted**	**1**	**4**	**0.60 (0.22 to 1.65)**	**14.4 (NR)**	**10.0 (NR)**
Guida 2017[6]*		5	1	1.33 (0.8 to 2.1)	14.9 (7.4 to 22.4)	21.5 (16.5 to 26.5)
Heng 2016[7]	-	6	1	1.25 (0.75 to 2.10)	18.7 (NR)	23.0 (NR)
Heng 2016[7]	-	5	1	1.22 (0.77 to 1.94)	23.5 (NR)	23.0 (NR)
Iacovelli 2015[8]	-	6	1	2.21 (1.47 to 3.31)	NR	NR
SPAZO-2[9]	Adjusted results	5	1	0.81 (0.60 to 1.20)	11.6 (7 to 16)	9.5 (7 to 12)
Vogelzang 2016[10]	-	1	5	1.16 (0.74 to 1.82)	NR	NR
Wong 2014[11]	Full adjusted results	1	6	0.66 (0.44 to 0.99)	19.0 (NR)	13.8 (NR)

Abbreviations: T1, treatment 1; T2, treatment 2 (baseline); HR, hazard ratio; CI, confidence interval; NE, not estimable; RPSFT, rank preserving structural failure time model; NR, not reported.
Treatment codes: 1, everolimus; 2, cabozantinib; 3, nivolumab; 4, best supportive care/placebo; 5, axitinib; 6, sorafenib; 7, lenvatinib with everolimus. Studies in bold formed the primary analysis.
*Data from personal communication with the study author, 11 March 2018

Table 6. Study data: progression-free survival

Study	Data details	T1	T2	HR (95% CI)	T1 median months (95% CI)	T2 median (95% CI)
**HOPE 205[12]**	**IRR**	**7**	**1**	**0.45 (0.27 to 0.79)**	**12.8 (7.4 to 17.5)**	**5.6 (3.6 to 9.3)**
**METEOR[4]**	**IRR for ITT**	**2**	**1**	**0.51 (0.41 to 0.62)**	**7.4 (6.6 to 9.1)**	**3.9 (3.7 to 5.1)**
**RECORD-1[13]**	**Final analysis, ICR**	**1**	**4**	**0.33 (0.25 to 0.43)**	**4.9 (4.0 to 5.5)**	**1.9 (1.8 to 1.9)**
AXIS[1]	Prior sunitinib subset	5	6	0.72 (0.57 to 0.90)	6.5 (5.7 to 7.9)	4.4 (2.9 to 4.7)
Guida 2017[6]*		5	1	0.84 (0.55 to 1.2)	7.7 (5.3 to 10.2)	5.3 (4.0 to 6.6)
Heng 2016[7]	-	6	1	1.47 (0.95 to 2.28)	-	-
Heng 2016[7]	-	5	1	1.26 (0.81 to 1.95)	-	-
Iacovelli 2015[8]	-	6	1	NR	NR	NR
SPAZO-2[9]	Adjusted results	5	1	0.76 (0.5 to 1.1)	5.3 (3 to 7)	4.6 (3 to 6)

For peer review only - http://bmjopen.bmj.com/site/about/guidelines.xhtml

SUPPLEMENTARY FILE

Targeted therapies for previously treated advanced or metastatic renal cell carcinoma: systematic review and network meta-analysis

Vogelzang 2016[10]	Adjusted results	1	5	1.16 (0.85 to 1.59)	NR	NR
Wong 2014[11]	Adjusted results	1	6	0.76 (0.55 to 1.04)	10.1 (NR)	8.6 (NR)

Abbreviations: T1, treatment 1; T2, treatment 2 (baseline); HR, hazard ratio; CI, confidence interval; NR, not reported.
Treatment codes: 1, everolimus; 2, cabozantinib; 3, nivolumab; 4, best supportive care/placebo; 5, axitinib; 6, sorafenib; 7, lenvatinib with everolimus. Studies in bold formed the primary analysis.
*Data from personal communication with the study author, 11 March 2018

Table 7. Study data: objective response rate

			**N**		**Objective response**	
**Study**	**T1**	**T2**	**T1**	**T2**	**T1**	**T2**
AXIS[14]	5	6	361	362	70	34
CheckMate 025[2]	3	1	410	411	103	22
HOPE 205[12]	7	1	51	50	18	0
METEOR[4]	2	1	330	328	57	11
RECORD-1[13]	1	4	277	139	5	0

Abbreviations: T1, treatment 1; T2, treatment 2 (baseline).
Treatment codes: 1, everolimus; 2, cabozantinib; 3, nivolumab; 4, best supportive care/placebo; 5, axitinib; 6, sorafenib; 7, lenvatinib with everolimus

Table 8. Study data: grade 3 or 4 adverse events

			**N**		**Grade 3 or 4 adverse events**	
**Study**	**T1**	**T2**	**T1**	**T2**	**T1**	**T2**
AXIS[1]	5	6	359	355	NR	NR
CheckMate 025[2]	3	1	406	397	76	145
HOPE 205[15]	7	1	51	50	36	25
METEOR[4]	2	1	331	322	235	193
RECORD-1[13]	1	4	274	137	NR	NR

Abbreviations: T1, treatment 1; T2, treatment 2 (baseline); HR, hazard ration; CI, confidence interval; NE, not estimable; RPSFT, rank preserving structural failure time model; NR, not reported.
Treatment codes: 1, everolimus; 2, cabozantinib; 3, nivolumab; 4, best supportive care/placebo; 5, axitinib; 6, sorafenib; 7, lenvatinib with everolimus

Table 9. Study data: health-related quality of life (not meta-analysed)

**Study**	**T1**	**T2**	**Study analysis details**	**FKSI scales**	**EuroQol scales**	**EORTC QLQ-C30**
AXIS[16]	5	6	End of treatment MD (95% CI)	DRS: MD 0.12 (-0.45 to 0.69) p = 0.68; FKSI-15: MD 0.35 (-0.63 to 1.34) p = 0.48	5D Index: MD 0.02 (-0.01 to 0.05) p = 0.19 VAS: -0.53 (-2.77 to 1.72) p = 0.65	NR

For peer review only - http://bmjopen.bmj.com/site/about/guidelines.xhtml

SUPPLEMENTARY FILE

Targeted therapies for previously treated advanced or metastatic renal cell carcinoma: systematic review and network meta-analysis

CheckMate 025[2]	3	1	Median change (range) at week 104	DRS:<br>niv -2 (-1 to 16)<br>evo 2 (-7 to 15)	NR	NR
HOPE 205	7	1	NA	NR	NR	NR
METEOR	2	1	NA	NR	NR	NR
RECORD-1[17]	1	4	Time to deterioration HR (95% CI); results favour placebo	DRS: HR 0.82, 95% CI 0.75 to 0.92, p = 0.001	NR	Global health status HR 0.85 (0.75 to 0.96) p = 0.006<br>Physical functioning HR 0.84 (0.75 to 0.94) p = 0.001
Abbreviations: FKSI-DRS = Functional Assessment of Cancer Therapy (FACT) Kidney Cancer Symptom Index; DRS = Disease-related Symptoms subscale of the FKSI-15; EQ-5D = European Quality of Life self-report questionnaire; VAS = visual analogue scale; EORTC QLQ = European Organization for the Research and Treatment of Cancer Quality of Life Questionnaire; MD, mean difference; CI, confidence interval; HR, hazard ratio; NA, not applicable; NR, not reported						

For peer review only

SUPPLEMENTARY FILE

Targeted therapies for previously treated advanced or metastatic renal cell carcinoma: systematic review and network meta-analysis

WINBUGS CODE

CODE 1: Fixed effect log hazard ratio NMA for 2-arm studies (overall survival primary and progression-free survival primary)

```
model{
#Model for log-hazard ratios
for(i in 1:ndp){
        prec[i]<- 1/(se[i]*se[i])
                lhr[i]~dnorm(md[i],prec[i])

#Fixed effect model for log hazard ratios
                md[i] <- d[t[i]] - d[b[i]]

#Deviance residuals for data i
    dev[i] <- (lhr[i] - md[i])*(lhr[i] - md[i])/(se[i]*se[i])
            }
            resdev <- sum(dev[])

#Give priors for log hazard ratios
            d[1]<-0
            for (k in 2:nt){
            d[k] ~ dnorm(0,.001)
            }

#Rank the treatment effects (with 1=best) & record the best treatment
for(k in 1:nt){
            rk[k]<- rank(d[],k)
            best[k]<-equals(rk[k],1)
            }

#All pairwise log hazard ratios and hazard ratios
for (c in 1:nt-1){
            for (k in (c+1):nt){
                        lhzr[c,k] <- d[k] - d[c]
                        HR[c,k] <- exp(lhzr[c,k])
                        }
            }
}
```

CODE 2: Fixed effect odds ratio NMA (objective response rate analysis)

```
model{

for(i in 1:ns){

            delta[i,t[i,1]]<-0
            mu[i] ~ dnorm(0,.0001)

            for (k in 1:na[i]) {
                        r[i,t[i,k]] ~ dbin(p[i,t[i,k]],n[i,t[i,k]])
```


For peer review only

For peer review only - http://bmjopen.bmj.com/site/about/guidelines.xhtml

SUPPLEMENTARY FILE

Targeted therapies for previously treated advanced or metastatic renal cell carcinoma: systematic review and network meta-analysis

```
        logit(p[i,t[i,k]])<-mu[i] + delta[i,t[i,k]]

    rhat[i,t[i,k]]<- p[i,t[i,k]] * n[i,t[i,k]]

    resdev[i,k]<- 2 * (r[i,t[i,k]] * (log(r[i,t[i,k]]) - log(rhat[i,t[i,k]])) + (n[i,t[i,k]] - r[i,t[i,k]]) * (log(n[i,t[i,k]] - r[i,t[i,k]]) -
log(n[i,t[i,k]] - rhat[i,t[i,k]]))))
    }
    sumdev[i]<-sum(resdev[i,1:na[i]])

for (k in 2:na[i]) {
    delta[i,t[i,k]] <- d[t[i,k]] - d[t[i,1]]        # trial-specific LOR
    }
}

sumdevtot<- sum(sumdev[])

d[1]<-0
for (k in 2:nt){
    d[k] ~ dnorm(0,.0001)
    }

for (i in 1:ns) {
    mu1[i] <- mu[i] * equals(t[i,1],1)
    }

for (k in 1:nt) {
    logit(T[k])<- sum(mu1[])/nb +d[k]
    }

for (k in 1:nt) {
    rk[k]<-nt - rank(T[],k)
    best[k]<-equals(rk[k],1)
    }

for (c in 1:(nt-1)) {
    for (k in (c+1):nt) {
        lor[c,k] <- (d[k] - d[c])
        or[c,k]<-exp(lor[c,k])
            }
        }
}
```

CODE 3: Fixed effect log hazard ratio NMA to combine 2-arm and multi-arm studies (overall survival sensitivity and progression-free survival sensitivity)

```
model{

# Priors

  #On tx effect mean
  beta[1] < -0
  for (tt in 2:nt){
  beta[tt]~dnorm(0,1.0E-6)
  }

  #On individual study baseline effect
```


For peer review only - http://bmjopen.bmj.com/site/about/guidelines.xhtml

SUPPLEMENTARY FILE

Targeted therapies for previously treated advanced or metastatic renal cell carcinoma: systematic review and network meta-analysis

```
    for(ss in 1:ns){
    alpha[ss] ~ dnorm(0,1.0E-6)
    }

# Fit data
    for(ii in 1:ndp){
    mu[ii] < - alpha[t[ii]]*multi[ii] + beta[tx[ii]] - beta[b[ii]]
    prec[ii] < - 1/pow(se[ii],2)
    m[ii] ~ dnorm(mu[ii],prec[ii])
    }

# Calculate HRs
    for (hh in 1:nt) {
    hr[hh] < -exp(beta[hh])
    }

# Rank
    for (ll in 1:nt) {
                    rk[ll]<-rank(beta[],ll)
     best[ll] < - equals(rk[ll],1)
      }
    }
}
```

RESULTS

Table 10. Results of the RCT network meta-analyses for objective response rate, with 0.5 correction of 0 values

	Best supportive care	Lenvatinib+ everolimus	Nivolumab	Cabozantinib	Everolimus
Everolimus	0.24 (0.00 to 1.39)	91190 (9.30 to 34400)	6.23 (3.78 to 10.01)	6.61 (3.27 to 12.55)	-
Cabozantinib	0.04 (0.00 to 0.24)	14500 (1.36 to 5629)	1.06 (0.41 to 2.19)	-	0.15 (0.08 to 0.31)
Nivolumab	0.00 (0.00 to 0.24)	7.73 (1.46 to 5652)	-	0.94 (0.46 to 2.41)	0.16 (0.10 to 0.26)
Lenvatinib + everolimus	0.01 (0.00 to 0.04)	-	0.13 (0.00 to 0.68)	0.00 (0.00 to 0.74)	0.00 (0.00 to 0.11)
BSC	-	193 (24.99 to 4494382)	657000 (4.15 to 9580)	23.53 (4.10 to 30312)	4.02 (0.72 to 4826)

Abbreviations: BSC, best supportive care; OS, overall survival; PFS, progression-free survival
Results are odds ratios with 95% credible interval; odds ratios > 1 favour the treatment along the top row.

For peer review only - http://bmjopen.bmj.com/site/about/guidelines.xhtml

SUPPLEMENTARY FILE

Targeted therapies for previously treated advanced or metastatic renal cell carcinoma: systematic review and network meta-analysis

REFERENCES

1. Motzer RJ, Escudier B, Tomczak P, Hutson TE, Michaelson MD, Negrier S, et al. Axitinib versus sorafenib as second-line treatment for advanced renal cell carcinoma: overall survival analysis and updated results from a randomised phase 3 trial, 2013. Available from: http://onlinelibrary.wiley.com/o/cochrane/clcentral/articles/055/CN-00864055/frame.html. Date accessed.
2. Motzer RJ, Escudier B, McDermott DF, George S, Hammers HJ, Srinivas S, et al. Nivolumab versus Everolimus in Advanced Renal-Cell Carcinoma, 2015. Available from: http://onlinelibrary.wiley.com/o/cochrane/clcentral/articles/537/CN-01108537/frame.html. Date accessed.
3. Hutson TE, Dutcus CE, Ren M, Baig MA, Fishman MN. Subgroup analyses and updated overall survival from the phase II trial of lenvatinib (LEN), everolimus (EVE), and LEN+EVE in metastatic renal cell carcinoma (mRCC). Journal of Clinical Oncology Conference 2016; 34.
4. Choueiri T, Escudier B, Powles T, Tannir N, Mainwaring P, Rin iB. Cabozantinib versus everolimus in advanced renal cell carcinoma (METEOR): final results from a randomised, open-label, phase 3 trial. The Lancet Oncology 2016; 17: 917-27.
5. Korhonen P, Zuber E, Branson M, al. e. Correcting overall survival for the impact of crossover via a rank-preserving structural failure time (RPSFT) model in the RECORD-1 trial of everolimus in metastatic renal-cell carcinoma. J Biopharm Stat 2012; 22: 1258-71.
6. Guida A, Albiges L, Derosa L, Loriot Y, Massard C, Fizazi K, et al. Everolimus Versus Axitinib as Second-line Therapy in Metastatic Renal Cell Carcinoma: Experience From Institut Gustave Roussy. Clinical Genitourinary Cancer 2017; 15: e1081-e8.
7. Heng DY, Signorovitch J, Swallow E, Li N, Zhong Y, Qin P, et al. Comparative Effectiveness of Second-Line Targeted Therapies for Metastatic Renal Cell Carcinoma: A Systematic Review and Meta-Analysis of Real-World Observational Studies. PLoS ONE [Electronic Resource] 2014; 9: e114264.
8. Iacovelli R, Santini D, Rizzo M, Felici A, Santoni M, Verzoni E, et al. Bone metastases affect prognosis but not effectiveness of third-line targeted therapies in patients with metastatic renal cell carcinoma. Can Urol Assoc J 2015; 9: 263-7.
9. Arranz Arija J, Perez-Valderrama B, Rodriguez Sanchez A, Puertas Alvarez J, Pinto Marin A, Maximiano Alonso C, et al. SPAZO2 (SOGUG): Comparative effectiveness of everolimus (Ev) vs axitinib (Ax) as second-line after first-line pazopanib (1stPz) in metastatic renal carcinoma (mRC). Annals of Oncology 2017; 28 (Supplement 5): v313.
10. Vogelzang NJ, Pal SK, Signorovitch JE, Reichmann WM, Li N, Yang C, et al. Comparative effectiveness of everolimus and axitinib as second targeted therapies for metastatic renal cell carcinoma in the US: A retrospective chart review. Current Medical Research and Opinion 2016; 32: 741-7.
11. Wong MK, Yang H, Signorovitch JE, Wang X, Liu Z, Liu NS, et al. Comparative outcomes of everolimus, temsirolimus and sorafenib as second targeted therapies for metastatic renal cell carcinoma: A US medical record review. Current Medical Research and Opinion 2014; 30: 537-45.
12. Motzer RJ, Hutson TE, Ren M, Dutcus C, Larkin J. Independent assessment of lenvatinib plus everolimus in patients with metastatic renal cell carcinoma, 2016. Available from: http://onlinelibrary.wiley.com/o/cochrane/clcentral/articles/453/CN-01168453/frame.html. Date accessed.
13. Motzer RJ, Escudier B, Oudard S, Hutson TE, Porta C, Bracarda S, et al. Phase 3 trial of everolimus for metastatic renal cell carcinoma : final results and analysis of prognostic factors, 2010. Available from: http://onlinelibrary.wiley.com/o/cochrane/clcentral/articles/159/CN-00762159/frame.html. Date accessed.
14. Rini BI, Escudier B, Tomczak P, Kaprin A, Szczylik C, Hutson TE, et al. Comparative effectiveness of axitinib versus sorafenib in advanced renal cell carcinoma (AXIS): a randomised phase 3 trial, 2011. Available from: http://onlinelibrary.wiley.com/o/cochrane/clcentral/articles/402/CN-00804402/frame.html. Date accessed.
15. Motzer RJ, Hutson TE, Glen H, Michaelson MD, Molina A, Eisen T, et al. Lenvatinib, everolimus, and the combination in patients with metastatic renal cell carcinoma: a randomised, phase 2,open-label, multicentre trial. Lancet Oncology 2015; 16: 1473-82.

For peer review only - http://bmjopen.bmj.com/site/about/guidelines.xhtml

SUPPLEMENTARY FILE

Targeted therapies for previously treated advanced or metastatic renal cell carcinoma: systematic review and network meta-analysis

16. Cella D, Escudier B, Rini B, Chen C, Bhattacharyya H, Tarazi J, et al. Patient-reported outcomes for axitinib vs sorafenib in metastatic renal cell carcinoma: phase III (AXIS) trial. British Journal of Cancer 2013; 108: 1571-8.
17. Beaumont JL, Butt Z, Baladi J, Motzer RJ, Haas T, Hollaender N, et al. Patient-reported outcomes in a phase iii study of everolimus versus placebo in patients with metastatic carcinoma of the kidney that has progressed on vascular endothelial growth factor receptor tyrosine kinase inhibitor therapy, 2011. Available from: http://onlinelibrary.wiley.com/o/cochrane/clcentral/articles/925/CN-00800925/frame.html

For peer review only

For peer review only - http://bmjopen.bmj.com/site/about/guidelines.xhtml

PRISMA 2009 Checklist

Section/topic	#	Checklist item	Reported on page #
**TITLE**			
Title	1	Identify the report as a systematic review, meta-analysis, or both.	1
**ABSTRACT**			
Structured summary	2	Provide a structured summary including, as applicable: background; objectives; data sources; study eligibility criteria, participants, and interventions; study appraisal and synthesis methods; results; limitations; conclusions and implications of key findings; systematic review registration number.	2-4
**INTRODUCTION**			
Rationale	3	Describe the rationale for the review in the context of what is already known.	7
Objectives	4	Provide an explicit statement of questions being addressed with reference to participants, interventions, comparisons, outcomes, and study design (PICOS).	7
**METHODS**			
Protocol and registration	5	Indicate if a review protocol exists, if and where it can be accessed (e.g., Web address), and, if available, provide registration information including registration number.	3
Eligibility criteria	6	Specify study characteristics (e.g., PICOS, length of follow-up) and report characteristics (e.g., years considered, language, publication status) used as criteria for eligibility, giving rationale.	7-9
Information sources	7	Describe all information sources (e.g., databases with dates of coverage, contact with study authors to identify additional studies) in the search and date last searched.	9-10
Search	8	Present full electronic search strategy for at least one database, including any limits used, such that it could be repeated.	Suppl. 2-3, Table 1
Study selection	9	State the process for selecting studies (i.e., screening, eligibility, included in systematic review, and, if applicable, included in the meta-analysis).	9, 11
Data collection process	10	Describe method of data extraction from reports (e.g., piloted forms, independently, in duplicate) and any processes for obtaining and confirming data from investigators.	9-10
Data items	11	List and define all variables for which data were sought (e.g., PICOS, funding sources) and any assumptions and simplifications made.	9-10, suppl. 4-6, Table 2
Risk of bias in individual studies	12	Describe methods used for assessing risk of bias of individual studies (including specification of whether this was done at the study or outcome level), and how this information is to be used in any data synthesis.	10
Summary measures	13	State the principal summary measures (e.g., risk ratio, difference in means).	11
Synthesis of results	14	Describe the methods of handling data and combining results of studies, if done, including measures of consistency (e.g., I^2) for each meta-analysis.	10-11

For peer review only

For peer review only - http://bmjopen.bmj.com/site/about/guidelines.xhtml

PRISMA 2009 Checklist

Section/topic	#	Checklist item	Reported on page #
Risk of bias across studies	15	Specify any assessment of risk of bias that may affect the cumulative evidence (e.g., publication bias, selective reporting within studies).	10
Additional analyses	16	Describe methods of additional analyses (e.g., sensitivity or subgroup analyses, meta-regression), if done, indicating which were pre-specified.	11
**RESULTS**			
Study selection	17	Give numbers of studies screened, assessed for eligibility, and included in the review, with reasons for exclusions at each stage, ideally with a flow diagram.	Fig. 1
Study characteristics	18	For each study, present characteristics for which data were extracted (e.g., study size, PICOS, follow-up period) and provide the citations.	Table 1
Risk of bias within studies	19	Present data on risk of bias of each study and, if available, any outcome level assessment (see item 12).	16-17, suppl. 7-8, Table 3-4
Results of individual studies	20	For all outcomes considered (benefits or harms), present, for each study: (a) simple summary data for each intervention group (b) effect estimates and confidence intervals, ideally with a forest plot.	Suppl. 9-11, Table 5-9
Synthesis of results	21	Present results of each meta-analysis done, including confidence intervals and measures of consistency.	17-19, Table 2, Suppl. 14, Table 10
Risk of bias across studies	22	Present results of any assessment of risk of bias across studies (see Item 15).	4, 16-18
Additional analysis	23	Give results of additional analyses, if done (e.g., sensitivity or subgroup analyses, meta-regression [see Item 16]).	17-18, Table 2
**DISCUSSION**			
Summary of evidence	24	Summarize the main findings including the strength of evidence for each main outcome; consider their relevance to key groups (e.g., healthcare providers, users, and policy makers).	20, 23
Limitations	25	Discuss limitations at study and outcome level (e.g., risk of bias), and at review-level (e.g., incomplete retrieval of identified research, reporting bias).	21-22
Conclusions	26	Provide a general interpretation of the results in the context of other evidence, and implications for future research.	22-23
**FUNDING**			
Funding	27	Describe sources of funding for the systematic review and other support (e.g., supply of data); role of funders for the systematic review.	25

For peer review only
For peer review only - http://bmjopen.bmj.com/site/about/guidelines.xhtml

PRISMA 2009 Checklist

From: Moher D, Liberati A, Tetzlaff J, Altman DG, The PRISMA Group (2009). Preferred Reporting Items for Systematic Reviews and Meta-Analyses: The PRISMA Statement. PLoS Med 6(7): e1000097. doi:10.1371/journal.pmed1000097

For more information, visit: **www.prisma-statement.org**.

For peer review only

Reporting checklist for meta-analysis of observational studies.

Based on the MOOSE guidelines.

Instructions to authors

Complete this checklist by entering the page numbers from your manuscript where readers will find each of the items listed below.

Your article may not currently address all the items on the checklist. Please modify your text to include the missing information. If you are certain that an item does not apply, please write "n/a" and provide a short explanation.

Upload your completed checklist as an extra file when you submit to a journal.

In your methods section, say that you used the MOOSE reporting guidelines, and cite them as:

Stroup DF, Berlin JA, Morton SC, Olkin I, Williamson GD, Rennie D, Moher D, Becker BJ, Sipe TA, Thacker SB. Meta-analysis of observational studies in epidemiology: a proposal for reporting. Meta-analysis Of Observational Studies in Epidemiology (MOOSE) group. JAMA. 2000; 283(15):2008-2012.

	Reporting Item	Page Number
#1	Identify the study as a meta-analysis of observational research	2
#2	Provide a structured summary including, as applicable: background; objectives; data sources; study eligibility criteria, participants, and interventions; study appraisal and synthesis methods; results; limitations; conclusions and implications of key findings; systematic review registration number (From PRISMA checklist)	2-4
#3a	Problem definition	7
#3b	Hypothesis statement	n/a
#3c	Description of study outcomes	8
#3d	Type of exposure or intervention used	8
#3e	Type of study designs used	8

For peer review only

	#3f	Study population	8
Search strategy	#4a	Qualifications of searchers (eg, librarians and investigators)	24
	#4b	Search strategy, including time period included in the synthesis and keywords	9-10, Suppl. 2-3, Table 1
	#4c	Effort to include all available studies, including contact with authors	10
	#4d	Databases and registries searched	9
	#4e	Search software used, name and version, including special features used (eg, explosion)	Suppl. 2, Table 1
	#4f	Use of hand searching (eg, reference lists of obtained articles)	9
	#4g	List of citations located and those excluded, including justification	n/a, word count restriction
	#4h	Method of addressing articles published in languages other than English	9
	#4i	Method of handling abstracts and unpublished studies	9
	#4j	Description of any contact with authors	10
	#5a	Description of relevance or appropriateness of studies gathered for assessing the hypothesis to be tested	12-17
	#5b	Rationale for the selection and coding of data (eg, sound clinical principles or convenience)	9-10
	#5c	Documentation of how data were classified and coded (eg, multiple raters, blinding, and interrater reliability)	9-10
	#5d	Assessment of confounding (eg, comparability of cases and controls in studies where appropriate)	10
	#5e	Assessment of study quality, including blinding of quality assessors; stratification or regression on possible predictors of study results	10
	#5f	Assessment of heterogeneity	10
	#5g	Description of statistical methods (eg, complete description of fixed or random effects models, justification of whether the chosen	10-11

For peer review only

	models account for predictors of study results, dose-response models, or cumulative meta-analysis) in sufficient detail to be replicated	
#5h	Provision of appropriate tables and graphics	Figure 1,2, p13-14, Table 1, p18 Table 2, Suppl. all Tables
#6a	Graphic summarizing individual study estimates and overall estimate	18, Table 2, suppl. 9-11, Table 5-9
#6b	Table giving descriptive information for each study included	13-14, Table 1
#6c	Results of sensitivity testing (eg, subgroup analysis)	17-18, Table 2
#6d	Indication of statistical uncertainty of findings	18, Table 2
#7a	Quantitative assessment of bias (eg. publication bias)	10, 17-18
#7b	Justification for exclusion (eg, exclusion of non–English-language citations)	9
#7c	Assessment of quality of included studies	16-17, suppl. 7-8, Table 3-4
#8a	Consideration of alternative explanations for observed results	21
#8b	Generalization of the conclusions (ie, appropriate for the data presented and within the domain of the literature review)	22-23
#8c	Guidelines for future research	23
#8d	Disclosure of funding source	25

Reproduced with permission from JAMA. 2000. 283(15):2008-2012. Copyright © 2000 American Medical Association. All rights reserved.This checklist can be completed online using https://www.goodreports.org/, a tool made by the EQUATOR Network in collaboration with Penelope.ai

For peer review only
